# Efficacy and safety of L-oxiracetam on cognitive function in patients with traumatic brain injury: a multicentre, randomised, double-blind, phase 3 clinical trial

**DOI:** 10.1038/s41392-025-02492-5

**Published:** 2025-12-12

**Authors:** Tao Liu, Jiao Wang, Zhihao Zhao, Weiwei Jiang, Minzhi Zhang, Yunhu Yu, Yang Liu, Mingqi Liu, Linan Chen, Hengzhu Zhang, Yingbiao Hong, Bohe Li, Rutong Yu, Hongming Ji, Liang Mi, Biao Zhao, Chuanxiang Lv, Chenglong Liu, Jianning Zhang, Rongcai Jiang

**Affiliations:** 1 Department of Neurosurgery, Xuanwu Hospital, Capital Medical University, Beijing, China; 2 Department of Neurosurgery, Tianjin Medical University General Hospital, Tianjin, China; 3 The George Institute for Global Health, Faculty of Medicine, University of New South Wales, Sydney, NSW Australia; 4 Center of Gerontology and Geriatrics, West China Hospital, Sichuan University, Chengdu, China; 5 National Clinical Research Centre for Geriatrics, West China Hospital, Sichuan University, Chengdu, China; 6 Department of Neurobiology, Care Sciences and Society, Karolinska Institutet, Stockholm, Sweden; 7 Department of Rehabilitation Medicine, Zhujiang Hospital, Southern Medical University, Guangzhou, China; 8 Department of Neurology, Tianjin Medical University General Hospital, Tianjin, China; 9 Department of Clinical Research Centre for Neurological Disease, The People’s Hospital of Honghuagang District of ZunYi, Guizhou, China; 10 Department of Rehabilitation Medicine, Zhejiang Provincial People’s Hospital, Hangzhou, China; 11 Department of Neurosurgery, Subei People’s Hospital, Jiangsu, China; 12 Department of Neurosurgery, Jieyang People’s Hospital, Guangdong, China; 13 Department of Neurosurgery, Yichun People’s Hospital, Jiangxi, China; 14 Department of Neurosurgery, The Affiliated Hospital of Xuzhou Medical University, Xuzhou, China; 15 Department of Neurosurgery, Shanxi Provincial People’s Hospital, Shanxi, China; 16 Department of Neurosurgery, The Second Affiliated Hospital of Bengbu Medical University, Bengbu, China; 17 Department of Neurosurgery, Tianjin Huanhu Hospital, Tianjin, China; 18 Department of Radiology, The First Affiliated Hospital of Anhui Medical University, Hefei, China; 19 Yan’an Hospital of Kunming City, Kunming, China; 20 Dazhu County People’s Hospital, Dazhou, China; 21 The People’s Hospital of Yuechi County, Guang’an, China; 22 Yan’an University Xianyang Hospital, Xianyang, China; 23 Pu’er City People’s Hospital, Pu’er, China; 24 Panzhihua Central Hospital, Panzhihua, China; 25 First Affiliated Hospital of Anhui Medical University North District, Hefei, China; 26 The First People’s Hospital of Nanning, Nanning, China; 27 Huai’an NO.1 People’s Hospital of Nanjing Medical University, Huai’an, China; 28 Yangquan Coal Industry General Hospital, Yangquan, China; 29 The First People’s Hospital of Jinzhong, Jinzhong, China; 30 Zhumadian Central Hospital, Zhumadian, China; 31 The First People’s Hospital of Lianyungang, Lianyungang, China; 32 The Second Affiliated Hospital of Nanchang University, Nanchang, China; 33 The First People’s Hospital of Changzhou, Changzhou, China; 34 The First People’s Hospital of Shenyang, Shenyang, China; 35 Inner Mongolia Autonomous Region People’s Hospital, Hohhot, China; 36 Zhangzhou Municipal Hospital of Fujian Province, Zhangzhou, China; 37 Haikou People’s Hospital, Haikou, China; 38 Liuzhou Worker’s Hospital, Liuzhou, China; 39 Taizhou First People’s Hospital, Taizhou, China; 40 Ganzhou People’s Hospital, Ganzhou, China; 41 Beijing Chaoyang Hospital, Beijing, China; 42 First Affiliated Hospital of Gannan Medical University, Ganzhou, China; 43 Hainan Third People’s Hospital, Sanya, China; 44 Suzhou Kowloon Hospital, Suzhou, China; 45 Affiliated Hospital of Nantong University, Nantong, China; 46 Affiliated Hospital of Jiangsu University, Zhenjiang, Jiangsu China; 47 The First People’s Hospital of Changde City, Changde, China; 48 Linfen People’s Hospital, Linfen, China; 49 Guilin People’s Hospital, Guilin, China; 50 First Affiliated Hospital of Ningbo University, Ningbo, China; 51 Affiliated Hospital of Henan University of Science and Technology, Luoyang, China; 52 The Second Affiliated Hospital of Guangzhou Medical University, Guangzhou, China; 53 Huizhou Central People’s Hospital, Huizhou, China; 54 Zhejiang Provincial People’s Hospital, Hangzhou, China; 55 The First Affiliated Hospital of Nanchang University, Nanchang, China; 56 The Second Hospital of Tianjin Medical University, Tianjin, China

**Keywords:** Trauma, Neurology, Neurological disorders

## Abstract

To assess the efficacy and safety of L-oxiracetam, a novel nootropic agent, in improving cognition in patients with TBI, we performed a multicentre, double-blind, randomized controlled trial in China. Participants aged 18 to 75 years with TBI (Glasgow Coma Scale score of 10 to 15) were recruited from 51 hospitals from 2019 to 2024. Patients were randomly assigned to L-oxiracetam, 4 g/day, oxiracetam 6 g/day, or placebo in 2:2:1. The primary outcome was the change in the Loewenstein Occupational Therapy Cognitive Assessment (LOTCA) score from baseline to 90 days post treatment. Secondary outcomes included changes in additional cognitive evaluations, neurological function, activities of daily living (ADL), and adverse events (AEs). The trial was approved by the China National Medical Products Administration (2016L03521), and registered at Clinicaltrials.gov (NCT04205565) and Chinadrugtrials.org.cn (CTR20192539). Five hundred and ninety patients were included (mean age (SD), 50.9 (14.5); 421 males). The least squares (LS) mean of LOTCA change from baseline to 90 days post treatment was 20.45 (95% confidence interval [CI] 17.23, 23.66) in the L-oxiracetam group, 15.90 (95% CI 12.71, 19.10) in the oxiracetam group, and 11.47 (95% CI 7.75, 15.20) in the placebo group (*P* value < 0.05 for all groups). The LS mean difference of the L-oxiracetam was significantly higher than the placebo group (8.97, 95% CI 5.69,12.26; *P* < 0.001; Cohen’s *d* = 0.48 [95% CI: 0.26,0.69]) and the oxiracetam group (4.54, 95% CI 1.85,7.23). Secondary efficacy outcomes did not differ between the L-oxiracetam and oxiracetam groups. The proportion of serious AEs did not differ among the three groups. L-oxiracetam could improve cognitive function in patients with mild-to-moderate TBI. L-oxiracetam might be more efficacious than oxiracetam. No significant safety concerns were reported. Despite limitations such as loss to follow-up, the findings of this study provide important evidence for the clinical management of cognitive dysfunction following TBI. Future studies in real-world clinical settings are warranted to further substantiate the efficacy of L-oxiracetam and oxiracetam.

## Introduction

Traumatic brain injury (TBI) is a major global health concern, imposing a substantial burden on individuals, healthcare systems, and societies. Between 1990 and 2016, the incidence of TBI increased by 47%.^1^ A global survey in 2018 estimated that TBI affects ~69 million people worldwide each year.^2^ Despite a steady decline in mortality over the past several decades, the disability rate remains high, with more than 8 million people suffering from severe disability annually.^2,3^ Among survivors, cognitive dysfunction remains a major sequela. Approximately 15–25% of patients with mild TBI experience long-term cognitive impairment, characterized by deficits in attention, memory, and executive function.^4,5^ A recent large-scale cohort study (the Transforming Research and Clinical Knowledge in TBI study) found that cognitive impairment is more frequent among patients with more severe TBI and exhibits considerable heterogeneity across individuals.^6^ Meta-analyzes and systematic reviews have further confirmed that long-term cognitive impairment is common even after mild TBI, particularly among patients with positive brain imaging.^7,8^ Moreover, TBI has also been linked to an increased lifetime risk of developing Alzheimer’s disease and other forms of dementia.^9^

Putative mechanisms underlying cognitive impairment after TBI include structural damage to relevant brain regions and abnormalities in neurotransmitters and their associated receptors.^4^ TBI typically results in focal contusion and diffuse axonal injury (DAI). DAI serves as one of the primary pathological bases for cognitive impairment, characterized by axonal disruption, impaired axonal transport, and demyelination.^10^ These pathological changes result in the disruption of cortical-subcortical network connectivity, particularly affecting key regions such as the frontal lobe, hippocampus, thalamus, and corpus callosum. Neuroimaging studies have revealed significantly reduced connectivity within the default mode network and the frontoparietal network in TBI patients, which is closely associated with deficits in attention and working memory.^11^ In addition, dysfunction in multiple neurotransmitter systems is frequently observed following TBI, including the cholinergic, glutamatergic, dopaminergic, and GABAergic pathways.^12^ Among these, cholinergic pathway disruption, particularly involving cholinergic neurons in the hippocampus and prefrontal cortex, is closely linked to impairments in attention, learning, and memory.^13,14^ Experimental studies have demonstrated that reduced acetylcholine release is directly associated with impaired synaptic plasticity.^15^ Furthermore, chronic inflammatory responses and mitochondrial dysfunction further impair synaptic plasticity and neurogenesis.^16–19^ The interplay of these multifaceted factors ultimately culminates in cognitive dysfunction following TBI.

Treatments intended to improve cognitive function after TBI include edaravone dexborneol, oxiracetam, piracetam, and compound porcine cerebroside and ganglioside injections.^20–22^ The efficacy of these early pharmacological interventions, however, lacks support from high-quality evidence, such as randomized controlled trials (RCTs), meta-analyzes, and clinical practice guidelines.

The nootropic agent oxiracetam promotes the reorganization and functional recovery of damaged brain tissue through multiple mechanisms, thereby enhancing cognitive function. A study in mice showed that oxiracetam activates cholinergic nerve fibers in the cerebral cortex and hippocampus and increases the release of acetylcholine, which in turn promotes synaptic plasticity.^23^ Oxiracetam has also been shown to reduce neuronal damage by scavenging free radicals, mitigating oxidative stress, and improving brain energy metabolism by activating the adenosine triphosphate synthesis.^24^ Recent evidence indicates that oxiracetam may ameliorate neuroinflammation during the early phase of TBI, mitigating neuronal injury and improving learning, memory, and spatial cognition in animal models.^25,26^ Animal and clinical studies suggest that compared to racemic oxiracetam, L-oxiracetam has a lower clearance rate and a longer half-life, reduces neuroinflammation and increases cerebral blood flow.^27,28^ However, currently available results come from small sample studies on cognitive impairments caused by other diseases (e.g., Alzheimer’s disease, vascular dementia) and lack sufficient scientific evidence to support clinical use of oxiracetam and its active component L-oxiracetam to improve cognitive function after TBI.^29,30^ We recently completed a RCT to examine the efficacy and safety of L-oxiracetam and to assess whether L-oxiracetam provides superior cognitive outcomes at 90 days compared to oxiracetam in patients with mild-to-moderate TBI.

## Results

### Study population

**Fig. 1:**
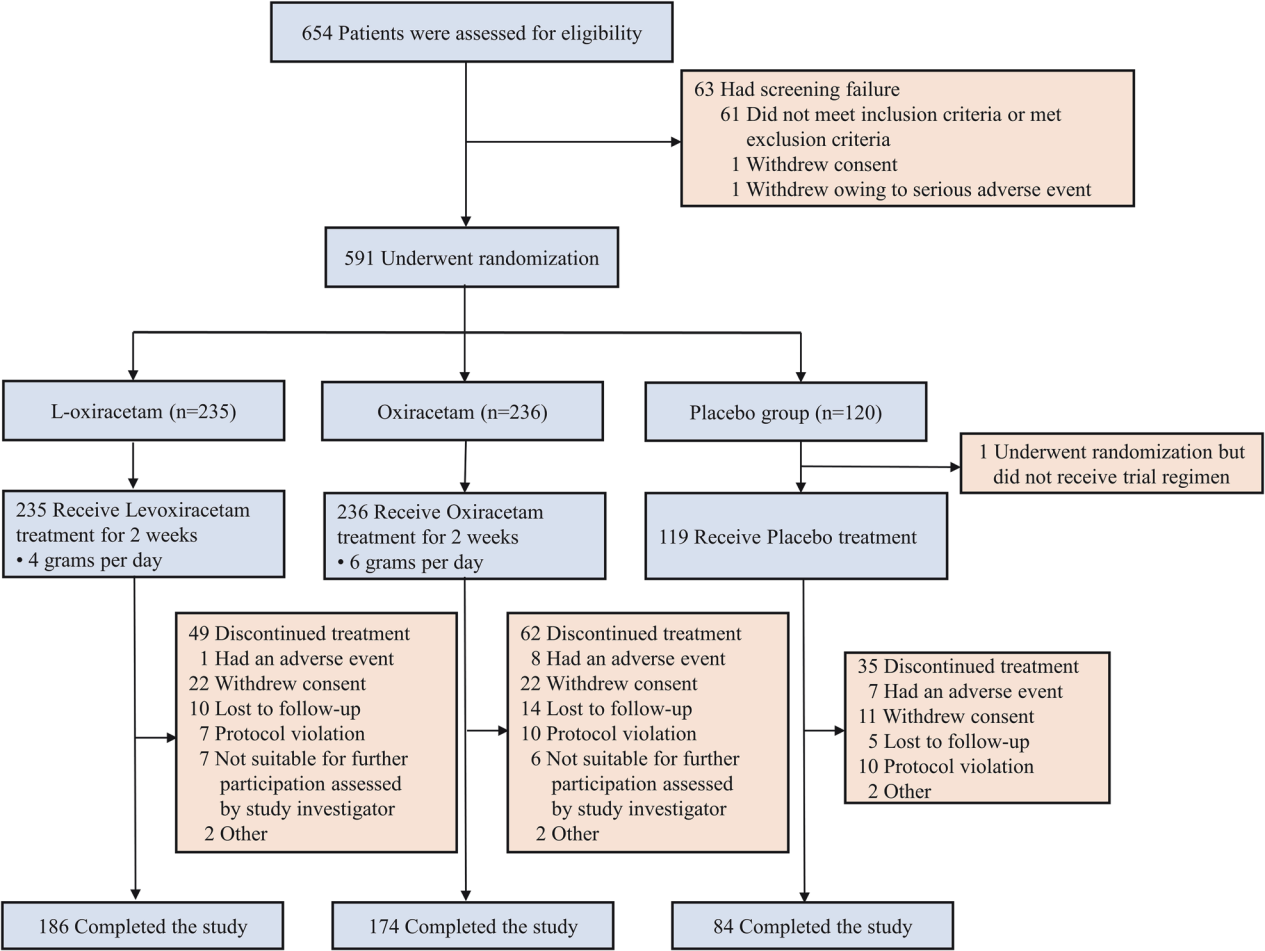
Patient flow through the trial. Flowchart illustrating the enrollment, screening, randomization, intervention, and follow-up processes for participants in this trial

From September 2019 to May 2024, a total of 590 patients were enrolled: 235 were assigned to the L-oxiracetam group, 236 to the oxiracetam group, and 119 to the placebo group (Fig. 1). A total of 146 patients withdrew before the 90-day assessment (49 from the L-oxiracetam group, 62 from the oxiracetam group, and 35 from the placebo group), leaving 444 patients (186 in the L-oxiracetam group, 174 in the oxiracetam group, and 84 in the placebo group) in the per-protocol analysis. Demographic and baseline characteristics of the Intent-to-Treat (ITT) population are presented in Table 1. No significant differences were observed among the three groups. Characteristics of the per-protocol population are shown in the Supplementary Table 1. Characteristics of the patients in each study site are shown in the Supplementary Table 2. The characteristics of patients who completed the follow-up and those who did not are summarized in the Supplementary Table 3. Additionally, we conducted a multivariate analysis to explore the types of loss to follow-up in different groups. The results showed that the loss to follow-up is highly likely to be random and is not influenced by treatment allocation (Supplementary Table 4).

**Table 1:** Demographic and baseline characteristics of the Intent-to-treat population

Characteristics	L-oxiracetam (*n* = 235)	Oxiracetam (*n* = 236)	Placebo (*n* = 119)	Overall* (*n* = 590)
Age, mean (SD), yr	50.5 (14.70)	51.2 (14.98)	51.0 (12.88)	50.9 (14.45)
<65, *n* (%)	184 (78.30)	182 (77.12)	105 (88.24)	471 (79.83)
≥65, *n* (%)	51 (21.70)	54 (22.88)	14 (11.79)	119 (20.17)
Sex, *n* (%)				
Male	162 (68.94)	172 (72.88)	87 (73.11)	421 (71.36)
Female	73 (31.06)	64 (27.12)	32 (26.89)	169 (28.64)
Race, *n* (%)				
Han nationality	222 (94.47)	222 (94.07)	112 (94.12)	556 (94.2)
Minorities	13 (5.53)	14 (5.93)	7 (5.88)	34 (5.76)
Education level, *n* (%)				
None	22 (9.36)	21 (8.90)	12 (10.08)	55 (9.32)
Primary school	73 (31.06)	75 (31.78)	38 (31.93)	186 (31.52)
Secondary school or higher	140 (59.58)	140 (59.32)	69 (57.98)	349 (59.15)
Drug allergy history, *n* (%)	15 (6.38)	8 (3.39)	3 (2.52)	26 (4.41)
Body temperature, mean (SD)	36.7 (0.29)	36.6 (0.36)	36.6 (0.30)	36.6 (0.32)
Respiratory rate, mean (SD)	18.9 (1.58)	18.7 (1.61)	18.9 (1.70)	18.8 (1.62)
Heart rate, mean (SD)	76.0 (11.65)	76.1 (10.60)	75.9 (12.45)	76 (11.39)
Blood pressure, mean (SD)				
Systolic	125.9 (14.88)	125.6 (15.25)	124.0 (14.13)	125.4 (14.87)
Diastolic	76.2 (10.29)	75.5 (10.03)	76.3 (9.95)	75.9 (10.11)
QT interval^†^, mean (SD)	382.5 (36.71)	388.3 (36.10)	386.8 (32.27)	385.7 (35.64)
Cause of injury, *n* (%)				
Road-traffic incident	89 (37.87)	98 (41.53)	46 (38.66)	233 (39.49)
Incidental fall				
Ground-level fall	39 (16.60)	58 (24.58)	23 (19.32)	120 (20.34)
Fall from height	65 (27.66)	54 (22.88)	34 (28.57)	153 (25.93)
Mechanism of injury, *n* (%)				
Primary injury	192 (81.70)	167 (70.76)	91 (76.47)	450 (76.27)
Secondary injury	9 (3.83)	12 (5.08)	7 (5.88)	28 (4.75)
Unknown	34 (14.47)	57 (24.15)	21 (17.64)	112 (18.98)
TBI severity, *n* (%)				
Mild	221 (94.04)	221 (93.64)	112 (94.12)	554 (93.90)
Moderate	14 (5.96)	15 (6.36)	7 (5.88)	36 (6.10)
Surgical treatment, *n* (%)	5 (2.13)	3 (1.27)	1 (0.84)	9 (1.52)
Testing scores at baseline				
LOTCA, mean (SD)	71.8 (22.06)	72.3 (22.03)	71.4 (21.98)	71.9 (22.00)
MoCA, mean (SD)	14.4 (6.22)	15.2 (6.37)	14.1 (6.16)	14.6 (6.28)
MMSE, mean (SD)	19.1 (5.60)	19.5 (5.64)	18.6 (5.31)	19.2 (5.56)
GCS, median (IQR)	15.0 (14.00–15.00)	15.0 (14.00–15.00)	15.0 (14.00–15.00)	15.0 (14.0–15.0)

*GCS* Glasgow Coma Scale, *LOTCA* Loewenstein Occupational Therapy Cognitive Assessment, *MMSE* mini-mental state examination, *MoCA* Montreal Cognitive Assessment, *TBI* traumatic brain injury, *SD* standard deviation

^†^QT interval, the length of the QT interval is related to the heart rate. Abnormalities in the QT interval may be associated with cardiac diseases, especially those that can lead to arrhythmias

^*^No significant differences were observed among the three groups

### Primary outcome

**Fig. 2:**
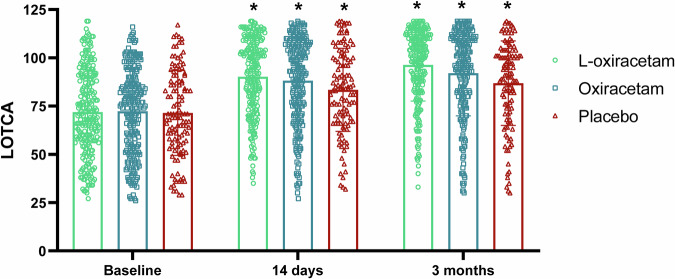
Column chart of LOTCA scores at baseline, 14 days, and 90 days. The LOTCA scores at days 14 and 90 were significantly higher than baseline in all 3 groups (L-oxiracetam, Oxiracetam, and Placebo). *There was a significant increase in LOTCA compared with the baseline. LOTCA Loewenstein Occupational Therapy Cognitive Assessment

The LOTCA score at 90 days was significantly higher than baseline in all 3 groups (Fig. 2). The LS mean of LOTCA change from baseline to 90 days post treatment was 20.45 (95% CI: 17.23, 23.66) in the L-oxiracetam group, 15.90 (12.71, 19.10) in the oxiracetam group, and 11.47 (7.75, 15.20) in the placebo group (Table 2; Supplementary Table 5). The LS mean difference was 8.97 (95% CI: 5.69, 12.26; *P* < 0.001) between the L-oxiracetam and placebo groups and 4.54 (1.85, 7.23) between the L-oxiracetam and oxiracetam groups. Changes in individual items of LOTCA score at 90 days are shown in the Supplementary Table 6. The LS mean of change of the L- oxiracetam group was significantly greater than the control group in visual perception, spatial perception, motor praxis, visual motor organization, thinking operation, attention and concentration (all *P value* < 0.05).

**Table 2:** Group differences of the primary and secondary outcomes

End point^a^	L-oxiracetam	Oxiracetam	Placebo	L-oxiracetam vs. Placebo			L-oxiracetam vs. Oxiracetam
				Mean Difference (95% CI)^b^	Effect Size (95% CI)^b^	*P*	Mean Difference (95% CI)^b, c^
Primary outcome							
Mean change in LOTCA at 90 days	20.45 (17.23, 23.66)	15.90 (12.71, 19.10)	11.47 (7.75, 15.20)	8.97 (5.69, 12.26)	0.48 (0.26, 0.69)	<0.001	4.54 (1.85, 7.23)
Secondary outcomes							
Mean change in LOTCA at 14 days	13.76 (10.86, 16.66)	11.47 (8.59, 14.35)	7.45 (4.09, 10.80)	6.31 (3.35, 9.28)	0.39 (0.18, 0.60)	<0.001	2.29 (–0.14, 4.71)
Mean change in MMSE							
14 days	4.47 (3.67, 5.27)	4.23 (3.43, 5.02)	4.53 (3.59, 5.46)	–0.05 (–0.86, 0.75)	–0.03 (–0.25, 0.18)	0.896	0.25 (–0.41, 0.90)
90 days	5.62 (4.70, 6.53)	5.14 (4.22, 6.06)	4.91 (3.87, 5.94)	0.71 (–0.16, 1.57)	0.13 (–0.10, 0.37)	0.108	0.48 (–0.23, 1.18)
Mean change in MoCA							
14 days	4.09 (3.22, 4.97)	3.38 (2.52, 4.23)	3.27 (2.25, 4.29)	0.83 (–0.07, 1.72)	0.22 (0.00, 0.43)	0.072	0.72 (–0.01, 1.45)
90 days	5.47 (4.39, 6.55)	4.77 (3.70, 5.84)	4.37 (3.15, 5.58)	1.11 (0.08, 2.14)	0.26 (0.03, 0.50)	0.036	0.71 (–0.14, 1.55)
Percentage of GOSE 7–8, *n* (%)							
14 days	90.42 (85.80, 93.90)	92.21 (87.90, 95.40)	92.72 (86.00, 96.80)	–2.30 (–8.50, 3.90)	–0.08 (–0.29, 0.13)	0.546	–1.80 (–7.10, 3.40)
90 days	97.43 (94.10, 99.20)	97.31 (93.80, 99.10)	98.92 (94.20, 100.00)	–1.50 (–4.61, 1.51)	–0.11 (–0.34, 0.13)	0.667	0.10 (–3.10, 3.30)
ADL							
14 days	77.59 (73.39, 81.79)	75.42 (71.21, 79.63)	76.83 (71.81, 81.86)	0.76 (–3.94, 5.45)	0.05 (–0.17, 0.26)	0.751	2.17 (–1.64, 5.98)
30 days	92.64 (90.67, 94.62)	92.04 (90.06, 94.02)	92.50 (90.13, 94.88)	0.14 (–2.09, 2.37)	0.02 (–0.19, 0.24)	0.909	0.60 (–1.20, 2.40)
60 days	96.56 (95.21, 97.90)	95.94 (94.59, 97.29)	96.74 (95.12, 98.37)	–0.19 (–1.71, 1.34)	–0.03 (–0.24, 0.19)	0.810	0.61 (–0.62, 1.84)
90 days	98.39 (97.30, 99.49)	98.16 (97.04, 99.28)	98.58 (97.31, 99.85)	–0.19 (–1.33, 0.96)	–0.03 (-0.26, 0.21)	0.747	0.23 (–0.70, 1.17)
Change in GCS at 14 days	0.49 (0.40, 0.58)	0.46 (0.37, 0.55)	0.50 (0.40, 0.60)	–0.01 (–0.08, 0.07)	–0.04 (–0.26, 0.17)	0.872	0.03 (–0.03, 0.09)

*LOTCA* Loewenstein Occupational Therapy Cognitive Assessment, *MMSE* mini-mental state examination, *MoCA* Montreal Cognitive Assessment, *GOSE* Extended Glasgow Outcome Scale, *GCS* Glasgow Coma Scale, *CI* confidence interval, *IQR* interquartile range, *ADL* activities of daily living

^a^The endpoint was assessed at 14 days (end of the treatment), 30 days (first follow-up), 60 days (second follow-up), and 90 days (third follow-up) after the end of treatment. The analysis compared the outcomes assessed at corresponding date with baseline

^b^Adjusted for age, sex, education, TBI severity, and measurements of each outcome at baseline

^c^The confidence intervals have not been adjusted for multiplicity and cannot be used to infer treatment effects

The results of per-protocol analysis are generally consistent with those in the ITT population (Supplementary Table 7 for individual items of LOTCA; Supplementary Table 8 for unadjusted analysis; and Supplementary Table 9 for adjusted analysis). In the per-protocol population, the LS mean difference for L-oxiracetam was significantly greater than that of both the placebo and oxiracetam groups at day 90, showing a similar direction and magnitude of effect as observed in the ITT population.

### Secondary outcomes

None of the secondary outcomes differed significantly between the L-oxiracetam and oxiracetam groups (Table 2). However, compared with the placebo group, a significantly greater increase in LOTCA score at 14 days was observed in the L-oxiracetam group (mean difference 6.31 [95% CI: 3.35, 9.28]; *P* < 0.001) (Table 2). The L-oxiracetam group also showed a greater increase in MoCA score at 90 days compared with the placebo group (mean difference 1.11 [95% CI: 0.08, 2.14]).

There were no significant differences between the L-oxiracetam and placebo groups in MMSE score change at either 14 days (mean difference –0.05 [95% CI: –0.86, 0.75]) or 90 days (mean difference 0.71 [95% CI: –0.16, 1.57]), MoCA score change at 14 days (mean difference 0.83 [95% CI: –0.07, 1.72]), Glasgow Coma Scale (GCS) score change at 14 days (mean difference –0.01 [95% CI: –0.08, 0.07]), the proportion of patients with GOS-E score of 7–8 at either 14 or 90 days, or activities of daily living (ADL) scores at multiple time points (days 14, 30, 60, and 90) (Table 2).

The results for individual items of MMSE, MoCA, and GCS are shown in the Supplementary Tables 10–12, with no significant differences among the L-oxiracetam and placebo groups. The results of the secondary outcomes in the per-protocol analysis are consistent with the main analysis (Supplementary Tables 8 and 13–15). The results of sensitivity analyzes after imputation of missing values for both primary and second outcomes are consistent with the main analysis (Supplementary Table 16).

### Subgroup analyses

**Fig. 3:**
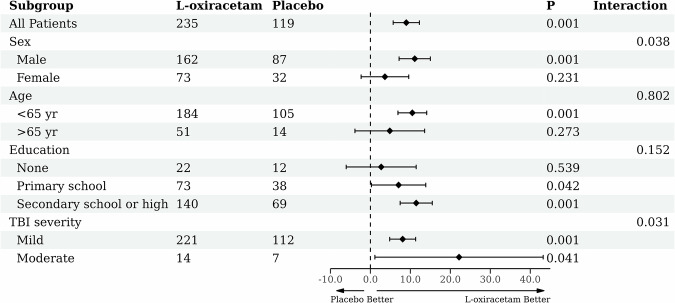
Subgroup analysis of the primary outcome (mean change in LOTCA at 90 days). Models adjusted for age, sex, education, TBI severity, and LOTCA at baseline. There were significant interactions between L-oxiracetam treatment and sex and TBI severity. yr year, TBI Traumatic brain injury

The results of subgroup analyses based on sex, age, education and TBI severity are presented in Fig. 3. There were significant interactions between L-oxiracetam treatment and sex (*P* for interaction = 0.038) and TBI severity (*P* for interaction = 0.031). Significant changes in LOTCA scores from baseline to day 90 were observed between the L-oxiracetam and placebo groups in male (mean difference [MD] 11.09, 95% CI [7.17, 15.00]; *P* < 0.0001; Supplementary Table 16) but not in female (MD 3.63, 95% CI [–2.33, 9.59]; *P* = 0.2305). Participants with moderate TBI severity (MD 22.18, 95% CI [1.12, 42.23]; *P* = 0.0397) appeared to be more responsive to L-oxiracetam compared to those with mild TBI severity (MD 8.07, 95% CI [4.82, 11.32]; *P* < 0.0001). No interactions between L-oxiracetam treatment and age or education were observed (both *P* for interaction > 0.05; Supplementary Table 17).

In additional exploratory subgroup analyses, the change in LOTCA score from baseline to day 90 was greater in the L-oxiracetam group than in the placebo group in the subgroup of patients with traffic incidents, primary injury, and any time interval from injury to first medication (all *P* < 0.05; Supplementary Table 18).

### AEs

AE of any grades occurred in 155 (65.96%) patients in the L-oxiracetam group, 157 (66.53%) in the oxiracetam group, and 80 (67.23%) in the placebo group (*P* = 0.980). The rate of SAEs was 13 (5.53%) in the L-oxiracetam group, 18 (7.63%) in the oxiracetam group, and 15 (12.60%) in the placebo group (*P* = 0.100). Oxiracetam might have a higher rate of overall treatment-related AEs (22 [9.4%] vs 41 [17.4%] vs 11(9.2); *P* = 0.049). Details are listed in Table 3.

**Table 3:** Frequencies of adverse events by treatment group

Adverse event	L-oxiracetam (*N* = 235)	Oxiracetam (*N* = 236)	Placebo (*N* = 119)	*P*
Any adverse event	155 (65.96)	157 (66.53)	80 (67.23)	0.980
Serious adverse event	13 (5.53)	18 (7.63)	15 (12.60)	0.100
Treatment-related serious adverse event	0 (0.00)	0 (0.00)	0 (0.00)	–
Adverse event leading to dose reduction	0 (0.00)	0 (0.00)	0 (0.00)	–
Adverse event leading to permanent discontinuation of treatment	1 (0.43)	9 (3.81)	7 (5.88)	0.004
Adverse event leading to death	0 (0.00)	0 (0.00)	1 (0.84)	0.203
Most common treatment-related adverse events	2 (0.85)	6 (2.54)	7 (5.88)	0.021
Treatment-related adverse event	22 (9.36)	41 (17.37)	11 (9.24)	0.049
Abnormal laboratory examination	16 (6.81)	23 (9.75)	4 (3.36)	0.085
Systemic diseases and reactions at the site of administration	2 (0.85)	6 (2.54)	7 (5.88)	0.649
Gastrointestinal diseases	0 (0.00)	6 (2.54)	0 (0.00)	0.017
Electrolyte disturbances	1 (0.43)	5 (2.12)	1 (0.84)	0.294
Heart disease, *n*/*N* (%)	2 (0.85)	1 (0.42)	0 (0.00)	0.615
Renal disease	0 (0.00)	1 (0.42)	0 (0.00)	1.000
Liver disease	1 (0.43)	0 (0.00)	0 (0.00)	0.600
Nervous system disease	0 (0.00)	4 (1.70)	1 (0.84)	0.129
Infection	0 (0.00)	1 (0.42)	0 (0.00)	1.000
Mental illness	2 (0.85)	1 (0.42)	1 (0.84)	0.845
Skin and subcutaneous tissue diseases	0 (0.00)	5 (2.12)	0 (0.00)	0.026
Respiratory system disease	0 (0.00)	1 (0.42)	0 (0.00)	1.000
Immune system disorders	0 (0.00)	2 (0.85)	0 (0.00)	0.358

Numbers represent the *n* (%) of participants who experienced the given adverse event at least once during the follow-up. Data are the *n* (%) of patients who experienced the given adverse event at least once during the follow-up

## Discussion

This trial demonstrated an improved LOTCA score in the L-oxiracetam group at 90 days compared to both the oxiracetam and placebo groups. There was no significant difference in the overall rate of AEs among the three groups. However, the rate of treatment-related AEs was significantly lower in the L-oxiracetam group than in the oxiracetam group.

Currently, there is a lack of specific and effective early interventions to improve cognitive functions and support vocational rehabilitation after TBI. This is particularly evident in patients with mild-to-moderate TBI, who may experience long-term cognitive impairments, emotional instability, and sensory and motor disorders, leading to significant distress for both individuals and their families.^31^ In animal studies, oxiracetam has been shown to improve memory in rats with concussion-induced brain injuries by alleviating blood-brain barrier (BBB) injury and increasing cerebral blood flow.^25,32^ In a clinical study, as the major active enantiomer of oxiracetam, L-oxiracetam has demonstrated good tolerance in Chinese healthy volunteers, with favorable pharmacokinetic profiles and relatively few adverse reactions.^28^

Oxiracetam was approved for the treatment of vascular dementia in China in 2003.^33^ Although its efficacy and safety had not been fully evaluated at the time of approval, several preclinical studies had been conducted. Several placebo-controlled trials have since found that oxiracetam can alleviate physical and mental symptoms in patients with primary degenerative and multi-infarct dementia and, to some extent, improve cognitive impairments in these patients.^34,35^ In addition, oxiracetam has also been used in the treatment of dementia of the Alzheimer type.^30,36^ Taken together, to date, oxiracetam has been explored for dementia and cognitive impairment of various aetiologies, including vascular dementia, multi-infarct dementia, and Alzheimer’s disease. The mechanisms underlying the cognitive effects of oxiracetam may include activation of central cholinergic neurons, increase in the synthesis or release of acetylcholine, and increased sensitivity of cholinergic receptors.^4,37^ Furthermore, animal and clinical studies indicate that oxiracetam may help alleviate BBB disruption caused by ischemic stroke and induce a neuroprotective microglial phenotype after hypoxic-ischemic brain injury.^24,38^

To date, there has been a plethora of intervention studies aimed at oxiracetam improving cognitive function in TBI animal models by targeting mitochondrial functions, inflammation, oxidative stress, and brain metabolite metabolism.^39,40^ These studies increase the understanding of the action of oxiracetam on TBI pathophysiology and support the launch of clinical translational research. Up to now, clinical trials focusing on cognitive functions post-TBI have been scarce, and deficiencies in aspects such as sample size and power calculation challenge the rigor, reasonableness, and completeness of these trials.^41^ The current study overcame some of the deficiencies observed in previous trials by incorporating key clinical trial elements such as randomization, blinding, and controls, predefining endpoint indicators and statistical analysis plans, and having the statistical analysis conducted independently by a third party, thereby enhancing the standardization and credibility of the experimental results.

In our study, only patients with mild-to-moderate TBI were included, and the follow-up period was limited to 90 days. The fact that more than 90% of the patients in this trial had mild TBI might have contributed to the lack of group difference in most secondary efficacy outcomes. And the high proportion of patients with mild TBI likely resulted in a ceiling effect in MMSE and MoCA scores, which substantially limited the sensitivity of these tools to detect further improvement or group differences. In addition, unlike the LOTCA, the MoCA and MMSE—although widely used for cognitive assessment—were not specifically designed for brain-injured patients,^42^ and may therefore be less sensitive in detecting subtle cognitive changes in patients with mild TBI.

This trial has several limitations. First, a relatively high proportion of patients withdrew from the study for a variety of reasons, which may introduce the uncertainty of the conclusions. To further address this concern, we conducted a supplementary analysis comparing baseline characteristics and withdrawal patterns between groups. The results indicated that loss to follow-up was not associated with treatment assignment and appeared to be random (Supplementary Tables 3 and 4). In addition, sensitivity analyses of per-protocol population also confirmed the stability of our conclusions. Second, the primary outcome was assessed at 3 months post injury, which may have underestimated the rate of good recovery, as some deficits may persist or emerge beyond this period—particularly when using global functional measures such as the GOSE. Moreover, due to the restricted follow-up duration, we were unable to assess the association between short-term cognitive improvement (3 months) and long-term outcomes such as dementia. Future studies are warranted to investigate the effects of oxiracetam and its active component (L-oxiracetam) on long-term cognitive outcomes including dementia after TBI. Third, the effectiveness of blinding was not formally assessed in our study. However, the research team adhered strictly to predefined blinding protocols, ensuring that both patients and assessors remained unaware of participants’ group assignments. In addition, we did not specifically assess, exclude, or control for patients with exaggerated symptom validity or poor effort on cognitive testing. Nonetheless, because patients were randomized and outcomes were independently assessed by blinded evaluators, such potential bias would be expected to be minimal. Fourth, outcomes in the domains of mood, sleep, and headache were not measured in our study, although these were common areas of impairment following mild TBI. Fifth, the practice effects associated with administering cognitive tests multiple times in a short period (baseline, day 14, and day 90) were difficult to avoid. However, this effect should be homogeneous across groups, and therefore, it has a negligible effect on the results.

In patients with mild-to-moderate TBI, both L-oxiracetam and oxiracetam improved cognitive function as assessed by the LOTCA score at 90 days, with no significant safety concerns. L-oxiracetam seemed to be more efficacious than oxiracetam. The findings should be interpreted with caution due to limitations such as loss to follow-up. Further real-world studies are required to confirm the comparative efficacy of L-oxiracetam and oxiracetam.

## Materials and methods

### Study design

The L-oxiracetam Outcomes in Clinical Assessment of Traumatic Brain Injury Effects (LOCATE) trial is a multicenter, phase III RCT. The trial design and statistical analysis plan have been previously published.^43,44^ Details are shown in the clinical trial protocol. The trial protocol was approved by the ethics committees of all participating centers. All patients provided written informed consent. This manuscript adheres to the Consolidated Standards of Reporting Trials (CONSORT) guidelines.^45^

### Participants

For patients admitted within 72 h after injury, investigators assessed enrollment eligibility during the acute phase of TBI recovery. The inclusion criteria of this trial included:

1. Age 18–75 years (inclusive), without sex restriction;
2. Head injury meeting all of the following conditions:

- Clear evidence of head trauma in the current diagnosis, including closed head injury or head injury with cerebrospinal fluid leakage and/or ear or nasal leakage and/or intracranial air accumulation.
- Confirmed by MRI or CT to have intracranial bleeding above the cerebellar tentorium (including cerebral contusion, subarachnoid hemorrhage, extradural hematoma, subdural hematoma, intracerebral hematoma, etc.), with or without transient loss of consciousness.
- Classified as mild to moderate head injury (GCS: 10–15);
- Stable condition within 72 h after head injury, undergoing conservative treatment, not undergoing craniotomy (may have intracranial pressure monitoring without general anesthesia or basal anesthesia).

3) MMSE score below normal, with diagnostic cut-off values depending on different educational levels: illiterate (no education) ≤19 points, elementary school level ≤ 22 points, junior high school and above level ≤ 26 points;
4) Consent from the guardian and/or patient to participate in this clinical trial and signing of the informed consent form.

Key exclusion criteria included a history of severe head trauma, hemorrhagic or ischemic stroke, and structural brain lesions caused by tumors, trauma, or surgery. A complete list of inclusion and exclusion criteria is provided in the clinical trial protocol (pp 39–40).

### Randomization and masking

Eligible patients were randomly assigned in a 2:2:1 ratio to receive an intravenous infusion of L-oxiracetam (4 g per day), oxiracetam (6 g per day), or placebo for 14 consecutive days (Fig. 1). Baseline GCS scores were recorded by trained assessors after informed consent was obtained, typically within 72 h of injury. Patients were classified as moderate TBI (GCS 10–12) or mild TBI (GCS 13–15). Randomization was stratified by TBI severity (GCS scores of 10–12 vs. 13–15) and educational level (illiterate/no formal education, primary education, or secondary education and higher). The randomization sequence was generated by a staff member from the Department of Public Health, Nanjing Medical University (Jiangsu, China), who was not otherwise involved in the trial. Allocation concealment was achieved through a central web response system. Patients, investigators, and outcome assessors were blinded to the treatment allocation. Outcome assessments were conducted by occupational therapists who were blinded to group allocation and were not involved in the delivery of the intervention or control activities. These assessors received training in standardized administration and scoring of the LOTCA and performed evaluations independently. In the event of a serious adverse event (SAE), group allocation was revealed to attending physicians.

### Procedures

This trial employed a randomized, double-blind, double-dummy design. The investigational drug, L-oxiracetam for injection (1 g per vial, calculated as L-oxiracetam), was manufactured by Shenghe (China) Biopharmaceutical Co., Ltd. The comparator drug, oxiracetam for injection (1 g per vial; approval number: H20100040), was manufactured by CSPC Ouyi Pharmaceutical Co., Ltd. The L-oxiracetam placebo was identical in color, odor, and appearance to L-oxiracetam for injection, but contained no active ingredient; the oxiracetam placebo was identical in colour, odour, and appearance to oxiracetam for injection, but contained no active ingredient. Both placebos were manufactured by Shenghe (China) Biopharmaceuticals Co., Ltd. After providing informed consent and randomization, the medication was initiated as soon as possible, typically within 72 h of injury. Research nurses prepared the trial drugs by diluting them in 100–250 mL of 5% glucose injection or 0.9% sodium chloride injection, mixed thoroughly, and administered via standard intravenous infusion without specific requirements for infusion rate. The intervention group received L-oxiracetam (4 vials/day) plus oxiracetam placebo (6 vials/day); the active control group received L-oxiracetam placebo (4 vials/day) plus oxiracetam (6 vials/day); and the placebo group received L-oxiracetam placebo (4 vials/day) plus oxiracetam placebo (6 vials/day). The treatment period lasted for 14 days, during which all patients received inpatient care. After completion of treatment, participants were followed for 3 months. Comprehensive cognitive assessments, including the LOTCA, MoCA, and MMSE, were conducted by investigators on day 14 and day 90.

Management of intracranial pressure and complications was based on current guidelines and has been previously reported.^46,47^ Medications that could potentially affect the efficacy assessment were neither prescribed by physicians nor permitted for use by patients. Details of the prohibited medications and treatments were shown in the clinical trial protocol (p 48).

### Outcomes

The primary outcome was the change in LOTCA score from baseline to 90 days post treatment, with a higher score indicating better cognitive function.^42^ The Simplified Chinese version of LOTCA used in our study consists of seven cognitive domains: Orientation (awareness of time and place), Visual Perception (interpretation of visual information), Spatial Perception (understanding spatial relationships), Praxis (execution of purposeful motor actions), visuomotor organization (integration of visual input with motor output), Thinking Operation (abstract reasoning, categorisation, sequencing, problem-solving), and Attention and Concentration (ability to focus and shift attention).^48^

The secondary efficacy outcomes included changes from baseline to day 14 in LOTCA and GCS scores and changes from baseline to days 14 and 90 in Mini-Mental State Examination (MMSE) and Montreal Cognitive Assessment (MoCA) scores. Neurological function, assessed by the Glasgow Outcome Scale–Extended (GOS-E) at days 14 and 90, and functional independence, measured by the Barthel Index for ADL at days 14, 30, 60, and 90^49^ were also included as secondary efficacy outcomes. Good recovery was defined as GOSE 7–8.

Adverse events (AEs) were classified into definitely related, probably related, possibly related, possibly unrelated, or definitely unrelated to assigned treatment based on the temporal relationship between the onset of the AEs and the use of the study drug, the type of drug reaction, and whether the reaction diminishes, disappears, or reoccurs after discontinuation of the drug. SAEs were defined as AEs that resulted in unplanned or prolonged hospitalization, were life-threatening, or led to permanent or severe disability, functional impairment, or death.

### Statistical analysis

Sample size was estimated based on the results of an unpublished phase II trial: the mean (standard deviation, SD) changes in LOTCA score from baseline to 90 days post intervention were 26.0 ± 11.8 in L-oxiracetam group, 22.8 ± 10.0 in oxiracetam group, and 20.0 ± 11.4 in placebo group, with pooled SDs of 11.0 and 11.6. The threshold for statistical significance (*α*) was set at 0.05, and the statistical power (1–*β*) at 0.8. Accounting for an expected 15% of patients who might not complete the study (dropout), we determined that 590 patients in total were needed: 236 assigned to L-oxiracetam, 236 to oxiracetam, and 118 to placebo. Details of the sample size estimation have been described previously.^43,44^

All efficacy and safety analyses were performed on all patients who received at least one dose of study medication, according to their original group assignment (ITT) Categorical variables are expressed as numbers (%). Continuous variables were expressed according to the distribution of the data, with normally distributed data expressed as mean ± SD and non-normally distributed using median and interquartile range (IQR). A two-sided *P*-value of <0.05 was considered statistically significant. All statistical analyses were performed using SAS (version 9.4).

#### Primary outcome analysis

The primary outcome of LOTCA score change from the baseline to 90 days was analyzed using analysis of covariance (ANCOVA). This method compares the mean change between groups by statistically adjusting for recognized factors that may affect recovery, included age, sex, education level, TBI severity, and LOTCA score at baseline. The average change in LOTCA for each group was reported by the adjusted least squares (LS) means calculated by ANCOVA. Difference in the changes of LOTCA between groups were estimated using mean difference and 95% confidence intervals (CIs) (L-oxiracetam *vs*. oxiracetam/placebo). Efficacy inferences were only performed between the comparison of the L-oxiracetam and the placebo groups. To understand the size of the treatment effect in a standardized way, we calculated Cohen’s d with 95% CI.

To explore whether the effect of L-oxiracetam varied across patient characteristics, multiplicative interactions were tested by including a cross-product item in the model for the intervention group (L-oxiracetam vs. placebo) with age (<60 vs. 60+ years), sex (male vs. female), education level (none vs. primary vs. secondary school or higher), and TBI severity (mild vs. moderate). Then, we performed analyses within specific subgroups based on age, sex, education level, and TBI severity. Additional exploratory subgroup analyses were conducted based on the cause of injury (traffic incident *vs*. ground-level fall *vs*. fall from height), the mechanism of injury (primary *vs*. secondary injury), and the time interval from injury to first medication (≤48 *vs*. >48 to ≤72 *vs*. >72 h).

#### Secondary outcome analysis

For secondary continuous efficacy outcomes, mean differences and 95% CIs were similarly analyzed using ANCOVA. For categorical outcomes GOS-E, the intergroup rate differences and 95% CIs between groups were estimated using the approximate normality method and tested by the Fisher’s exact probability test.

#### Sensitivity analysis

To test how robust our main findings were, we performed a sensitivity analysis including only patients who followed the study protocol closely (Per-Protocol population). We also conducted a post hoc sensitivity analysis to address the possible impact of missing data for outcomes or covariates on the main results. First, the Last Observation Carries Forward method was used to impute the missing values of outcome. Second, we used an advanced statistical technique (multiple imputation using fully conditional specification) to impute the missing covariates. Finally, we repeated the analysis of the efficacy of L-oxiracetam in the imputed complete data.

## Supplementary information

Supplemental materials

Clinical Trial Protocol

## Data Availability

All data generated or analyzed during this study are included in this published article and the supplementary files. Additional data such as identified participant data can be obtained from the corresponding author upon reasonable request.
