# Supplementary material for: Efficacy and safety of L-oxiracetam on cognitive function in patients with traumatic brain injury: a multicentre, randomised, double-blind, phase 3 clinical trial
Source: Signal Transduct Target Ther. 2025 Dec 12;10:401. doi: 10.1038/s41392-025-02492-5 (PMC12698704; doi:10.1038/s41392-025-02492-5)
Supplement: Supplementary file 1 — Supplemental materials [file 41392_2025_2492_MOESM1_ESM.docx]

Supplementary Materials for

Efficacy and safety of L-oxiracetam on cognitive function in patients with traumatic brain injury: a multicenter, randomized, double-blind, phase 3 clinical trial

Tao Liu^#^; Jiao Wang^#^; Zhihao Zhao^#^; Weiwei Jiang; Minzhi Zhang; Yunhu Yu; Yang Liu; Mingqi Liu; Linan Chen; Hengzhu Zhang; Yingbiao Hong; Bohe Li; Rutong Yu; Hongming Ji; Liang Mi; Biao Zhao; Chuanxiang Lv; Chenglong Liu; Jianning Zhang*; Rongcai Jiang*; for the LOCATE Trial Investigators

Correspondence to: jiang116216@163.com & jianningzhang@hotmail.com

**This PDF file includes:**

Appendix 1-4

Supplementary Tables 1 to 18

Appendix 1. List of LOCATE study group and trial investigators

**1.1 Trial Steering Committee**

Professor Jianning Zhang (Chair), Tianjin Medical University General hospital, China

Professor Rongcai Jiang (Principal Investigator), Tianjin Medical University General hospital, China

Dr Tao Liu (Member), Tianjin Medical University General hospital & The George Institute for Global Health, Australia

Dr Zhihao Zhao, Tianjin Medical University General hospital, China

Dr Weiwei Jiang, Tianjin Medical University General hospital, China

Dr Ye Tian (observer), Tianjin Medical University General hospital, China

Dr Xiaopeng Yan (observer), Shanxi Provincial People's Hospital, China

Ms Fuhe Hu (patient representative), China

Ms Juexin Zhou (patient representative), China

Dr Liusong Yin, (member, independent), Sunho (China) Biopharmaceutical Co.,Ltd.

Dr Dongcheng Jiang, (member, independent), Sunho (China) Biopharmaceutical Co.,Ltd.

**1.2 Statisticians**

Professor Jiao Wang (Member), College of Preventive Medicine, Third Military Medical University, China

Yuanping Yue (Statistician), Department of Biostatistics, School of Public Health, Nanjing Medical University, China

**1.3 Principal Investigators and Coordinators (center, with numbers of patients in**

**parentheses)**

*Subei People's Hospital of Jiangsu province (62):* Hengzhu Zhang, Professor; *Jieyang People's Hospital (55):* Yingbiao Hong, Professor; *Yichun People's Hospital(45):* Bohe Li, Professor; *The Affiliated Hospital of Xuzhou Medical University (40)*: Rutong Yu, Professor; *Shanxi Provincial People's Hospital (33):* Hongming Ji, Professor; *Yan'an Hospital of Kunming City (29):* Taikun Tang, Professor; *Dazhu County People's Hospital (26):* Sheng Zhu, Professor; *The People's Hospital of Yuechi County (24):* Hangang Jiang, Professor; *Yan'an University Xianyang Hospital (20):* Zengqiang Liu, Professor; *Pu'er City People's Hospital (20):* Xingchang Li, Professor; *The First Affiliated Hospital of Anhui Medical University North District(17)*: Tao Jiang, Professor; *Panzhihua Central Hospital(17):* Jianbo Wei, Professor; *The First People's Hospital of Nanning (15):* Feng Wei, Professor; *The Affiliated Huaian NO.1 People's Hospital of Nanjing Medical University (14):* Lianshu Ding, Professor; *Yangquan Coal Industry (group) General Hospital (14):* Weilong Li, Professor; *The First People's Hospital of Jinzhong(14):* Shutian Wei, Professor; *Zhumadian Central Hospital (13):*Li Li, Professor; *The First People's Hospital of Lianyungang (12):* Aiming Li, Professor; *The Second Affiliated Hospital of Nanchang University (11):* Xingen Zhu, Professor; *The First People's Hospital of Changzhou (9):* Ya Peng, Professor; *The First People's Hospital of Shenyang (8):* Hui Li, Professor; *Inner* *Mongolia Autonomous Region People's Hospital (7):* Ruijian Zhang, Professor; *Zhangzhou Municipal Hospital of Fujian Province (7):* Ruisheng Lin, Professor; *Haikou People's Hospital (7):* Ying Xia, Professor; *Liuzhou Worker's Hospital (7):* Chaoyong Wen, Professor; *Taizhou First People's Hospital (7):* Jian Wu, Professor; *Ganzhou People's Hospital (6):* Qiuhua Jiang, Professor; *Beijing Chaoyang Hospital, Capital Medical University (5):* Jinping Li, Professor; *First Affiliated Hospital of Gannan Medical University (5):*Shaochun Yang, Professor; *Hainan Third People's Hospital (5):* Gang Li, Professor; *Suzhou Kowloon Hospital (4):*Zhimin Wang, Professor; *Affiliated Hospital of Nantong University (4):* Jian Chen, Professor; *Affiliated Hospital of Jiangsu University (4):* Zejun Chen, Professor; *The First People's Hospital of Changde City (4):* Tao Mei, Professor; *Linfen People's Hospital (4):* Jianrong Liang, Professor; *Guilin People's Hospital (4):* Bin Li, Professor; *The First Affiliated Hospital of Ningbo University (3):* Xiang Gao, Professor; *The First Affiliated Hospital of Henan University of Science and Technology (2):* Bo Fan, Professor; *The Second Affiliated Hospital of Guangzhou Medical University (2):* Yezhong Wang, Professor; *Huizhou Central People's Hospital(2):* Gang Zhu, Professor; *Zhejiang Provincial People's Hospital(2):* Lin Lou Professor; *The First Affiliated Hospital of Nanchang University (1):* Meihua Li, Professor; *The Second People Hospital (1):* Guoqing Jian, Professor.

Appendix 2. Study timeline

| **Major Study Milestones** | **2019** | **2020** | **2021** | **2022** | **2023** | **2024** | **2025** |
| --- | --- | --- | --- | --- | --- | --- | --- |
|  | **9-12 months** |  |  |  |  |  |  |
| Site Selection |  |  |  |  |  |  |  |
| Ethics Application |  |  |  |  |  |  |  |
| Site Training |  |  |  |  |  |  |  |
| Recruitment and Intervention |  |  |  |  |  |  |  |
| Endpoint Evaluation |  |  |  |  |  |  |  |
| Data Cleaning and Closure |  |  |  |  |  |  |  |
| Analysis and Results |  |  |  |  |  |  |  |
| Presentation and Publication |  |  |  |  |  |  |  |

# Appendix 3. CONSORT 2010 checklist of information to include when reporting a randomised trial^*^

| **Section/Topic** | **Item No** | **Checklist Item** | **Reported on page No** |
| --- | --- | --- | --- |
| **Title and abstract** | | | |
|  | 1a | Identification as a randomized trial in the title | 1 |
|  | 1b | Structured summary of trial design, methods, results, and conclusions (for specific guidance see CONSORT for abstracts) | 3 |
| **Introduction** | | | |
| Background and objectives | 2a | Scientific background and explanation of rationale | 4 |
|  | 2b | Specific objectives or hypotheses | 4 |
| **Methods** | | | |
| Trial design | 3a | Description of trial design (such as parallel, factorial) including allocation ratio | 11 |
|  | 3b | Important changes to methods after trial commencement (such as eligibility criteria), with reasons | 11 |
| Participants | 4a | Eligibility criteria for participants | 11 |
|  | 4b | Settings and locations where the data were collected | 11 |
| Interventions | 5 | The interventions for each group with sufficient details to allow replication, including how and when they were actually administered | 12,13 |
| Outcomes | 6a | Completely defined pre-specified primary and secondary outcome measures, including how and when they were assessed | 13 |
|  | 6b | Any changes to trial outcomes after the trial commenced, with reasons | 13 |
| Sample size | 7a | How sample size was determined | 13,14 |
|  | 7b | When applicable, explanation of any interim analyses and stopping guidelines | 14,15 |
| **Randomization** |  |  |  |
| Sequence generation | 8a | Method used to generate the random allocation sequence | 12 |
|  | 8b | Type of randomization; details of any restriction (such as blocking and block size) | 12 |
| Allocation concealment  mechanism | 9 | Mechanism used to implement the random allocation sequence (such as sequentially numbered containers), describing any steps taken to conceal the sequence until interventions were assigned | 12 |
| Implementation | 10 | Who generated the random allocation sequence, who enrolled participants, and who assigned participants to interventions | 12 |
| **Blinding** | 11a | If done, who was blinded after assignment to interventions (for example, participants, care providers, those assessing outcomes) and how | 12 |
|  | 11b | If relevant, description of the similarity of interventions |  |
| Statistical methods | 12a | Statistical methods used to compare groups for primary and secondary outcomes | 13-15 |
|  | 12b | Methods for additional analyses, such as subgroup analyses and adjusted analyses | 15 |
| **Results** | | | |
| Participant flow (a diagram is strongly recommended) | 13a | For each group, the numbers of participants who were randomly assigned, received intended treatment, and were analysed for the primary outcome | 5 |
|  | 13b | For each group, losses and exclusions after randomisation, together with reasons | 5 |
| Recruitment | 14a | Dates defining the periods of recruitment and follow-up | 5 |
|  | 14b | Why the trial ended or was stopped | 5 |
| Baseline data | 15 | A table showing baseline demographic and clinical characteristics for each group | 5 |
| Numbers analysed | 16 | For each group, number of participants (denominator) included in each analysis and whether the analysis was by original assigned groups | 5 |
| Outcomes and estimation | 17a | For each primary and secondary outcome, results for each group, and the estimated effect size and its precision (such as 95% confidence interval) | 5,6 |
|  | 17b | For binary outcomes, presentation of both absolute and relative effect sizes is recommended | 5,6 |
| Ancillary analyses | 18 | Results of any other analyses performed, including subgroup analyses and adjusted analyses, distinguishing pre-specified from exploratory | 6,7 |
| Harms | 19 | All important harms or unintended effects in each group (for specific guidance see CONSORT for harms) | 7 |
| **Discussion** | | | |
| Limitations | 20 | Trial limitations, addressing sources of potential bias, imprecision, and, if relevant, multiplicity of analyses | 9,10 |
| Generalisability | 21 | Generalisability (external validity, applicability) of the trial findings | 8,10 |
| Interpretation | 22 | Interpretation consistent with results, balancing benefits and harms, and considering other relevant evidence | 8-10 |
| **Other information** | | |  |
| Registration | 23 | Registration number and name of trial registry | 3 |
| Protocol | 24 | Where the full trial protocol can be accessed, if available | 16 |
| Funding | 25 | Sources of funding and other support (such as supply of drugs), role of funders | 16,17 |

*We strongly recommend reading this statement in conjunction with the CONSORT 2010 Explanation and Elaboration for important clarifications on all the items. If relevant, we also recommend reading CONSORT extensions for cluster randomised trials, non-inferiority and equivalence trials, non-pharmacological treatments, herbal interventions, and pragmatic trials. Additional extensions are forthcoming: for those and for up-to-date references relevant to this checklist, see www.consort-statement.org

**Appendix 4. Nonauthor Collaborators**

| **First Name** | **Last Name** | **Degree** | **Institution** | **Location (city, province, country)** | **Role or Contribution** |
| --- | --- | --- | --- | --- | --- |
| Taikun | Tang | MD | Yan'an Hospital of Kunming City | Kunming, Yunnan, China | Principal investigator |
| Sheng | Zhu | MD | Dazhu County People's Hospital | Dazhu, Sichuan, China | Principal investigator |
| Hangang | Jiang | MD | The People's Hospital of Yuechi County | Yuechi, Sichuan, China | Principal investigator |
| Zengqiang | Liu | MD | Yan'an University Xianyang Hospital | Xianyang, Shaanxi, China | Principal investigator |
| Xingchang | Li | MD | Pu'er City People's Hospital | Pu'er, Yunnan, China | Principal investigator |
| Jianbo | Wei | MD | Panzhihua Central Hospital | Panzhihua, Sichuan, China | Principal investigator |
| Tao | Jiang | MD | First Affiliated Hospital of Anhui Medical University North District | Hefei, Anhui, China | Principal investigator |
| Feng | Wei | MD | The First People's Hospital of Nanning | Nanning, Guangxi, China | Principal investigator |
| Lianshu | Ding | MD | Huaian NO.1 People's Hospital of Nanjing Medical University | Huai'an, Jiangsu, China | Principal investigator |
| Weilong | Li | MD | Yangquan Coal Industry General Hospital | Yangquan, Shanxi, China | Principal investigator |
| Shutian | Wei | MD | The First People's Hospital of Jinzhong | Jinzhong, Shanxi, China | Principal investigator |
| Li | Li | MD | Zhumadian Central Hospital | Zhumadian, Henan, China | Principal investigator |
| Aiming | Li | MD | The First People's Hospital of Lianyungang | Lianyungang, Jiangsu, China | Principal investigator |
| Xingen | Zhu | MD | The Second Affiliated Hospital of Nanchang University | Nanchang, Jiangxi, China | Principal investigator |
| Ya | Peng | MD | The First People's Hospital of Changzhou | Changzhou, Jiangsu, China | Principal investigator |
| Hui | Li | MD | The First People's Hospital of Shenyang | Shenyang, Liaoning, China | Principal investigator |
| Ruijian | Zhang | MD | Inner Mongolia Autonomous Region People's Hospital | Hohhot, Inner Mongolia, China | Principal investigator |
| Ruisheng | Lin | MD | Zhangzhou Municipal Hospital of Fujian Province | Zhangzhou, Fujian, China | Principal investigator |
| Ying | Xia | MD | Haikou People's Hospital | Haikou, Hainan, China | Principal investigator |
| Chaoyong | Wen | MD | Liuzhou Worker's Hospital | Liuzhou, Guangxi, China | Principal investigator |
| Jian | Wu | MD | Taizhou First People's Hospital – | Taizhou, Zhejiang, China | Principal investigator |
| Qiuhua | Jiang | MD | Ganzhou People's Hospital | Ganzhou, Jiangxi, China | Principal investigator |
| Jinping | Li | MD | Beijing Chaoyang Hospital | Beijing, China | Principal investigator |
| Shaochun | Yang | MD | First Affiliated Hospital of Gannan Medical University | Ganzhou, Jiangxi, China | Principal investigator |
| Gang | Li | MD | Hainan Third People's Hospital | Sanya, Hainan, China | Principal investigator |
| Zhimin | Wang | MD | Suzhou Kowloon Hospital | Suzhou, Jiangsu, China | Principal investigator |
| Jian | Chen | MD | Affiliated Hospital of Nantong University | Nantong, Jiangsu, China | Principal investigator |
| Zejun | Chen | MD | Affiliated Hospital of Jiangsu University | Zhenjiang, Jiangsu, China | Principal investigator |
| Tao | Mei | MD | The First People's Hospital of Changde City | Changde, Hunan, China | Principal investigator |
| Jianrong | Liang | MD | Linfen People's Hospital | Linfen, Shanxi, China | Principal investigator |
| Bin | Li | MD | Guilin People's Hospital | Guilin, Guangxi, China | Principal investigator |
| Xiang | Gao | MD | First Affiliated Hospital of Ningbo University | Ningbo, Zhejiang, China | Principal investigator |
| Bo | Fan | MD | Affiliated Hospital of Henan University of Science and Technology | Luoyang, Henan, China | Principal investigator |
| Yezhong | Wang | MD | The Second Affiliated Hospital of Guangzhou Medical University | Guangzhou, Guangdong, China | Principal investigator |
| Gang | Zhu | MD | Huizhou Central People's Hospital | Huizhou, Guangdong, China | Principal investigator |
| Lin | Lou | MD | Zhejiang Provincial People's Hospital | Hangzhou, Zhejiang, China | Principal investigator |
| Meihua | Li | MD | The First Affiliated Hospital of Nanchang University | Nanchang, Jiangxi, China | Principal investigator |
| Guoqing | Jian | MD | The Second Hospital of Tianjin Medical University | Tianjin, China | Principal investigator |

# Supplementary Table 1. Demographic and baseline characteristics of the per-protocol population.

| **Characteristics** | **L-oxiracetam**  **(n=186)** | **Oxiracetam**  **(n=174)** | **Placebo**  **(n=84)** |
| --- | --- | --- | --- |
| **Age, mean (SD), yr** | 50.2 (14.15) | 50.1 (14.60) | 51.0 (13.40) |
| <65, n (%) | 151 (81.18) | 140 (80.46) | 73 (86.90) |
| ≥65, n (%) | 35 (18.82) | 34 (19.54) | 11 (13.10) |
| **Sex, n (%)** |  |  |  |
| Male | 130 (69.89) | 128 (73.56) | 61 (72.62) |
| Female | 56 (30.11) | 46 (26.44) | 23 (27.38) |
| **Race, n (%)** |  |  |  |
| Han nationality | 176 (94.62) | 165 (94.83) | 78 (92.86) |
| Minorities | 10 (5.38) | 9 (5.17) | 6 (7.14) |
| **Education level, n (%)** |  |  |  |
| None | 13 (6.99) | 14 (8.05) | 9 (10.71) |
| Primary school | 56 (30.11) | 57 (32.76) | 30 (35.71) |
| Secondary school or Higher | 117 (62.90) | 103 (59.20) | 45 (53.57) |
| **Drug allergy history, n (%)** | 13 (6.99) | 7 (4.02) | 2 (2.38) |
| **Body temperature, mean (SD)** | 36.6 (0.28) | 36.6 (0.36) | 36.6 (0.29) |
| **Respiratory rate, mean (SD)** | 18.9 (1.60) | 18.8 (1.52) | 18.8 (1.79) |
| **Heart rate, mean (SD)** | 76.1 (11.86) | 75.8 (10.46) | 76.0 (13.02) |
| **Blood pressure, mean (SD)** |  |  |  |
| Systolic | 125.6 (15.01) | 124.9 (14.88) | 122.2 (14.94) |
| Diastolic | 76.2 (10.08) | 75.0 (9.99) | 76.0 (9.67) |
| **QT interval^†^, mean (SD)** | 382.1 (35.34) | 389.6 (35.95) | 389.3 (33.04) |
| **Cause of injury, n (%)** |  |  |  |
| Road-traffic incident | 73 (39.25) | 67 (38.51) | 31 (36.90) |
| Incidental fall |  |  |  |
| Ground-level fall | 30 (16.13) | 43 (24.71) | 15 (17.86) |
| Fall from height | 55 (29.57) | 40 (22.99) | 26 (30.95) |
| **Mechanism of injury, n (%)** |  |  |  |
| Primary injury | 154 (82.80) | 130 (74.71) | 65 (77.38) |
| Secondary injury | 6 (3.23) | 6 (3.45) | 4 (4.76) |
| Unknown | 26 (13.98) | 38 (21.84) | 15 (17.86) |
| **TBI severity, n (%)** |  |  |  |
| Mild | 174 (93.55) | 166 (95.40) | 79 (94.05) |
| Moderate | 12 (6.45) | 8 (4.60) | 5 (5.95) |
| **Non-surgical treatment, n (%)** | 182 (97.85) | 172 (98.85) | 83 (98.81) |
| **Testing scores at baseline** |  |  |  |
| LOTCA, mean (SD) | 72.9 (21.55) | 74.5 (21.11) | 74.8 (20.52) |
| MoCA, mean (SD) | 14.6 (6.12) | 15.7 (6.29) | 14.9 (6.04) |
| MMSE, mean (SD) | 19.5 (5.68) | 19.7 (5.49) | 19.3 (4.96) |
| GCS, median (IQR) | 15.0 (14.00~15.00) | 15.0 (14.00~15.00) | 15.0 (14.00~15.00) |

**^†^**QT interval, the length of the QT interval is related to the heart rate. Abnormalities in the QT interval may be associated with cardiac diseases, especially those that can lead to arrhythmias

Abbreviations: TBI, Traumatic Brain Injury; LOTCA, Loewenstein Occupational Therapy Cognitive Assessment; MoCA, Montreal Cognitive Assessment; MMSE, Mini-Mental State Examination; GCS, Glasgow Coma Scale.

# Supplementary Table 2. Demographic and baseline characteristics of the per-protocol population at each study site.

| **Site** | **Baseline LOTCA** | **90 days LOTCA** | **Age** | **Sex** | | **Education** | | | **TBI severity** | |
| --- | --- | --- | --- | --- | --- | --- | --- | --- | --- | --- |
|  |  |  |  | **Female** | **Male** | **None** | **Primary school** | **Secondary school**  **or Higher** | **Mild TBI** | **Moderate TBI** |
| Site 2 | 105.8 (13.68) | 118.0 (1.22) | 45.6 (12.50) | 1 (20.00) | 4 (80.00) | 0 (0.00) | 0 (0.00) | 5 (100.00) | 5 (100.00) | 0 (0.00) |
| Site 3 | 65.4 (23.44) | 90.9 (25.47) | 45.0 (16.61) | 0 (0.00) | 7 (100.00) | 0 (0.00) | 3 (42.86) | 4 (57.14) | 6 (85.71) | 1 (14.29) |
| Site 5 | 65.6 (27.48) | 79.1 (27.99) | 57.5 (8.59) | 2 (25.00) | 6 (75.00) | 0 (0.00) | 3 (37.50) | 5 (62.50) | 8 (100.00) | 0 (0.00) |
| Site 9 | 73.5 (18.69) | 98.2 (13.49) | 49.0 (12.41) | 6 (18.18) | 27 (81.82) | 2 (6.06) | 10 (30.30) | 21 (63.64) | 32 (96.97) | 1 (3.03) |
| Site 11 | 106.5 (3.54) | 117.5 (0.71) | 35.5 (2.12) | 0 (0.00) | 2 (100.00) | 0 (0.00) | 0 (0.00) | 2 (100.00) | 2 (100.00) | 0 (0.00) |
| Site 12 | 82.0 (13.08) | 104.3 (6.43) | 54.3 (5.51) | 1 (33.33) | 2 (66.67) | 0 (0.00) | 0 (0.00) | 3 (100.00) | 3 (100.00) | 0 (0.00) |
| Site 13 | 61.3 (16.53) | 84.5 (21.16) | 51.4 (14.16) | 17 (42.50) | 23 (57.50) | 2 (5.00) | 13 (32.50) | 25 (62.50) | 40 (100.00) | 0 (0.00) |
| Site 14 | 85.7 (15.72) | 102.3 (13.91) | 49.0 (12.08) | 3 (25.00) | 9 (75.00) | 1 (8.33) | 6 (50.00) | 5 (41.67) | 12 (100.00) | 0 (0.00) |
| Site 15 | 89.3 (12.61) | 95.8 (23.56) | 42.3 (16.32) | 1 (25.00) | 3 (75.00) | 0 (0.00) | 2 (50.00) | 2 (50.00) | 4 (100.00) | 0 (0.00) |
| Site 16 | 73.6 (13.81) | 91.9 (24.35) | 56.4 (12.06) | 6 (42.86) | 8 (57.14) | 2 (14.29) | 5 (35.71) | 7 (50.00) | 14 (100.00) | 0 (0.00) |
| Site 18 | 53.5 (20.72) | 76.4 (26.35) | 54.0 (13.81) | 17 (27.42) | 45 (72.58) | 9 (14.52) | 20 (32.26) | 33 (53.23) | 49 (79.03) | 13 (20.97) |
| Site 19 | 80.5 (13.99) | 98.5 (13.00) | 56.0 (16.57) | 0 (0.00) | 4 (100.00) | 0 (0.00) | 1 (25.00) | 3 (75.00) | 3 (75.00) | 1 (25.00) |
| Site 20 | 70.8 (15.90) | 81.0 (25.55) | 49.8 (11.64) | 2 (50.00) | 2 (50.00) | 2 (50.00) | 1 (25.00) | 1 (25.00) | 4 (100.00) | 0 (0.00) |
| Site 21 | 66.9 (27.30) | 91.2 (23.16) | 63.2 (6.55) | 2 (22.22) | 7 (77.78) | 0 (0.00) | 2 (22.22) | 7 (77.78) | 8 (88.89) | 1 (11.11) |
| Site 23 | 63.6 (31.33) | 91.1 (32.59) | 48.6 (16.63) | 1 (14.29) | 6 (85.71) | 0 (0.00) | 0 (0.00) | 7 (100.00) | 7 (100.00) | 0 (0.00) |
| Site 25 | 54.0 (0.00) | 54.0 (0.00) | 50.0 (7.07) | 0 (0.00) | 2 (100.00) | 0 (0.00) | 1 (50.00) | 1 (50.00) | 2 (100.00) | 0 (0.00) |
| Site 27 | 77.0 (4.24) | 91.0 (15.56) | 62.0 (15.56) | 1 (50.00) | 1 (50.00) | 0 (0.00) | 1 (50.00) | 1 (50.00) | 2 (100.00) | 0 (0.00) |
| Site 28 | 71.6 (21.83) | 90.8 (18.20) | 47.8 (15.67) | 16 (35.56) | 29 (64.44) | 2 (4.44) | 17 (37.78) | 26 (57.78) | 45 (100.00) | 0 (0.00) |
| Site 29 | 73.5 (27.94) | 100.9 (19.71) | 45.4 (12.27) | 4 (36.36) | 7 (63.64) | 1 (9.09) | 3 (27.27) | 7 (63.64) | 9 (81.82) | 2 (18.18) |
| Site 30 | 67.6 (26.60) | 83.8 (33.24) | 52.0 (22.62) | 1 (20.00) | 4 (80.00) | 2 (40.00) | 1 (20.00) | 2 (40.00) | 5 (100.00) | 0 (0.00) |
| Site 31 | 101.0 (22.09) | 111.4 (6.50) | 37.4 (15.33) | 0 (0.00) | 7 (100.00) | 0 (0.00) | 0 (0.00) | 7 (100.00) | 7 (100.00) | 0 (0.00) |
| Site 33 | 83.1 (13.98) | 100.7 (12.59) | 47.5 (12.19) | 1 (6.67) | 14 (93.33) | 0 (0.00) | 4 (26.67) | 11 (73.33) | 15 (100.00) | 0 (0.00) |
| Site 35 | 90.3 (12.38) | 106.4 (11.07) | 46.4 (17.15) | 2 (28.57) | 5 (71.43) | 0 (0.00) | 1 (14.29) | 6 (85.71) | 7 (100.00) | 0 (0.00) |
| Site 37 | 74.0 (15.99) | 93.4 (16.99) | 48.2 (16.78) | 7 (24.14) | 22 (75.86) | 4 (13.79) | 12 (41.38) | 13 (44.83) | 29 (100.00) | 0 (0.00) |
| Site 39 | 70.2 (16.66) | 85.3 (15.68) | 57.2 (13.67) | 8 (33.33) | 16 (66.67) | 4 (16.67) | 12 (50.00) | 8 (33.33) | 20 (83.33) | 4 (16.67) |
| Site 41 | 80.9 (19.00) | 97.5 (14.33) | 49.4 (16.64) | 16 (29.63) | 38 (70.37) | 3 (5.56) | 16 (29.63) | 35 (64.81) | 53 (98.15) | 1 (1.85) |
| Site 42 | 71.3 (15.83) | 94.2 (14.24) | 54.8 (12.13) | 7 (35.00) | 13 (65.00) | 1 (5.00) | 5 (25.00) | 14 (70.00) | 20 (100.00) | 0 (0.00) |
| Site 43 | 82.0 (23.13) | 103.0 (16.94) | 41.7 (12.03) | 5 (25.00) | 15 (75.00) | 4 (20.00) | 4 (20.00) | 12 (60.00) | 19 (95.00) | 1 (5.00) |
| Site 44 | 59.0 (.) | 83.0 (.) | 70.0 (.) | 0 (0.00) | 1 (100.00) | 1 (100.00) | 0 (0.00) | 0 (0.00) | 1 (100.00) | 0 (0.00) |
| Site 45 | 84.6 (18.38) | 112.9 (5.05) | 53.0 (8.64) | 0 (0.00) | 14 (100.00) | 0 (0.00) | 5 (35.71) | 9 (64.29) | 14 (100.00) | 0 (0.00) |
| Site 46 | 94.5 (7.78) | 106.5 (3.54) | 52.5 (0.71) | 1 (50.00) | 1 (50.00) | 0 (0.00) | 1 (50.00) | 1 (50.00) | 2 (100.00) | 0 (0.00) |
| Site 47 | 72.9 (30.69) | 86.1 (33.69) | 44.4 (11.36) | 3 (42.86) | 4 (57.14) | 0 (0.00) | 4 (57.14) | 3 (42.86) | 6 (85.71) | 1 (14.29) |
| Site 49 | 60.2 (10.96) | 76.0 (15.23) | 62.2 (9.93) | 11 (42.31) | 15 (57.69) | 7 (26.92) | 12 (46.15) | 7 (26.92) | 26 (100.00) | 0 (0.00) |
| Site 54 | 84.8 (21.59) | 101.6 (11.69) | 56.9 (14.14) | 3 (23.08) | 10 (76.92) | 1 (7.69) | 2 (15.38) | 10 (76.92) | 13 (100.00) | 0 (0.00) |
| Site 58 | 106.4 (10.57) | 114.8 (4.02) | 28.0 (5.15) | 1 (20.00) | 4 (80.00) | 0 (0.00) | 1 (20.00) | 4 (80.00) | 5 (100.00) | 0 (0.00) |
| Site 61 | 49.0 (18.18) | 94.3 (23.18) | 52.3 (14.91) | 3 (50.00) | 3 (50.00) | 0 (0.00) | 4 (66.67) | 2 (33.33) | 4 (66.67) | 2 (33.33) |
| Site 62 | 52.3 (6.70) | 94.0 (12.94) | 60.0 (9.27) | 2 (50.00) | 2 (50.00) | 0 (0.00) | 2 (50.00) | 2 (50.00) | 4 (100.00) | 0 (0.00) |
| Site 63 | 76.5 (27.20) | 99.8 (8.81) | 61.3 (6.75) | 2 (50.00) | 2 (50.00) | 0 (0.00) | 1 (25.00) | 3 (75.00) | 3 (75.00) | 1 (25.00) |
| Site 66 | 72.3 (24.59) | 102.5 (15.15) | 52.3 (10.84) | 6 (42.86) | 8 (57.14) | 3 (21.43) | 2 (14.29) | 9 (64.29) | 13 (92.86) | 1 (7.14) |
| Site 68 | 42.0 (.) | 119.0 (.) | 53.0 (.) | 0 (0.00) | 1 (100.00) | 0 (0.00) | 0 (0.00) | 1 (100.00) | 0 (0.00) | 1 (100.00) |
| Site 71 | 65.1 (21.13) | 84.9 (23.24) | 46.3 (15.13) | 6 (35.29) | 11 (64.71) | 2 (11.76) | 6 (35.29) | 9 (52.94) | 15 (88.24) | 2 (11.76) |
| Site 73 | 88.5 (15.80) | 111.8 (14.50) | 44.5 (13.77) | 1 (25.00) | 3 (75.00) | 0 (0.00) | 1 (25.00) | 3 (75.00) | 4 (100.00) | 0 (0.00) |
| Site 74 | 73.8 (16.13) | 106.1 (14.63) | 47.6 (13.81) | 3 (17.65) | 14 (82.35) | 2 (11.76) | 2 (11.76) | 13 (76.47) | 14 (82.35) | 3 (17.65) |

Abbreviations: LOTCA, Loewenstein Occupational Therapy Cognitive Assessment; TBI, Traumatic Brain Injury.

# Supplementary Table 3. Baseline characteristics of participants complete and not complete follow-up visits.

| **Characteristics** | **Loss of follow-up**  **(n=119)** | **Complete follow-up visit**  **(n=472)** | **P** |
| --- | --- | --- | --- |
| **Age, mean (SD), yr** | 51.9 ± 16.08 | 50.6 ± 14.00 | 0.3995 |
| <65, n (%) | 85 (71.4) | 387 (82.0) | 0.0102 |
| ≥65, n (%) | 34 (28.6) | 85 (18.0) |  |
| **Sex, n (%)** |  |  | 0.8153 |
| Male | 86 (72.3) | 336 (71.2) |  |
| Female | 33 (27.7) | 136 (28.8) |  |
| **Race, n (%)** |  |  | 0.6112 |
| Han nationality | 111 (93.3) | 446 (94.5) |  |
| Minorities | 8 (6.7) | 26 (5.5) |  |
| **Schooling level completed, n (%)** |  |  | 0.0456 |
| None | 18 (15.1) | 37 (7.8) |  |
| Primary school | 37 (31.1) | 149 (31.6) |  |
| Secondary school or Higher | 64 (53.8) | 286 (60.6) |  |
| **Drug allergy history****,** **n (%)** | 4 (3.4) | 22 (4.7) | 0.5367 |
| **Temperature, mean (SD)** | 36.68 (0.351) | 36.64 (0.315) | 0.2112 |
| **Respiratory rate, mean (SD)** | 18.8 (1.64) | 18.8 (1.61) | 0.8787 |
| **Heart rate, mean (SD)** | 76.4 (10.87) | 75.9 (11.53) | 0.6781 |
| **Blood pressure, mean (SD)** |  |  |  |
| Systolic | 127.3 (14.59) | 124.9 (14.92) | 0.1138 |
| Diastolic | 77.1 (10.98) | 75.6 (9.87) | 0.1644 |
| **QT interval, mean (SD)** | 379.8 (35.80) | 387.2 (35.49) | 0.0451 |
| **Cause of injury, n (%)** |  |  | 0.1540 |
| Road-traffic incident | 47 (39.8) | 186 (39.7) |  |
| Incidental fall |  |  |  |
| Ground-level fall | 28 (23.5) | 92 (19.5) |  |
| Fall from height | 22 (18.5) | 131 (27.8) |  |
| Other |  |  |  |
| **Mechanism of injury, n (%)** |  |  | 0.0099 |
| Primary injury | 79 (66.4) | 372 (78.8) |  |
| Secondary injury | 10 (8.4) | 18 (3.8) |  |
| Unknown | 30 (25.2) | 82 (17.4) |  |
| **TBI severity, n (%)** |  |  | 0.2380 |
| Mild TBI | 109 (91.6) | 446 (94.5) |  |
| Moderate TBI | 10 (8.4) | 26 (5.5) |  |
| **Surgical treatment, n (%)** | 2 (1.7) | 7 (1.5) | 1.0000 |
| **Clinical presentation** |  |  |  |
| LOTCA, mean (SD) | 66.2 (24.07) | 73.4 (21.23) | 0.0014 |
| MoCA, mean (SD) | 13.3 (6.53) | 15.0 (6.18) | 0.0117 |
| MMSE, mean (SD) | 17.9 (5.60) | 19.5 (5.51) | 0.0057 |
| GCS, median (IQR) | 14.3 (1.09) | 14.4 (0.95) | 0.2585 |
| **Intervention** |  |  | 0.5373 |
| L-oxiracetam | 42 (35.3) | 193 (40.9) |  |
| Oxiracetam | 51 (42.9) | 185 (39.2) |  |
| Placebo | 26 (21.8) | 94 (19.9) |  |

Abbreviations: TBI, Traumatic Brain Injury; LOTCA, Loewenstein Occupational Therapy Cognitive Assessment; MoCA, Montreal Cognitive Assessment; MMSE, Mini-Mental State Examination; GCS, Glasgow Coma Scale; IQR, interquartile range.

# Supplementary Table 4. Multi-variable analysis of loss of follow-up.

| **Characteristics** | **Reference** | **OR (95%CI)** | **P** |
| --- | --- | --- | --- |
| Age | <65 vs. ≥65 | 1.386 (0.786,2.444) | 0.2589 |
| Sex | Male vs. Female | 0.851 (0.505,1.436) | 0.5459 |
| Race | Han nationality vs. Minorities | 1.645 (0.683,3.959) | 0.2667 |
| Schooling level completed |  |  |  |
|  | Primary school vs. None | 1.777 (0.825,3.825) | 0.1418 |
|  | Secondary school or Higher vs. None | 1.540 (0.663,3.576) | 0.3153 |
| Drug allergy history | Yes vs. No | 1.123 (0.349,3.617) | 0.8455 |
| Temperature | Continuous | 1.405 (0.720,2.739) | 0.3185 |
| Respiratory rate | Continuous | 0.962 (0.839,1.103) | 0.5795 |
| Heart rate, mean (SD) | Continuous | 0.992 (0.971,1.014) | 0.4988 |
| Systolic blood pressure | Continuous | 1.007 (0.986,1.027) | 0.5301 |
| Diastolic blood pressure | Continuous | 1.007 (0.978,1.036) | 0.6596 |
| QT interval | Continuous | 0.993 (0.987,0.999) | 0.0315 |
| Cause of injury | Road-traffic incident vs. Incidental fall | 1.001 (0.639,1.569) | 0.9957 |
| Mechanism of injury | Primary injury vs. Secondary injury | 2.034 (0.847,4.886) | 0.1121 |
| TBI severity | Mild vs. Moderate | 0.752 (0.190,2.975) | 0.6844 |
| Surgical treatment | Yes vs. No | 0.830 (0.135,5.114) | 0.8413 |
| LOTCA | Continuous | 0.989 (0.972,1.006) | 0.1990 |
| MoCA | Continuous | 1.004 (0.934,1.079) | 0.9203 |
| MMSE | Continuous | 0.979 (0.909,1.054) | 0.5683 |
| GCS | Continuous | 1.197 (0.824,1.737) | 0.3455 |
| Intervention | L-oxiracetam vs. Placebo | 1.241 (0.689,2.238) | 0.4720 |
|  | Oxiracetam vs. Placebo | 1.006 (0.565,1.790) | 0.9842 |

Abbreviations: LOTCA, Loewenstein Occupational Therapy Cognitive Assessment; MoCA, Montreal Cognitive Assessment; MMSE, Mini-Mental State Examination; GCS, Glasgow Coma Scale

# Supplementary Table 5. Additional exploratory analyses of the differences between the Oxiracetam and placebo groups in the intent-to-treat population for the primary and secondary outcomes.

| **Outcome ^a^** | **Oxiracetam** | **Placebo** | **Oxiracetam vs. Placebo** | | |
| --- | --- | --- | --- | --- | --- |
|  |  |  | **Mean Difference**  **(95% CI) ^b^** | **Effect Size**  **(95% CI) ^b^** | **P** |
| **Primary Outcome** |  |  |  |  |  |
| Mean change in LOTCA at 90 days | 15.90 (12.71,19.10) | 11.47 (7.75,15.20) | 4.43 (1.15,7.71) | 0.25 (0.04,0.46) | 0.008 |
| **Secondary Outcomes** |  |  |  |  |  |
| Mean change in LOTCA at 14 days | 11.47 (8.59,14.35) | 7.45 (4.09,10.80) | 4.03 (1.07,6.99) | 0.27 (0.06,0.48) | 0.008 |
| Mean change in MMSE |  |  |  |  |  |
| 14 days | 4.23 (3.43,5.02) | 4.53 (3.59,5.46) | -0.30 (-1.12,0.51) | -0.13 (-0.35,0.09) | 0.467 |
| 90 days | 5.14 (4.22,6.06) | 4.91 (3.87,5.94) | 0.23 (-0.64,1.10) | -0.05 (-0.28,0.19) | 0.600 |
| Mean change in MoCA |  |  |  |  |  |
| 14 days | 3.38 (2.52,4.23) | 3.27 (2.25,4.29) | 0.11 (-0.80,1.01) | -0.01 (-0.23,0.21) | 0.813 |
| 90 days | 4.77 (3.70,5.84) | 4.37 (3.15,5.58) | 0.40 (-0.64,1.44) | -0.00 (-0.24,0.23) | 0.448 |
| Percentage of GOSE 7–8, n (%) |  |  |  |  |  |
| 14 days | 92.21 (87.90,95.40) | 92.72 (86.00,96.80) | -0.42 (-6.47,5.62) | -0.02 (-0.23,0.20) | 0.892 |
| 90 days | 97.31 (93.80,99.10) | 98.92 (94.20,100.00) | -1.64 (-4.76,1.49) | -0.11 (-0.35,0.12) | 0.372 |
| ADL |  |  |  |  |  |
| 14 days | 75.42 (71.21,79.63) | 76.83 (71.81,81.86) | -1.41 (-6.14,3.32) | -0.06 (-0.28,0.16) | 0.558 |
| 30 days | 92.04 (90.06,94.02) | 92.50 (90.13,94.88) | -0.46 (-2.71,1.79) | -0.04 (-0.26,0.18) | 0.687 |
| 60 days | 95.94 (94.59,97.29) | 96.74 (95.12,98.37) | -0.80 (-2.34,0.74) | -0.11 (-0.33,0.11) | 0.607 |
| 90 days | 98.16 (97.04,99.28) | 98.58 (97.31,99.85) | -0.42 (-1.58,0.73) | -0.08 (-0.32,0.16) | 0.471 |
| Change in GCS at 14 days | 0.46 (0.37,0.55) | 0.50 (0.40,0.60) | -0.04 (-0.11,0.04) | -0.08 (-0.30,0.13) | 0.332 |

^a^ The outcome was assessed at 14 days (end of the treatment), 30 days (first follow-up), 60 days (second follow-up), and 90 days (third follow-up) after the end of treatment. The analysis compared the outcomes assessed at corresponding date with baseline.

^b^ Adjusted for age, sex, education, TBI severity, and measurements of each outcome at baseline.

Abbreviations: LOTCA, Loewenstein Occupational Therapy Cognitive Assessment; MMSE, Mini-Mental State Examination; MoCA, Montreal Cognitive Assessment; GOSE, Extended Glasgow Outcome Scale; ADL, activities of daily living; GCS, Glasgow Coma Scale; CI, confidence interval.

# Supplementary Table 6. Group differences of the LOTCA in the intent-to-treat population.

| **Outcome** | **L-oxiracetam** | **Oxiracetam** | **Placebo** | **L-oxiracetam vs. Placebo ^a^** | | | **L-oxiracetam**  **vs. Oxiracetam ^a, b^** |
| --- | --- | --- | --- | --- | --- | --- | --- |
|  |  |  |  | **Mean Difference**  **(95% CI)** | **Effect Size**  **(95% CI)** | **P** | **Mean**  **Difference**  **(95% CI)** |
| **14 days** |  |  |  |  |  |  |  |
| Orientation | 1.70  (1.25,2.15) | 1.91  (1.45,2.36) | 1.54  (1.01,2.06) | 0.17  (-0.30,0.63) | -0.00  (-0.21,0.21) | 0.4868 | -0.20  (-0.59,0.18) |
| Visual perception | 1.67  (1.22,2.11) | 1.21  (0.77,1.66) | 0.90  (0.38,1.42) | 0.77  (0.31,1.23) | 0.23  (0.02,0.44) | 0.0010 | 0.46  (0.08,0.83) |
| Spatial perception | 1.14  (0.77,1.51) | 0.97  (0.59,1.34) | 0.83  (0.40,1.26) | 0.31  (-0.07,0.69) | 0.17  (-0.04,0.38) | 0.1061 | 0.17  (-0.14,0.48) |
| Praxis | 1.24  (0.90,1.59) | 0.99  (0.64,1.34) | 0.79  (0.39,1.19) | 0.45  (0.10,0.81) | 0.21  (0.00,0.42) | 0.0130 | 0.25  (-0.04,0.55) |
| Visuomotor Organization | 2.76  (1.76,3.76) | 1.98  (0.98,2.99) | 0.92  (-0.25,2.09) | 1.84  (0.80,2.88) | 0.33  (0.12,0.54) | 0.0005 | 0.77  (-0.08,1.63) |
| Thinking Operation | 3.43  (2.34,4.52) | 2.77  (1.68,3.86) | 0.84  (-0.43,2.12) | 2.58  (1.45,3.72) | 0.46  (0.25,0.67) | <0.0001 | 0.66  (-0.27,1.59) |
| Attention and Concentration | 0.70  (0.54,0.86) | 0.63  (0.47,0.78) | 0.50  (0.32,0.69) | 0.19  (0.03,0.36) | 0.22  (0.01,0.43) | 0.0218 | 0.07  (-0.06,0.21) |
| **90 days** |  |  |  |  |  |  |  |
| Orientation | 2.38  (1.93,2.82) | 2.10  (1.65,2.55) | 1.94  (1.41,2.46) | 0.44  (-0.02,0.90) | 0.07  (-0.14,0.28) | 0.0599 | 0.28  (-0.10,0.65) |
| Visual perception | 2.15  (1.64,2.65) | 1.63  (1.12,2.13) | 1.17  (0.58,1.77) | 0.97  (0.46,1.49) | 0.26  (0.05,0.47) | 0.0002 | 0.52  (0.10,0.95) |
| Spatial perception | 1.52  (1.12,1.91) | 1.13  (0.74,1.52) | 0.98  (0.53,1.43) | 0.54  (0.14,0.94) | 0.25  (0.04,0.46) | 0.0082 | 0.39  (0.06,0.71) |
| Praxis | 1.59  (1.23,1.95) | 1.20  (0.84,1.56) | 1.04  (0.62,1.46) | 0.55  (0.18,0.92) | 0.22  (0.01,0.43) | 0.0036 | 0.39  (0.09,0.69) |
| Visuomotor Organization | 4.89  (3.84,5.94) | 3.39  (2.35,4.44) | 2.38  (1.16,3.60) | 2.51  (1.42,3.60) | 0.41  (0.20,0.62) | <0.0001 | 1.50  (0.60,2.39) |
| Thinking Operation | 5.53  (4.32,6.75) | 4.24  (3.02,5.45) | 1.87  (0.45,3.28) | 3.67  (2.41,4.93) | 0.58  (0.36,0.79) | <0.0001 | 1.30  (0.26,2.33) |
| Attention and Concentration | 1.09  (0.94,1.25) | 0.96  (0.80,1.11) | 0.78  (0.60,0.96) | 0.31  (0.15,0.47) | 0.31  (0.10,0.52) | 0.0002 | 0.13  (0.00,0.27) |

^a^ Models were adjusted for age, sex, education, TBI severity, and LOTCA at baseline.

^b^ The confidence intervals have not been adjusted for multipicity and cannot be used to infer treatment effects.

Abbreviations: LOTCA, Loewenstein Occupational Therapy Cognitive Assessment; CI, confidence interval.

# Supplementary Table 7. Group differences of the LOTCA in the per-protocol population.

| **Outcome ^a^** | **L-oxiracetam** | **Oxiracetam** | **Placebo** | **L-oxiracetam vs. Placebo ^a^** | | | **L-oxiracetam**  **vs. Oxiracetam ^a,b^** |
| --- | --- | --- | --- | --- | --- | --- | --- |
|  |  |  |  | **Mean Difference**  **(95% CI)** | **Effect Size**  **(95% CI)** | **P** | **Mean**  **Difference**  **(95% CI)** |
| **14 days** |  |  |  |  |  |  |  |
| Orientation | 1.29  (0.79,1.80) | 1.66  (1.14,2.18) | 1.56  (0.97,2.15) | -0.27  (-0.79,0.25) | -0.09  (-0.33,0.15) | 0.3116 | -0.36  (-0.78,0.05) |
| Visual perception | 1.54  (1.07,2.01) | 1.27  (0.79,1.75) | 0.92  (0.37,1.47) | 0.62  (0.14,1.10) | 0.21  (-0.03,0.45) | 0.0115 | 0.27  (-0.12,0.65) |
| Spatial perception | 1.10  (0.71,1.48) | 1.06  (0.67,1.45) | 0.93  (0.50,1.37) | 0.16  (-0.22,0.55) | 0.25  (0.01,0.49) | 0.4010 | 0.04  (-0.27,0.35) |
| Praxis | 1.27  (0.94,1.61) | 1.15  (0.81,1.49) | 1.06  (0.68,1.45) | 0.21  (-0.13,0.55) | 0.22  (-0.02,0.46) | 0.2236 | 0.12  (-0.15,0.39) |
| Visuomotor Organization | 2.22  (1.07,3.36) | 1.91  (0.75,3.08) | 0.93  (-0.40,2.25) | 1.29  (0.12,2.47) | 0.33  (0.09,0.57) | 0.0314 | 0.31  (-0.63,1.25) |
| Thinking Operation | 3.40  (2.11,4.68) | 3.03  (1.72,4.34) | 1.25  (-0.25,2.74) | 2.15  (0.82,3.47) | 0.46  (0.22,0.70) | 0.0015 | 0.36  (-0.70,1.42) |
| Attention and Concentration | 0.67  (0.49,0.84) | 0.66  (0.48,0.84) | 0.62  (0.42,0.82) | 0.05  (-0.14,0.23) | 0.15  (-0.09,0.39) | 0.6158 | 0.00  (-0.14,0.15) |
| **90 days** |  |  |  |  |  |  |  |
| Orientation | 2.24  (1.78,2.70) | 2.06  (1.59,2.53) | 2.27  (1.73,2.80) | -0.03  (-0.50,0.44) | 0.01  (-0.22,0.25) | 0.9069 | 0.18  (-0.20,0.55) |
| Visual perception | 2.20  (1.67,2.73) | 1.88  (1.34,2.43) | 1.44  (0.81,2.06) | 0.77  (0.22,1.31) | 0.22  (-0.02,0.46) | 0.0058 | 0.32  (-0.12,0.75) |
| Spatial perception | 1.58  (1.21,1.94) | 1.36  (1.00,1.73) | 1.27  (0.86,1.69) | 0.30  (-0.06,0.67) | 0.32  (0.08,0.56) | 0.1032 | 0.21  (-0.08,0.50) |
| Praxis | 1.69  (1.39,1.99) | 1.45  (1.14,1.75) | 1.48  (1.14,1.83) | 0.21  (-0.09,0.52) | 0.21  (-0.03,0.45) | 0.1712 | 0.25  (0.00,0.49) |
| Visuomotor Organization | 4.86  (3.75,5.97) | 3.84  (2.71,4.97) | 3.14  (1.85,4.43) | 1.72  (0.58,2.86) | 0.40  (0.16,0.64) | 0.0033 | 1.02  (0.11,1.94) |
| Thinking Operation | 5.97  (4.57,7.36) | 5.08  (3.66,6.50) | 2.68  (1.06,4.31) | 3.28  (1.85,4.72) | 0.62  (0.37,0.86) | <0.0001 | 0.88  (-0.26,2.03) |
| Attention and Concentration | 1.22  (1.08,1.36) | 1.16  (1.01,1.30) | 1.07  (0.91,1.24) | 0.14  (-0.01,0.29) | 0.26  (0.02,0.50) | 0.0587 | 0.06  (-0.06,0.18) |

^a^ Models were adjusted for age, sex, education, TBI severity, and LOTCA at baseline.

^b^ The confidence intervals have not been adjusted for multipicity and cannot be used to infer treatment effects.

Abbreviations: LOTCA, Loewenstein Occupational Therapy Cognitive Assessment; CI, confidence interval.

# Supplementary Table 8 Group differences of the outcomes in per-protocol population (Unadjusted).

| **Outcome ^a^** | **L-oxiracetam** | **Oxiracetam** | **Placebo** | **L-oxiracetam vs. Placebo** | | | **L-oxiracetam vs. Oxiracetam ^d^** |
| --- | --- | --- | --- | --- | --- | --- | --- |
|  |  |  |  | **Mean Difference**  **(95% CI)** | **Effect Size**  **(95% CI)** | **P** | **Mean Difference**  **(95% CI)** |
| **Primary outcome** |  |  |  |  |  |  |  |
| Mean change in LOTCA at 90 days **^b^** | 26.55  (23.93,29.18) | 22.13  (19.41,24.84) | 17.37  (13.46,21.28) | 9.18  (4.47,13.89) | 0.48  (0.23,0.72) | 0.0001 | 4.43  (0.65,8.21) |
| **Secondary outcomes** |  |  |  |  |  |  |  |
| Mean change in LOTCA at 14 days ^c^ | 19.03  (16.79,21.26) | 17.33  (15.03,19.64) | 12.71  (9.39,16.04) | 6.31  (2.31,10.31) | 0.38  (0.14,0.62) | 0.0021 | 1.69  (-1.52,4.90) |
| Mean change in MMSE |  |  |  |  |  |  |  |
| 14 days ^c^ | 5.35  (4.77,5.93) | 5.17  (4.56,5.77) | 5.58  (4.71,6.45) | -0.23  (-1.28,0.81) | -0.06  (-0.29,0.18) | 0.6607 | 0.18  (-0.66,1.02) |
| 90 days ^b^ | 6.85  (6.17,7.54) | 6.09  (5.38,6.80) | 6.27  (5.25.7.30) | 0.58  (-0.65,1.81) | 0.12  (-0.12,0.36) | 0.3540 | 0.77  (-0.22,1.76) |
| Mean change in MoCA |  |  |  |  |  |  |  |
| 14 days ^c^ | 5.47  (4.87,6.08) | 4.34  (3.72,4.97) | 4.15  (3.25,5.06) | 1.32  (0.23,2.41) | 0.31  (0.07,0.55) | 0.0178 | 1.13  (0.25,2.00) |
| 90 days ^b^ | 7.09  (6.35,7.83) | 5.74  (4.98,6.51) | 5.57  (4.47,6.67) | 1.52  (0.19,2.85) | 0.28  (0.04,0.52) | 0.0250 | 1.35  (0.28,2.42) |
| Percentage of GOS-E 7–8, n (%) |  |  |  |  |  |  |  |
| 14 days ^c^ | 88.7  (83.3,92.9) | 92.0  (86.9,95.5) | 94.0  (86.7,98.0) | -5.3  (-12.1,1.5) | -0.18  (-0.42,0.06) | 0.1687 | -3.2  (-9.3,2.8) |
| 90 days ^b^ | 97.3  (93.8,99.1) | 97.7  (94.2,99.4) | 98.8  (93.5,100.0) | -1.5  (-4.8,1.8) | -0.10  (-0.34,0.14) | 0.4396 | -0.4  (-3.6,2.8) |
| ADL |  |  |  |  |  |  |  |
| 14 days ^c^ | 88.95  (86.13,91.77) | 88.85  (85.93,91.77) | 91.07  (86.87,95.27) | -2.12  (-7.18,2.94) | -0.11  (-0.35,0.13) | 0.4104 | 0.10  (-3.96,4.16) |
| 30 days | 97.23  (95.86,98.60) | 97.13  (95.71,98.54) | 97.38  (95.35,99.42) | -0.15  (-2.60,2.30) | -0.02  (-0.25,0.22) | 0.9045 | 0.10  (-1.86,2.07) |
| 60 days | 98.55  (97.65,99.45) | 98.33  (97.40,99.26) | 99.17  (97.83,100.50) | -0.62  (-2.23,0.99) | -0.11  (-0.35,0.13) | 0.4513 | 0.22  (-1.08,1.51) |
| 90 days | 99.30  (98.61,99.99) | 99.05  (98.34,99.77) | 99.35  (98.32,100.37) | -0.04  (-1.28,1.19) | -0.01  (-0.25,0.23) | 0.9441 | 0.25  (-0.74,1.24) |
| Change in GCS at 14 days ^c^ | 0.56  (0.43,0.69) | 0.47  (0.33,0.60) | 0.49  (0.30,0.68) | 0.07  (-0.16,0.30) | 0.08  (-0.16,0.32) | 0.5410 | 0.09  (-0.09,0.28) |

^a^ The outcome was assessed during 14 days (end of the treatment), 30 days (first follow-up period), 60 days (second follow-up period), and 90 days (third follow-up period) after the end of treatment.

^b^ This analysis compared the data at 90 days with baseline.

^c^ This analysis compared the data at the end of treatment with baseline.

^d^ The confidence intervals have not been adjusted for multiplicity and cannot be used to infer treatment effects.

Abbreviations: LOTCA, Loewenstein Occupational Therapy Cognitive Assessment; MMSE, Mini-Mental State Examination; MoCA, Montreal Cognitive Assessment; GOSE, Extended Glasgow Outcome Scale; ADL, activities of daily living; GCS, Glasgow Coma Scale.

# Supplementary Table 9 Group differences of the primary and secondary outcome in per-protocol population (Adjusted).

| **Outcome ^a^** | **L-oxiracetam** **^d^** | **Oxiracetam** **^d^** | **Placebo ^d^** | **L-oxiracetam vs. Placebo** | | **L-oxiracetam vs. Oxiracetam^e^** |
| --- | --- | --- | --- | --- | --- | --- |
|  |  |  |  | **Mean Difference**  **(95% CI) ^d^** | **P** | **Mean Difference**  **(95% CI) ^d^** |
| **Primary outcome** |  |  |  |  |  |  |
| Mean change in LOTCA at 90 days **^b^** | 20.57  (17.24,23.90) | 17.77  (14.40,21.13) | 14.06  (10.25,17.86) | 6.51  (3.18,9.85) | 0.0001 | 2.80  (0.13,5.47) |
| **Secondary outcomes** |  |  |  |  |  |  |
| Mean change in LOTCA at 14 days ^c^ | 12.40  (9.12,15.68) | 11.80  (8.48,15.12) | 8.04  (4.29,11.79) | 4.36  (1.07,7.65) | 0.0096 | 0.60  (-2.03,3.23) |
| Mean change in MMSE |  |  |  |  |  |  |
| 14 days ^c^ | 4.19  (3.26,5.13) | 4.21  (3.26,5.15) | 4.59  (3.53,5.66) | -0.40  (-1.31,0.51) | 0.3867 | -0.01  (-0.74,0.71) |
| 90 days ^b^ | 5.51  (4.60,6.41) | 5.07  (4.15,5.98) | 5.17  (4.13,6.20) | 0.34  (-0.54,1.22) | 0.4494 | 0.44  (-0.27,1.15) |
| Mean change in MoCA |  |  |  |  |  |  |
| 14 days ^c^ | 4.20  (3.18,5.22) | 3.49  (2.48,4.51) | 3.24  (2.08,4.41) | 0.96  (-0.05,1.96) | 0.0617 | 0.71  (-0.10,1.52) |
| 90 days ^b^ | 5.50  (4.40,6.59) | 4.89  (3.80,5.97) | 4.54  (3.29,5.79) | 0.96  (-0.12,2.03) | 0.0816 | 0.61  (-0.25,1.48) |
| Percentage of GOS-E 7–8, n (%) |  |  |  |  |  |  |
| 14 days ^c^ | 88.7  (83.3,92.9) | 92.0  (86.9,95.5) | 94.0  (86.7,98.0) | -5.3  (-12.1,1.5) | 0.1687 | -3.2  (-9.3,2.8) |
| 90 days ^b^ | 97.3  (93.8,99.1) | 97.7  (94.2,99.4) | 98.8  (93.5,100.0) | -1.5  (-4.8,1.8) | 0.4396 | -0.4  (-3.6,2.8) |
| ADL |  |  |  |  |  |  |
| 14 days ^c^ | 79.22  (74.65,83.78) | 79.06  (74.38,83.75) | 81.92  (76.51,87.34) | -2.71  (-7.62,2.21) | 0.2800 | 0.15  (-3.79,4.09) |
| 30 days | 92.59  (90.37,94.81) | 92.44  (90.16,94.72) | 92.96  (90.33,95.60) | -0.37  (-2.76,2.02) | 0.7595 | 0.16  (-1.76,2.07) |
| 60 days | 96.15  (94.68,97.63) | 95.92  (94.41,97.44) | 96.91  (95.16,98.66) | -0.76  (-2.35,0.84) | 0.3510 | 0.23  (-1.04,1.50) |
| 90 days | 98.35  (97.21,99.50) | 98.13  (96.95,99.30) | 98.51  (97.15,99.87) | -0.15  (-1.39,1.08) | 0.8052 | 0.23  (-0.76,1.21) |
| Change in GCS at 14 days ^c^ | 0.42  (0.34,0.50) | 0.40  (0.32,0.49) | 0.41  (0.32,0.50) | 0.01  (-0.06,0.08) | 0.8059 | 0.02  (-0.04,0.07) |

^a^ The outcome was assessed during 14 days (end of the treatment), 30 days (first follow-up period), 60 days (second follow-up period), and 90 days (third follow-up period) after the end of treatment.

^b^ This analysis compared the data at 90 days with baseline.

^c^ This analysis compared the data at the end of treatment with baseline.

^d^ Adjusted for age, sex, education, TBI severity, and measurements of each outcome at baseline.

^e^ The confidence intervals have not been adjusted for multipicity and cannot be used to infer treatment effects.

Abbreviations: LOTCA, Loewenstein Occupational Therapy Cognitive Assessment; MMSE, Mini-Mental State Examination; MoCA, Montreal Cognitive Assessment; GOSE, Extended Glasgow Outcome Scale; ADL, activities of daily living; CI, Confidence Interval; GCS, Glasgow Coma Scale.

# Supplementary Table 10. Group differences of the MMSE in intent-to-treat population.

| **Outcome** | **L-oxiracetam** | **Oxiracetam** | **Placebo** | **L-oxiracetam vs. Placebo ^a^** | | | **L-oxiracetam**  **vs. Oxiracetam ^a, b^** |
| --- | --- | --- | --- | --- | --- | --- | --- |
|  |  |  |  | **Mean Difference**  **(95% CI)** | **Effect Size**  **(95% CI)** | **P** | **Mean Difference**  **(95% CI)** |
| **14 days** |  |  |  |  |  |  |  |
| Orientation | 1.40  (1.10,1.69) | 1.36  (1.07,1.66) | 1.45  (1.10,1.80) | -0.05  (-0.36,0.26) | -0.04  (-0.26,0.17) | 0.7306 | 0.04  (-0.22,0.29) |
| Memory | 0.35  (0.23,0.48) | 0.31  (0.18,0.44) | 0.29  (0.14,0.44) | 0.06  (-0.08,0.20) | 0.11  (-0.10,0.33) | 0.3832 | 0.04  (-0.07,0.16) |
| Attention and calculations | 0.49  (0.19,0.78) | 0.34  (0.05,0.64) | 0.43  (0.07,0.78) | 0.06  (-0.25,0.37) | 0.01  (-0.21,0.22) | 0.7052 | 0.14  (-0.11,0.40) |
| Recall | 0.68  (0.49,0.87) | 0.70  (0.51,0.90) | 0.71  (0.48,0.94) | -0.03  (-0.24,0.18) | -0.07  (-0.28,0.14) | 0.8028 | -0.02  (-0.19,0.15) |
| Language | 0.70  (0.45,0.96) | 0.74  (0.48,0.99) | 0.77  (0.47,1.07) | -0.07  (-0.33,0.19) | -0.06  (-0.27,0.16) | 0.5848 | -0.03  (-0.25,0.18) |
| **90 days** |  |  |  |  |  |  |  |
| Orientation | 1.99  (1.68,2.30) | 1.76  (1.44,2.07) | 1.81  (1.46,2.17) | 0.18  (-0.13,0.48) | 0.10  (-0.13,0.34) | 0.2527 | 0.24  (-0.01,0.48) |
| Memory | 0.47  (0.33,0.60) | 0.42  (0.28,0.55) | 0.39  (0.24,0.54) | 0.08  (-0.06,0.21) | 0.17  (-0.06,0.40) | 0.2713 | 0.05  (-0.06,0.16) |
| Attention and calculations | 0.72  (0.40,1.05) | 0.61  (0.28,0.93) | 0.39  (0.02,0.77) | 0.33  (0.01,0.65) | 0.13  (-0.10,0.36) | 0.0439 | 0.12  (-0.15,0.38) |
| Recall | 0.72  (0.49,0.95) | 0.77  (0.53,1.00) | 0.68  (0.41,0.95) | 0.04  (-0.20,0.28) | 0.02  (-0.21,0.25) | 0.7429 | -0.05  (-0.24,0.15) |
| Language | 1.22  (0.92,1.53) | 1.11  (0.80,1.42) | 1.13  (0.79,1.48) | 0.09  (-0.20,0.38) | 0.02  (-0.21,0.25) | 0.528 | 0.11  (-0.12,0.35) |

^a^ Models were adjusted for age, sex, education, TBI severity, and MMSE at baseline.

^b^ The confidence intervals have not been adjusted for multiplicity and cannot be used to infer treatment effects.

Abbreviations: MMSE, Mini-Mental State Examination; CI, Confidence Interval.

# Supplementary Table 11. Group differences of MoCA in intent-to-treat population.

| **Outcome** | **L-oxiracetam** | **Oxiracetam** | **Placebo** | **L-oxiracetam vs. Placebo ^a^** | | | **L-oxiracetam vs. Oxiracetam ^a,b^** |
| --- | --- | --- | --- | --- | --- | --- | --- |
|  |  |  |  | **Mean Difference**  **(95% CI)** | **Effect Size**  **(95% CI)** | **P** | **Mean**  **Difference**  **(95% CI)** |
| **14 days** |  |  |  |  |  |  |  |
| Executive function | 0.24  (-0.01,0.48) | 0.20  (-0.05,0.44) | 0.10  (-0.19,0.40) | 0.13  (-0.13,0.40) | 0.11  (-0.11,0.32) | 0.3141 | 0.04  (-0.17,0.25) |
| Name | 0.43  (0.28,0.58) | 0.32  (0.17,0.47) | 0.32  (0.14,0.50) | 0.11  (-0.05,0.27) | 0.21  (-0.01,0.42) | 0.1792 | 0.11  (-0.02,0.24) |
| Attention | 0.45  (0.20,0.71) | 0.33  (0.07,0.58) | 0.37  (0.07,0.67) | 0.08  (-0.18,0.35) | 0.07  (-0.15,0.28) | 0.5377 | 0.12  (-0.09,0.34) |
| Language | 0.26  (0.08,0.43) | 0.25  (0.07,0.42) | 0.18  (-0.02,0.39) | 0.07  (-0.11,0.26) | 0.05  (-0.17,0.26) | 0.4458 | 0.01  (-0.14,0.16) |
| Abstract | 0.23  (0.09,0.37) | 0.24  (0.10,0.38) | 0.21  (0.04,0.37) | 0.02  (-0.13,0.17) | 0.05  (-0.17,0.26) | 0.7872 | -0.01  (-0.14,0.11) |
| Delayed memory | 0.73  (0.45,1.02) | 0.58  (0.30,0.87) | 0.43  (0.09,0.77) | 0.30  (-0.01,0.61) | 0.23  (0.02,0.45) | 0.0551 | 0.15  (-0.10,0.40) |
| Orientation | 0.73  (0.52,0.95) | 0.65  (0.43,0.86) | 0.69  (0.43,0.95) | 0.04  (-0.19,0.27) | 0.05  (-0.17,0.26) | 0.7192 | 0.09  (-0.10,0.27) |
| **90 days** |  |  |  |  |  |  |  |
| Executive function | 0.58  (0.29,0.87) | 0.62  (0.32,0.91) | 0.44  (0.10,0.77) | 0.14  (-0.15,0.43) | 0.15  (-0.08,0.39) | 0.3293 | -0.04  (-0.27,0.20) |
| Name | 0.47  (0.30,0.64) | 0.37  (0.19,0.54) | 0.37  (0.18,0.57) | 0.09  (-0.07,0.26) | 0.18  (-0.06,0.41) | 0.2694 | 0.10  (-0.04,0.24) |
| Attention | 0.48  (0.20,0.75) | 0.40  (0.13,0.68) | 0.37  (0.05,0.68) | 0.11  (-0.16,0.38) | 0.09  (-0.15,0.32) | 0.4142 | 0.07  (-0.14,0.29) |
| Language | 0.74  (0.53,0.96) | 0.67  (0.45,0.89) | 0.76  (0.52,1.01) | -0.02  (-0.23,0.19) | -0.06  (-0.30,0.17) | 0.8552 | 0.08  (-0.10,0.25) |
| Abstract | 0.36  (0.19,0.54) | 0.26  (0.08,0.43) | 0.28  (0.08,0.48) | 0.08  (-0.09,0.26) | 0.12  (-0.12,0.35) | 0.3572 | 0.11  (-0.04,0.25) |
| Delayed memory | 1.07  (0.70,1.45) | 0.99  (0.61,1.37) | 0.57  (0.15,1.00) | 0.50  (0.12,0.88) | 0.36  (0.13,0.59) | 0.0093 | 0.08  (-0.23,0.39) |
| Orientation | 0.96  (0.75,1.18) | 0.82  (0.61,1.04) | 0.82  (0.57,1.06) | 0.15  (-0.07,0.36) | 0.14  (-0.09,0.38) | 0.1780 | 0.14  (-0.04,0.32) |

^a^ Models were adjusted for age, sex, education, TBI severity, and MoCA at baseline.

^b^The confidence intervals have not been adjusted for multipicity and cannot be used to infer treatment effects.

Abbreviations: MoCA, Montreal Cognitive Assessment: CI, Confidence Interval.

# Supplementary Table 12. Group differences of GCS in intent-to-treat population.

| **Outcome** | **L-oxiracetam** | **Oxiracetam** | **Placebo** | **L-oxiracetam vs. Placebo ^a^** | | | **L-oxiracetam vs. Oxiracetam ^a,b^** |
| --- | --- | --- | --- | --- | --- | --- | --- |
|  |  |  |  | **Proportion Difference**  **(95% CI)** | **Effect Size**  **(95% CI)** | **P** | **Proportion Difference**  **(95% CI)** |
| **14 days** |  |  |  |  |  |  |  |
| Eye-opening reaction | 0.23  (0.21,0.25) | 0.24  (0.22,0.26) | 0.23  (0.21,0.25) | -0.00  (-0.02,0.02) | -0.07  (-0.28,0.15) | 0.9686 | -0.01  (-0.02,0.01) |
| Verbal responses | 0.20  (0.14,0.26) | 0.17  (0.11,0.23) | 0.20  (0.13,0.27) | 0.00  (-0.06,0.06) | -0.00  (-0.22,0.21) | 0.9641 | 0.03  (-0.02,0.08) |
| Motor responses | 0.09  (0.07,0.11) | 0.08  (0.06,0.10) | 0.09  (0.07,0.12) | -0.01  (-0.02,0.01) | -0.03  (-0.24,0.19) | 0.5476 | 0.01  (-0.01,0.02) |

^a^ Models were adjusted for age, sex, education, TBI severity, and GCS at baseline.

^b^ The confidence intervals have not been adjusted for multipicity and cannot be used to infer treatment effects.

Abbreviations: GCS, Glasgow Coma Scale; CI, Confidence Interval.

# Supplementary Table 13 Group differences of MMSE in per-protocol population.

| **Outcome** | **L-oxiracetam** | **Oxiracetam** | **Placebo** | **L-oxiracetam vs. Placebo ^a^** | | | **L-oxiracetam vs. Oxiracetam ^a,b^** |
| --- | --- | --- | --- | --- | --- | --- | --- |
|  |  |  |  | **Mean Difference**  **(95% CI)** | **Effect Size**  **(95% CI)** | **P** | **Mean Difference**  **(95% CI)** |
| **14 days** |  |  |  |  |  |  |  |
| Orientation | 1.25  (0.92,1.59) | 1.27  (0.92,1.61) | 1.37  (0.97,1.76) | -0.11  (-0.46,0.23) | -0.08  (-0.32,0.16) | 0.5133 | -0.01  (-0.29,0.26) |
| Memory | 0.38  (0.24,0.52) | 0.34  (0.20,0.49) | 0.39  (0.23,0.56) | -0.02  (-0.16,0.13) | 0.14  (-0.10,0.38) | 0.8352 | 0.03  (-0.08,0.15) |
| Attention and calculations | 0.39  (0.05,0.73) | 0.28  (-0.06,0.63) | 0.44  (0.04,0.84) | -0.05  (-0.40,0.30) | -0.01  (-0.25,0.23) | 0.7723 | 0.11  (-0.17,0.39) |
| Recall | 0.71  (0.49,0.93) | 0.76  (0.53,0.99) | 0.85  (0.59,1.11) | -0.14  (-0.37,0.09) | -0.12  (-0.35,0.12) | 0.2361 | -0.05  (-0.24,0.14) |
| Language | 0.61  (0.31,0.90) | 0.69  (0.39,0.99) | 0.72  (0.38,1.06) | -0.12  (-0.40,0.17) | -0.05  (-0.29,0.19) | 0.4324 | -0.09  (-0.32,0.15) |
| **90 days** |  |  |  |  |  |  |  |
| Orientation | 2.06  (1.76,2.36) | 1.80  (1.49,2.11) | 1.97  (1.62,2.32) | 0.09  (-0.22,0.40) | 0.05  (-0.19,0.29) | 0.5596 | 0.26  (0.02,0.51) |
| Memory | 0.46  (0.32,0.59) | 0.41  (0.27,0.55) | 0.43  (0.27,0.59) | 0.03  (-0.11,0.17) | 0.20  (-0.04,0.44) | 0.7003 | 0.05  (-0.07,0.16) |
| Attention and calculations | 0.71  (0.38,1.04) | 0.58  (0.24,0.91) | 0.41  (0.03,0.80) | 0.29  (-0.04,0.63) | 0.18  (-0.06,0.42) | 0.0878 | 0.13  (-0.14,0.40) |
| Recall | 0.72  (0.49,0.95) | 0.78  (0.54,1.02) | 0.74  (0.47,1.02) | -0.02  (-0.27,0.22) | -0.01  (-0.25,0.23) | 0.8617 | -0.06  (-0.26,0.14) |
| Language | 1.21  (0.91,1.51) | 1.12  (0.81,1.42) | 1.27  (0.93,1.62) | -0.06  (-0.36,0.23) | -0.01  (-0.25,0.23) | 0.6766 | 0.09  (-0.14,0.33) |

^a^ Models were adjusted for age, sex, education, TBI severity, and MMSE at baseline.

^b^ The confidence intervals have not been adjusted for multipicity and cannot be used to infer treatment effects.

Abbreviations: MMSE, Mini-Mental State Examination; CI, Confidence Interval.

# Supplementary Table 14 Group differences of MoCA in per-protocol population.

| **Outcome** | **L-oxiracetam** | **Oxiracetam** | **Placebo** | **L-oxiracetam vs. Placebo ^a^** | | | **L-oxiracetam vs. Oxiracetam ^a, b^** |
| --- | --- | --- | --- | --- | --- | --- | --- |
|  |  |  |  | **Mean Difference**  **(95% CI)** | **Effect Size**  **(95% CI)** | **P** | **Mean Difference**  **(95% CI)** |
| **14 days** |  |  |  |  |  |  |  |
| Executive function | 0.22  (-0.07,0.51) | 0.12  (-0.18,0.41) | 0.10  (-0.24,0.43) | 0.12  (-0.17,0.42) | 0.18  (-0.06,0.42) | 0.4146 | 0.10  (-0.14,0.34) |
| Name | 0.35  (0.18,0.52) | 0.20  (0.03,0.37) | 0.26  (0.06,0.46) | 0.09  (-0.09,0.26) | 0.16  (-0.08,0.40) | 0.3200 | 0.15  (0.01,0.29) |
| Attention | 0.50  (0.21,0.80) | 0.35  (0.06,0.65) | 0.39  (0.05,0.73) | 0.12  (-0.18,0.41) | 0.17  (-0.07,0.41) | 0.4417 | 0.15  (-0.09,0.39) |
| Language | 0.36  (0.16,0.56) | 0.38  (0.17,0.59) | 0.24  (0.01,0.48) | 0.12  (-0.09,0.33) | 0.11  (-0.12,0.35) | 0.2571 | -0.02  (-0.19,0.15) |
| Abstract | 0.28  (0.12,0.44) | 0.31  (0.15,0.48) | 0.23  (0.04,0.42) | 0.05  (-0.12,0.22) | 0.10  (-0.14,0.34) | 0.5562 | -0.03  (-0.17,0.10) |
| Delayed memory | 0.67  (0.34,1.01) | 0.54  (0.19,0.88) | 0.36  (-0.03,0.75) | 0.31  (-0.04,0.66) | 0.28  (0.03,0.52) | 0.0816 | 0.14  (-0.14,0.42) |
| Orientation | 0.72  (0.47,0.97) | 0.66  (0.41,0.92) | 0.68  (0.39,0.97) | 0.04  (-0.21,0.30) | 0.04  (-0.20,0.28) | 0.7435 | 0.06  (-0.15,0.26) |
| **90 days** |  |  |  |  |  |  |  |
| Executive function | 0.61  (0.31,0.91) | 0.62  (0.32,0.92) | 0.49  (0.14,0.83) | 0.12  (-0.18,0.43) | 0.20  (-0.04,0.44) | 0.4310 | -0.02  (-0.26,0.23) |
| Name | 0.45  (0.27,0.62) | 0.34  (0.17,0.51) | 0.36  (0.17,0.56) | 0.08  (-0.09,0.25) | 0.13  (-0.11,0.37) | 0.3609 | 0.11  (-0.03,0.25) |
| Attention | 0.45  (0.17,0.73) | 0.39  (0.11,0.67) | 0.40  (0.08,0.72) | 0.05  (-0.23,0.32) | 0.14  (-0.10,0.38) | 0.7342 | 0.06  (-0.16,0.28) |
| Language | 0.75  (0.53,0.97) | 0.68  (0.45,0.90) | 0.77  (0.52,1.03) | -0.02  (-0.25,0.20) | -0.03  (-0.27,0.21) | 0.8421 | 0.07  (-0.11,0.25) |
| Abstract | 0.37  (0.20,0.55) | 0.27  (0.08,0.45) | 0.30  (0.09,0.51) | 0.07  (-0.12,0.26) | 0.13  (-0.11,0.37) | 0.4539 | 0.11  (-0.04,0.26) |
| Delayed memory | 1.07  (0.70,1.45) | 1.07  (0.69,1.45) | 0.60  (0.16,1.04) | 0.47  (0.08,0.86) | 0.35  (0.11,0.60) | 0.0188 | 0.01  (-0.31,0.32) |
| Orientation | 1.03  (0.82,1.25) | 0.87  (0.65,1.09) | 0.92  (0.66,1.17) | 0.11  (-0.11,0.34) | 0.10  (-0.14,0.34) | 0.3113 | 0.16  (-0.01,0.34) |

^a^ Models were adjusted for age, sex, education, TBI severity, and MoCA at baseline.

^b^ The confidence intervals have not been adjusted for multipicity and cannot be used to infer treatment effects.

Abbreviations: MoCA, Montreal Cognitive Assessment; CI, Confidence Interval.

# Supplementary Table 15 Group differences of GCS in per-protocol population.

| **Outcome ^a^** | **L-oxiracetam** | **Oxiracetam** | **Placebo** | **L-oxiracetam vs. Placebo ^a^** | | | **L-oxiracetam**  **vs. Oxiracetam ^a, b^** |
| --- | --- | --- | --- | --- | --- | --- | --- |
|  |  |  |  | **Proportion Difference**  **(95% CI)** | **Effect Size**  **(95% CI)** | **P** | **Proportion Difference**  **(95% CI)** |
| **14 days** |  |  |  |  |  |  |  |
| Eye-opening reaction | 0.23  (0.21,0.25) | 0.24  (0.22,0.26) | 0.23  (0.21,0.25) | -0.00  (-0.02,0.02) | -0.04  (-0.28,0.20) | 0.9721 | -0.01  (-0.03,0.01) |
| Verbal responses | 0.16  (0.10,0.21) | 0.13  (0.07,0.18) | 0.13  (0.07,0.19) | 0.02  (-0.03,0.07) | 0.21  (-0.03,0.45) | 0.3533 | 0.03  (-0.01,0.07) |
| Motor responses | 0.09  (0.07,0.12) | 0.09  (0.07,0.11) | 0.10  (0.07,0.12) | -0.01  (-0.02,0.01) | 0.01  (-0.23,0.25) | 0.3850 | 0.00  (-0.01,0.01) |

**^a^** Models were adjusted for age, sex, education, TBI severity, and GCS at baseline.

^b^ The confidence intervals have not been adjusted for multipicity and cannot be used to infer treatment effects.

Abbreviations: GCS, Glasgow Coma Score; CI, Confidence Interval.

# Supplementary Table 16 Group differences of outcomes after imputations of missing values.

| **Outcome ^a^** | **L-oxiracetam** | **Oxiracetam** | **Placebo** | **L-oxiracetam vs. Placebo ^d^** | | | **L-oxiracetam**  **vs. Oxiracetam^d,e^** |
| --- | --- | --- | --- | --- | --- | --- | --- |
|  |  |  |  | **Mean Difference**  **(95% CI)** | **Effect Size**  **(95% CI)** | **P** | **Mean Difference**  **(95% CI)** |
| **Primary outcome** |  |  |  |  |  |  |  |
| Mean change in LOTCA at 90 days **^b^** | 20.10  (17.22,22.99) | 17.23  (14.34,20.12) | 12.48  (9.13,15.83) | 7.62  (4.70,10.54) | 0.39  (0.18,0.60) | <0.001 | 2.87  (0.48,5.26) |
| **Secondary outcomes** |  |  |  |  |  |  |  |
| Mean change in LOTCA at 14 days ^c^ | 12.60  (9.85,15.34) | 11.46  (8.72,14.21) | 7.14  (3.95,10.32) | 5.46  (2.69,8.23) | 0.33  (0.12,0.55) | 0.0001 | 1.13  (-1.14,3.41) |
| Mean change in MMSE |  |  |  |  |  |  |  |
| 14 days ^c^ | 4.45  (3.66,5.24) | 4.24  (3.45,5.02) | 4.40  (3.49,5.32) | 0.05  (-0.72,0.81) | -0.03  (-0.24,0.18) | 0.9073 | 0.21  (-0.42,0.84) |
| 90 days ^b^ | 5.46  (4.66,6.26) | 5.04  (4.25,5.84) | 4.80  (3.87,5.73) | 0.66  (-0.11,1.44) | 0.08  (-0.13,0.29) | 0.0942 | 0.42  (-0.22,1.05) |
| Mean change in MoCA |  |  |  |  |  |  |  |
| 14 days ^c^ | 3.89  (3.03,4.75) | 3.26  (2.42,4.10) | 2.99  (1.99,3.98) | 0.90  (0.05,1.75) | 0.19  (-0.02,0.40) | 0.0383 | 0.63  (-0.07,1.33) |
| 90 days ^b^ | 5.00  (4.08,5.93) | 4.51  (3.61,5.42) | 4.12  (3.06,5.19) | 0.88  (-0.03,1.79) | 0.14  (-0.07,0.35) | 0.0587 | 0.49  (-0.26,1.24) |
| Percentage of GOS-E 7–8, n (%) |  |  |  |  |  |  |  |
| 14 days ^c^ | 210(89.36) | 218(92.37) | 109(91.60) | -2.23  (-8.59,4.12) | -0.07  (-0.28,0.13) | 0.5059 | -3.01  (-8.21,2.19) |
| 90 days ^b^ | 230(97.87) | 231(97.88) | 116(97.48) | 0.39  (-2.97,3.76) | 0.03  (-0.18,0.24) | 0.8140 | -0.01  (-2.61,2.59) |
| ADL^d^ |  |  |  |  |  |  |  |
| 14 days ^c^ | 79.03  (74.91,83.15) | 77.05  (72.88,81.23) | 78.22  (73.31,83.14) | 0.81  (-3.71,5.33) | 0.05  (-0.17,0.26) | 0.7255 | 1.98  (-1.70,5.65) |
| 30 days | 93.05  (91.03,95.07) | 92.22  (90.17,94.27) | 92.53  (90.12,94.94) | 0.52  (-1.70,2.74) | 0.06  (-0.15,0.28) | 0.6465 | 0.83  (-0.98,2.63) |
| 60 days | 96.81  (95.42,98.21) | 95.96  (94.55,97.38) | 96.56  (94.90,98.23) | 0.25  (-1.28,1.78) | 0.05  (-0.16,0.26) | 0.7484 | 0.85  (-0.40,2.09) |
| 90 days ^b^ | 98.30  (97.28,99.31) | 97.65  (96.62,98.68) | 98.08  (96.87,99.30) | 0.21  (-0.90,1.33) | 0.05  (-0.16,0.27) | 0.7082 | 0.65  (-0.26,1.55) |
| Change in GCS at 14 days ^c^ | 0.49  (0.40,0.58) | 0.46  (0.37,0.55) | 0.49  (0.39,0.59) | 0.00  (-0.07,0.07) | -0.01  (-0.22,0.20) | 0.9876 | 0.03  (-0.03,0.09) |

^a^ The outcome was assessed during 14 days (end of the treatment), 30 days (first follow-up period), 60 days (second follow-up period), and 90 days (third follow-up period) after the end of treatment.

^b^ This analysis compared the data at 90 days with baseline.

^c^ This analysis compared the data at the end of treatment with baseline.

^d^ Adjusted for age, sex, education, TBI severity, and measurements of each outcome at baseline.

^e^ The confidence intervals have not been adjusted for multiplicity and cannot be used to infer treatment effects.

Fully conditional specification was used to impute the missing values of covariates and out.

Abbreviations: CI, Confidence Interval; LOTCA, Loewenstein Occupational Therapy Cognitive Assessment; MMSE, Mini-Mental State Examination; MoCA, Montreal Cognitive Assessment; GOS-E, Extended Glasgow Outcome Scale; GCS, Glasgow Coma Scale.

# Supplementary Table 17 Subgroup analysis for LOTCA in intent-to-treat population.

| **Subgroup** | **Total/**  **L-oxiracetam/ Oxiracetam/**  **Placebo** | **L-oxiracetam** | **Placebo** | **L-oxiracetam vs. Placebo ^a^** | | | **P**  **for interaction** |
| --- | --- | --- | --- | --- | --- | --- | --- |
|  |  |  |  | **Mean Difference**  **(95% CI)** | **Effect Size**  **(95% CI)** | **P** |  |
| **Sex** |  |  |  |  |  |  | 0.0376 |
| Male | 421/162/  172/87 | 18.86  (14.70,23.02) | 7.78  (3.09,12.46) | 11.09  (7.17,15.00) | 0.54  (0.29,0.80) | <0.0001 |  |
| Female | 169/73/  64/32 | 25.75  (20.35,31.15) | 22.12  (15.58,28.65) | 3.63  (-2.33,9.59) | 0.30  (-0.09,0.69) | 0.2305 |  |
| **Age** |  |  |  |  |  |  | 0.8021 |
| <65 yr | 471/184/  182/105 | 21.69  (17.79,25.58) | 11.19  (6.88,15.51) | 10.49  (6.89,14.09) | 0.53  (0.29,0.77) | <0.0001 |  |
| 65+ yr | 119/51/  54/14 | 18.22  (11.78,24.66) | 13.37  (4.11,22.62) | 4.85  (-3.87,13.57) | 0.20  (-0.30,0.69) | 0.2727 |  |
| **Education** |  |  | |  |  |  | 0.1522 |
| None | 55/22  /21/12 | 16.77  (10.59,22.96) | 14.08  (6.44,21.72) | 2.70  (-6.06,11.45) | 0.22  (-0.47,0.92) | 0.5385 |  |
| Primary school | 186/73/  75/38 | 27.52  (20.81,34.23) | 20.47  (12.63,28.31) | 7.05  (0.25,13.85) | 0.46  (0.08,0.84) | 0.0421 |  |
| Secondary or high | 349/140/  140/69 | 25.09  (21.06,29.12) | 13.63  (8.91,18.35) | 11.46  (7.44,15.48) | 0.52  (0.24,0.79) | <0.0001 |  |
| **TBI severity** |  |  |  |  |  |  | 0.0298 |
| Mild TBI | 554/221/  221/112 | 17.80  (15.55,20.05) | 9.73  (6.80,12.66) | 8.07  (4.82,11.32) | 0.43  (0.21,0.64) | <0.0001 |  |
| Moderate TBI | 36/14/  15/7 | 45.36  (33.02,57.70) | 23.19  (5.45,40.92) | 22.18  (1.12,43.23) | 1.24  (0.25,2.22) | 0.0397 |  |

^a^ Models were adjusted for age, sex, education, TBI severity, and LOTCA at baseline.

Abbreviations: LOTCA, Loewenstein Occupational Therapy Cognitive Assessment; CI, confidence interval.

# Supplementary Table 18 Additional Exploratory Subgroup Analyses of LOTCA in intent-to-treat population.

| **Subgroup** | **Total/**  **L-oxiracetam/**  **Oxiracetam/ Placebo** | **L-oxiracetam** | **Oxiracetam** | **Placebo** | **L-oxiracetam vs. Placebo ^a^** | | | **L-oxiracetam**  **vs. Oxiracetam ^a, b^** |
| --- | --- | --- | --- | --- | --- | --- | --- | --- |
|  |  |  |  |  | **Mean Difference**  **(95% CI)** | **Effect Size**  **(95% CI)** | **P** | **Mean**  **Difference**  **(95% CI)** |
| Road-traffic incident | 232/89/97/46 | 21.74  (16.61,26.86) | 17.40  (12.28,22.52) | 11.40  (5.56,17.24) | 10.34  (4.98,15.70) | 0.55  (0.20,0.90) | 0.0002 | 4.34  (0.01,8.66) |
| Ground-level fall | 117/29/39/22 | 20.27  (13.02,27.51) | 20.08  (12.64,27.51) | 13.66  (4.90,22.41) | 6.61  (-0.92,14.14) | 0.34  (-0.18,0.85) | 0.0845 | 0.19  (-5.61,6.00) |
| Fall from height | 149/65/51/33 | 17.24  (11.39,23.08) | 14.92  (9.00,20.84) | 13.36  (6.66,20.06) | 3.88  (-1.80,9.55) | 0.34  (-0.06,0.75) | 0.1790 | 2.32  (-2.64,7.27) |
| Primary injury | 450/192/167/91 | 20.07  (16.75,23.40) | 16.52  (13.14,19.90) | 11.32  (7.41,15.23) | 8.75  (5.31,12.20) | 0.48  (0.24,0.72) | <0.0001 | 3.55  (0.68,6.42) |
| Secondary injury | 28/9/12/7 | 34.16  (7.82,60.49) | 26.51  (5.96,47.07) | 20.44  (-6.62,47.51) | 13.71  (-9.43,36.86) | 0.37  (-0.69,1.43) | 0.2301 | 7.64  (-13.45,28.73) |
| ≤48 hr | 123/51/50/22 | 24.08  (16.65,31.50) | 14.27  (7.10,21.44) | 14.13  (5.03,23.23) | 9.95  (1.86,18.05) | 0.56  (0.09,1.04) | 0.0164 | 9.81  (3.50,16.12) |
| >48 to ≤72 hr | 295/122/110/63 | 20.56  (16.20,24.92) | 17.77  (13.28,22.25) | 13.05  (7.99,18.11) | 7.51  (3.13,11.89) | 0.40  (0.11,0.69) | 0.0008 | 2.80  (-0.92,6.51) |
| >72 hr | 172 | 18.05  (11.59,24.52) | 15.38  (9.17,21.59) | 7.26  (0.03,14.50) | 10.79  (4.75,16.83) | 0.56  (0.14,0.97) | 0.0005 | 2.68  (-2.30,7.65) |

^a^ Models were adjusted for age, sex, education, TBI severity, and LOTCA at baseline.

^b^ The confidence intervals have not been adjusted for multipicity and cannot be used to infer treatment effect.

Abbreviations: LOTCA, Loewenstein Occupational Therapy Cognitive Assessment; CI, confidence interval; hr, hour.
