# Supplementary material for: Efficacy and safety of L-oxiracetam on cognitive function in patients with traumatic brain injury: a multicentre, randomised, double-blind, phase 3 clinical trial
Source: Signal Transduct Target Ther. 2025 Dec 12;10:401. doi: 10.1038/s41392-025-02492-5 (PMC12698704; doi:10.1038/s41392-025-02492-5)
Supplement: Supplementary file 2 — Clinical Trial Protocol [file 41392_2025_2492_MOESM2_ESM.docx]

**Clinical Trial Protocol**

Effect of L-oxiracetam on Clinical Outcomes in Patients with Traumatic Brain Injury

The LOCATE Randomized Clinical Trial

| Clinical trial approval number: | 2016L03521 |
| --- | --- |
| Protocol Number: | NJYK-L-ORCT-III |
| Sponsor: | **Rongcai Jiang**  **Jianning Zhang** |
| Date: 01/ 09/ 2019 | |
|  |  |
| GCP Declaration  This study strictly adheres to the International Conference on Harmonisation of Technical Requirements for Registration of Pharmaceuticals for Human Use (ICH) Guideline for Good Clinical Practice (GCP) E6 (R2), applicable Chinese regulations on GCP, and the Declaration of Helsinki requirements. All required study documents will be archived in accordance with these standards. | |
| Confidentiality Statement | |
| This clinical trial protocol is a confidential document intended solely for review by researchers involved in the clinical trial, ethics committees, sponsors, Contract Research Organizations (CROs), and regulatory authorities. Distribution and disclosure outside of these entities and individuals are strictly prohibited. | |

**Protocol Synopsis**

| **Title:** L-oxiracetam on Memory and Cognitive Impairment in Mild-to-moderate Traumatic Brain Injury Patients (LOCATE): A Randomized Controlled Trial |
| --- |
| **Trial registration:** chinadrugtrials.org.cn, identifier CTR20192539; ClinicalTrials.gov, identifier NCT04205565 |
| **Intervention:** L-oxiracetam group, oxiracetam group, and placebo group |
| **Phase:** 3 |
| **Subjects:** Patients with Mild-to-moderate Traumatic Brain Injury (TBI) |
| **Objectives:**   - Primary objectives - To evaluate the efficacy of L-oxiracetam on functional outcome in patients with TBI. - To evaluate the safety of L-oxiracetam in patients with TBI. - Secondary objectives - To analyse changes in function and disease severity of patients with TBI treated with either L-oxiracetam, oxiracetam or placebo. - Exploratory objectives - To evaluate influence in subgroup (such as age, gender and so on) in patients with TBI treated with either L-oxiracetam, oxiracetam or placebo. |
| **Inclusion/ Exclusion Criteria:**   - Inclusion Criteria - Age 18-75 years, male or female; - Head injury meeting all of the following conditions: - Clear evidence of head trauma in the current diagnosis, including closed head injury or head injury with cerebrospinal fluid (CSF) leakage and/ or ear or nasal leakage and/ or intracranial air accumulation; - Confirmed by magnetic resonance imaging (MRI) or computed tomography (CT) to have intracranial bleeding above the cerebellar tentorium (including cerebral contusion, subarachnoid haemorrhage, extradural hematoma, subdural hematoma, intracerebral hematoma, etc.), with or without transient loss of consciousness; - Classified as mild to moderate head injury (Glasgow Coma Scale (GCS): 10-15); - Stable condition within 72 hours after head injury, undergoing conservative treatment, not undergoing craniotomy (may have intracranial pressure monitoring without general anesthesia or basal anesthesia); - Mini-Mental State Examination (MMSE) score below normal, with diagnostic cutoff values depending on different educational levels: illiterate (no education) ≤ 19 points, elementary school level ≤ 22 points, junior high school and above level ≤ 26 points; - Consent from the guardian and/ or patient to participate in this clinical trial and signing of the informed consent form. - Exclusion Criteria - Known or suspected allergy to the experimental drug or its components; - Use of prohibited drugs or other cognitive-enhancing drugs listed in the protocol after injury; - History of severe head trauma, cerebrovascular accidents, or structural brain lesions; - Conditions such as speech/ hearing impairment that prevent completion of cognitive function assessment; - Occurrence of secondary brain injury after the current head injury; - Need for craniotomy or external ventricular drainage; - Concurrent serious injuries to other major organs or serious complications that may affect the subject's life; - Patients with active epilepsy within the past year; - Severe liver or kidney disease with abnormal liver or kidney function tests (Alanine aminotransferase (ALT), aspartate aminotransferase (AST) ≥ 3 times the upper limit of normal, serum creatinine (Scr) > upper limit of normal); - Concurrent severe heart disease, lung disease, blood and hematopoietic system diseases, gastrointestinal diseases, or other severe or progressive systemic diseases; - History or current diagnosis of malignant tumors (excluding cured stage IB or lower cervical cancer, non-invasive basal cell or squamous cell skin cancer; exclusion of breast cancer with CR > 10 years, malignant melanoma with complete remission (CR) > 10 years, and other malignant tumors with CR > 5 years); - Presence of neurological or psychiatric disorders that prevent or unwillingness to cooperate; - Pregnant, lactating women, or those with recent plans for childbirth; - Investigator deems unsuitable for participation in the clinical trial; - Participation in another clinical trial and use of experimental drugs in the last 3 months before the trial.   **Trial Design and Duration:**  This multi-center study is a randomized, double-blind, parallel, three-arm, phase III clinical trial. The design and implementation of this trial strictly adhere to the Helsinki Declaration and have obtained approval from the ethics committees of each research center. This trial was approved by the China National Medical Products Administration (Approval Number, 2016L03521) and registered on chinadrugtrials.org.cn (identifier, CTR20192539) and ClinicalTrials.gov (identifier, NCT04205565). The rights and safety of the subjects in this trial are prioritized over the benefits to science and society.  Eligible TBI patients will be randomly assigned to three groups: L-oxiracetam group, oxiracetam group, and placebo group in a ratio of 2:2:1. The efficacy and safety of improving memory and cognitive impairment in TBI patients will be observed over a treatment period of 14 days, with a follow-up period of 90 days. |
| **Dosage Forms and Route of Administration:**  L-oxiracetam: Injectable L-oxiracetam, specification: 1g per vial, manufactured by Shenghe (China) Biopharma Co., Ltd.  Oxiracetam: Injectable oxiracetam, specification: 1g per vial, manufactured by Shiyao Group Ouyi Pharmaceutical Co., Ltd.  Placebo: Injectable L-oxiracetam or oxiracetam placebo with identical color, smell, and appearance but without active ingredients, manufactured by Shenghe (China) Biopharma Co., Ltd. |
| **Efficacy Endpoints:**   - Primary endpoint - The primary outcome measure is the change in scores on the Loewenstein Occupational Therapy Cognitive Assessment (LOTCA) at 90 days post-treatment compared to baseline. - Secondary endpoints - The secondary outcome measures include: - Changes in scores on the LOTCA and Glasgow Coma Scale (GCS) at the end of treatment compared to baseline; - Changes in scores on the Mini-Mental State Examination (MMSE) and Montreal Cognitive Assessment (MoCA) at the end of treatment and 90 days post-treatment compared to baseline; - The percentage of subjects at each level of the Glasgow Outcome Scale-Extended (GOS-E) at the end of treatment and 90 days post-treatment; - Activities of Daily Life-the Barthel Index scale (ADL-BI) at the end of treatment, 30 days, 60 days, and 90 days.   **Safety Endpoints:**   - Vital signs (temperature, pulse, respiration, blood pressure); - Laboratory tests: - Complete blood count (white blood cell (WBC), red blood cell (RBC), hemoglobin (HGB), platelet (PLT)); - Urinalysis (protein (PRO), glucose (GLU), urinary leukocyte (LEU), urine erythrocyte (ERY)); - Liver function tests (AST, ALT, total bilirubin (TBIL), gamma-glutamyl transferase (γ-GT), alkaline phosphatase (ALP)); - Renal function tests (Scr, glomerular filtration rate (GFR)); - Creatine kinase (CK); - Triglycerides (TG); - Coagulation profile (prothrombin time (PT), activated partial thromboplastin time (APTT), thrombin time (TT), fibrinogen (FIB)); |
| - 12-lead electrocardiogram (ECG); - Adverse events (AEs). |
| **Statistical Analyses:**  SAS 9.4 was used for data analysis independently conducted by a biostatistician unaware of the allocation. Continuous variables will be presented as mean ± standard deviation (SD) with a 95% confidence interval (CI) for normal distribution and as median (range) for abnormal distribution. Categorical variables will be expressed by numbers and percentages.  All hypothesis tests were two-sided with α = 0.05. A P-value ≤ 0.05 indicated statistical significance. Analysis followed the intention to treat (ITT) principle, including all randomized cases receiving at least one drug dose. For cases with incomplete treatment process, the last observed data were carried forward to the trial's final results.  When comparing the change values from baseline for each group, an analysis of covariance (ANCOVA) model was used, adjusting for baseline, injury classification, and education level. |
| **Target Sample Size:**  Before conducting this study, we conducted a pilot trial: The change values of the total scores on the LOTCA scale at 90 days post-drug cessation compared to baseline for L-oxiracetam and oxiracetam were 26.0 ± 11.8 and 22.8 ± 10.0, respectively. The combined SD for both groups was 11.0, with α set at 0.05 and 1-β at 0.8. Employing an optimal design with a sample ratio of 1:1, PASS software calculated a sample size of 190 cases for each group.  The change values of the total scores on the LOTCA scale at 90 days post-drug cessation compared to baseline for the L-oxiracetam group and the placebo group were 26.0 ± 11.8 and 20.0 ± 11.4, respectively. The combined SD for both groups was 11.6, with α set at 0.05 and 1-β at 0.8. Employing an optimal design with a sample ratio of 2:1, PASS software calculated a sample size of 204 cases for the L-oxiracetam group and 102 cases for the placebo group.  Considering potential dropouts, we increased the sample size by 15%. Therefore, the sample sizes for the L-oxiracetam group, oxiracetam group, and placebo group were set at 236, 236, and 118, respectively. |
| **Trial Period:**  September 2019- May 2024. |

**Trial Schedule**

|  | **Medication Period** | | **Follow-up**^§^  **(± 3 days)** | | |
| --- | --- | --- | --- | --- | --- |
| **Visit window** | Screening | Treatment Period  (14 days + 1 day) | Day  30 | Day  60 | Day  90 |
| **General Procedures** | | | | | |
| Informed consent | X |  |  |  |  |
| Medical history | X |  |  |  |  |
| General data | X |  |  |  |  |
| Acute phase treatment | X |  |  |  |  |
| Inclusion/ Exclusion criteria | X |  |  |  |  |
| Concomitant diseases and medications | X |  |  |  |  |
| **Screening Indicators** | | | | | |
| Pregnancy test  (for women of childbearing age) | X |  |  |  | X |
| CT or MRI | X^‡^ |  |  |  | X |
| **Safety** | | | | | |
| Vital signs | X | X |  |  | X |
| Laboratory tests^†^ | X^‡^ | X |  |  | X |
| 12-lead electrocardiogram | X^‡^ | X |  |  | X |
| **Efficacy** | | | | | |
| MMSE | X | X |  |  | X |
| GCS | X | X |  |  |  |
| GOS-E |  | X |  |  | X |
| MoCA | X | X |  |  | X |
| LOTCA | X | X |  |  | X |
| ADL-BI |  | X | X | X | X |
| **Other Tasks** | | | | | |
| Allocation of drug numbers | X |  |  |  |  |
| Dispensing experimental drugs | X |  |  |  |  |
| Retrieval of remaining drugs |  | X |  |  |  |
| Recording concomitant medications |  | X | X | X | X |
| Adverse event recording |  | X | X | X | X |
| Efficacy assessment |  | X |  |  | X |
| Summary of trial completion status |  |  |  |  | X |

Abbreviations: ADL-BI, Activities of Daily Life-the Barthel Index scale; CT, computed tomography; GCS, Glasgow Coma Scale; GOS-E, Glasgow Outcome Scale-Extended; LOTCA, Loewenstein Occupational Therapy Cognitive Assessment; MMSE, Mini-Mental State Examination; MoCA, Montreal Cognitive Assessment; MRI, magnetic resonance imaging.

**Study timeline**

| **Major Study Milestones** | **2019** | **2020** | **2021** | **2022** | **2023** | **2024** | **2025** |
| --- | --- | --- | --- | --- | --- | --- | --- |
|  | **9-12 months** |  |  |  |  |  |  |
| **Site Selection** |  |  |  |  |  |  |  |
| **Ethics Application** |  |  |  |  |  |  |  |
| **Site Training** |  |  |  |  |  |  |  |
| **Recruitment and Intervention** |  |  |  |  |  |  |  |
| **Endpoint Evaluation** |  |  |  |  |  |  |  |
| **Data Cleaning and Closure** |  |  |  |  |  |  |  |
| **Analysis and Results** |  |  |  |  |  |  |  |
| **Presentation and Publication** |  |  |  |  |  |  |  |

[**Protocol Synopsis** 3](#_Toc179820024)

[**List of Abbreviation** 17](#_Toc179820025)

[**1 Introduction** 19](#_Toc179820026)

[**1.1 Traumatic Brain Injury** 19](#_Toc179820027)

[1.1.1 Epidemiological Overview of Traumatic Brain Injury 19](#_Toc179820028)

[1.1.2 Cognitive Impairments Related to TBI 19](#_Toc179820029)

[**1.2 Introduction to L-Oxiracetam for Injection** 21](#_Toc179820030)

[**1.3 Preclinical Trial Results for L-Oxiracetam for Injection** 23](#_Toc179820031)

[1.3.1 Main Pharmacological Study Results 23](#_Toc179820032)

[1.3.1.1 Effect on Brain Slice Long-Term Potentiation 23](#_Toc179820033)

[1.3.1.2 Protective Effects Against Neuronal Damage 23](#_Toc179820034)

[1.3.1.3 Effect on Secretion of Acetylcholine by Cholinergic Neurons 24](#_Toc179820035)

[1.3.1.4 Effect on Learning and Memory Functions in Rats with Traumatic Brain Injury Caused by Hydraulic Shock 24](#_Toc179820036)

[1.3.1.5 Effect on Learning and Memory Functions in Rats with TBI Caused by Free Fall 25](#_Toc179820037)

[1.3.1.6 Effect on Learning and Memory Impairments in Mice Induced by Scopolamine 26](#_Toc179820038)

[1.3.1.7 Effect on Learning and Memory Impairments in Mice with Vascular Dementia 26](#_Toc179820039)

[1.3.2 Main Toxicological Study Results 27](#_Toc179820040)

[1.3.2.1 Acute Toxicity Test 27](#_Toc179820041)

[1.3.2.2 Long-Term Toxicity Test 27](#_Toc179820042)

[1.3.2.3 Special Safety Tests (Allergenicity, Hemolysis, Local Irritancy, etc.) 28](#_Toc179820043)

[1.3.2.4 Mutagenicity Test 28](#_Toc179820044)

[1.3.3 Non-Clinical Pharmacokinetic Study Results 29](#_Toc179820045)

[1.3.3.1 Pharmacokinetic Parameters of L-Oxiracetam 29](#_Toc179820046)

[1.3.3.2 Tissue Distribution of L-Oxiracetam 29](#_Toc179820047)

[1.3.3.3 Chiral Conversion between L-Oxiracetam and R-Oxiracetam 29](#_Toc179820048)

[1.3.3.4 Comparative Study of the Pharmacokinetic Behavior and Tissue Distribution between L-Oxiracetam and Racemic Oxiracetam 30](#_Toc179820049)

[1.3.3.5 Plasma Protein Binding, Metabolic Pathways, and Excretion Routes of L-Oxiracetam 30](#_Toc179820050)

[1.3.4 Clinical Research Results 30](#_Toc179820051)

[1.3.4.1 Phase I Clinical Human Tolerability Test 30](#_Toc179820052)

[1.3.4.2 Phase I Clinical Human Pharmacokinetic Test 31](#_Toc179820053)

[1.3.4.3 Phase II Clinical Trials for Efficacy and Safety Evaluation 32](#_Toc179820054)

[**1.4 Potential Risks and Benefits** 33](#_Toc179820055)

[1.4.1 Known Potential Risks 33](#_Toc179820056)

[1.4.1.1 International Research Results 33](#_Toc179820057)

[1.4.1.2 Domestic Research Results 33](#_Toc179820058)

[1.4.2 Known Potential Benefits 34](#_Toc179820059)

[1.4.2.1 International Related Clinical Research 34](#_Toc179820060)

[1.4.2.2 Domestic Related Clinical Research 35](#_Toc179820061)

[**2 Trial Objectives** 37](#_Toc179820062)

[**3 Trial Design** 38](#_Toc179820063)

[**3.1 Summary of Trial Design** 38](#_Toc179820064)

[**3.2 Trial Period** 38](#_Toc179820065)

[**4 Target Population** 39](#_Toc179820066)

[**4.1 Eligibility of Target Population** 39](#_Toc179820067)

[**4.2 Inclusion and exclusion criteria** 39](#_Toc179820068)

[4.2.1 Inclusion criteria 39](#_Toc179820069)

[4.2.2 Exclusion criteria 39](#_Toc179820070)

[**4.3 Case criteria for discontinuation/ withdrawal** 40](#_Toc179820071)

[4.3.1 Termination/ Withdrawal Decided by the Investigator 40](#_Toc179820072)

[4.3.2 Withdrawal Initiated by the Subject 40](#_Toc179820073)

[4.3.3 Handling of Cases of Termination/ Withdrawal 41](#_Toc179820074)

[**4.4 Criteria for Stopping the Trial** 41](#_Toc179820075)

[**5 Trial Procedures and Assessment Methods** 42](#_Toc179820076)

[**5.1 Assessment Procedures** 42](#_Toc179820077)

[**5.2 Drug Provision** 42](#_Toc179820078)

[**5.3 Drug Packaging and Labeling** 42](#_Toc179820079)

[**5.4 Drug Storage and Stability** 42](#_Toc179820080)

[**5.5 Preparation of Formulations** 42](#_Toc179820081)

[**5.6 Trial Period** 42](#_Toc179820082)

[**5.7 Trial Drug Accountability Procedure** 42](#_Toc179820083)

[**5.8 Trial Procedure Related Assessments** 43](#_Toc179820084)

[**5.9 Scale evaluation** 44](#_Toc179820085)

[**5.10 Clinical Laboratory Evaluation** 45](#_Toc179820086)

[**5.11 Screening Period (-72h to 0h)** 45](#_Toc179820087)

[**5.12 Treatment Period (14 days + 1 day)** 46](#_Toc179820088)

[**5.13 Follow-Up Visits** 46](#_Toc179820089)

[**5.14 Unscheduled Visits** 47](#_Toc179820090)

[**6 Concomitant Medications and Treatments** 48](#_Toc179820091)

[**6.1 Basic Treatment** 48](#_Toc179820092)

[**6.2 Prohibited Medications and Treatments** 48](#_Toc179820093)

[**6.3 Permitted Medications and Treatments** 48](#_Toc179820094)

[**7 Endpoints evaluation criteria** 49](#_Toc179820095)

[**7.1 Efficacy Endpoints:** 49](#_Toc179820096)

[7.1.1 Primary endpoint 49](#_Toc179820097)

[7.1.2 Secondary endpoints 49](#_Toc179820098)

[**7.2 Safety Endpoints:** 49](#_Toc179820099)

[**8 Safety assessment** 50](#_Toc179820100)

[**8.1 Definitions** 50](#_Toc179820101)

[**8.2 Safety Information Related to the Trial Medication** 50](#_Toc179820102)

[**8.3 Management and Documentation of** **Adverse Events** 50](#_Toc179820103)

[8.3.1 Management of Adverse Events 50](#_Toc179820104)

[8.3.2 Recording of AEs 51](#_Toc179820105)

[**8.4 Severity grading of AEs** 51](#_Toc179820106)

[**8.5 Assessment of the Relationship between AEs and the Investigational Drug** 52](#_Toc179820107)

[**8.6 Follow-up of Adverse Event Outcomes** 53](#_Toc179820108)

[**8.7 Serious AEs** 53](#_Toc179820109)

[8.7.1 Management of SAEs 53](#_Toc179820110)

[8.7.2 Reporting of SAEs 53](#_Toc179820111)

[8.7.3 Pregnancy 54](#_Toc179820112)

[8.7.4 Hospitalization 54](#_Toc179820113)

[**9 Data Management** 56](#_Toc179820114)

[**9.1 Developing a Data Management Plan** 56](#_Toc179820115)

[**9.2 Database Creation** 56](#_Toc179820116)

[**9.3 Data Entry** 56](#_Toc179820117)

[**9.4 Data Verification** 56](#_Toc179820118)

[**9.5 Database Lock** 56](#_Toc179820119)

[**9.6 Data document preservation and data transfer** 57](#_Toc179820120)

[**10 Sample size calculation** 58](#_Toc179820121)

[**11 Blinding** 59](#_Toc179820122)

[**12 Randomization** 60](#_Toc179820123)

[**13 Statistical Analysis of Datasets: Definition and Selection** 61](#_Toc179820124)

[**14 Statistical analysis plan** 62](#_Toc179820125)

[**15 Quality Control and Assurance** 63](#_Toc179820126)

[**15.1 Quality Control Measures** 63](#_Toc179820127)

[**15.2 Training of Researchers** 63](#_Toc179820128)

[**15.3 Enhancing Participant Compliance** 63](#_Toc179820129)

[**15.4 Clinical Trial Monitoring** 63](#_Toc179820130)

[**15.5 Clinical Trial Auditing** 63](#_Toc179820131)

[**16 Ethical Requirements** 65](#_Toc179820132)

[**17 Clinical Trial Records** 66](#_Toc179820133)

[**18 Protocol Revision** 67](#_Toc179820134)

[**Appendix 1. Participating hospital** 68](#_Toc179820135)

[**Appendix 2 Informed Consent Form · Notification Page** 70](#_Toc179820136)

[**Appendix 3. Scoring Criteria Attachments** 81](#_Toc179820137)

[**Attachment 1. Mini-Mental State Examination (MMSE)** 81](#_Toc179820138)

[**Attachment 2. Montreal Cognitive Assessment (MoCA）** 86](#_Toc179820139)

[**Attachment 3. Loewenstein Occupational Therapy Cognitive Assessment (LOTCA)** 93](#_Toc179820140)

[**Attachment 4. Glasgow Outcome Scale Extended (GOS-E)** 111](#_Toc179820141)

[**Attachment 5. Glasgow Coma Scale (GCS)** 112](#_Toc179820142)

[**Attachment 6. Activities of Daily Living (ADL) Ability Scale (Barthel Index)** 113](#_Toc179820143)

**List of Abbreviation**

| TBI | Traumatic brain injury |
| --- | --- |
| CROs | Contract Research Organizations |
| CSF | Cerebrospinal fluid |
| MRI | Magnetic resonance imaging |
| CT | Computed tomography |
| GCS | Glasgow Coma Scale |
| MMSE | Mini-Mental State Examination |
| ALT | Alanine aminotransferase |
| AST | Aspartate aminotransferase |
| Scr | Serum creatinine |
| CR | Complete remission |
| LOTCA | Loewenstein Occupational Therapy Cognitive Assessment |
| MoCA | Montreal Cognitive Assessment |
| GOS-E | Glasgow Outcome Scale-Extended |
| ADL-BI | Activities of Daily Life-the Barthel Index scale |
| ADL | Activities of Daily Living |
| WBC | White blood cell |
| RBC | Red blood cell |
| HGB | Hemoglobin |
| PLT | Platelet |
| PRO | Protein |
| GLU | Glucose |
| LEU | Urinary leukocyte |
| ERY | Urine erythrocyte |
| TBIL | Total bilirubin |
| γ-GT | Gamma-glutamyl transferase |
| ALP | Alkaline phosphatase |
| GFR | Glomerular filtration rate |
| CK | Creatine kinase |
| TG | Triglyceride |
| PT | Prothrombin time |
| APTT | Activated partial thromboplastin time |
| TT | Thrombin time |
| FIB | Fibrinogen |
| ECG | Electrocardiogram |
| EEG | Electroencephalogram |
| SD | Standard deviation |
| CI | Confidence interval |
| ITT | Intention-to-treat |
| ANCOVA | Analysis of covariance |
| CDC | Centers for Disease Control and Prevention |
| WHO | World Health Organization |
| ATP | Adenosine triphosphate |
| RNA | Ribonucleic acid |
| LTP | Long-term potentiation |
| NSCs | Neural stem cells |
| ACh | Acetylcholine |
| MLD | Minimum lethal dose |
| MTD | Maximum tolerated dose |
| PCT | Plateletcrit |
| CL | Plasma clearance |
| AUC | Area Under the Curve |
| steady-state AUC | AUCsst |
| Cmax | Peak concentration |
| ELISA | Enzyme linked immunosorbent assay |
| PPS | Per Protocol Set |
| CDP-Choline | Cytidine diphosphate choline |
| eCRF | electronic Case Report Form |
| AE | Adverse event |
| SAE | Serious adverse event |
| SUSAR | Suspected unexpected serious adverse reaction |
| GCP | Good Clinical Practice |
| CRCs | Clinical Research Coordinators |
| IWRS | Interactive Web Response System |
| FAS | Full analysis set |
| SS | Safety analysis set |

**1 Introduction**

**1.1 Traumatic Brain Injury (TBI)**

1.1.1 Epidemiological Overview of TBI ^1-6^

With the advancement of modern industry, power machinery, and high-speed transportation, the incidence of TBIs has been steadily increasing. During peacetime, such injuries are the second most common type of trauma, following limb injuries, with an incidence rate of 22% to 42%. Moreover, TBIs lead in terms of mortality rate, accounting for 72.2% to 92.5% of all trauma-related deaths. Although the overall mortality rate for TBIs has decreased from 50% three decades ago to approximately 30% today, about 10% of survivors with mild injuries sustain permanent disabilities. This proportion rises to 66% for those with moderate injuries and 100% for those with severe injuries. The physical disabilities, cognitive impairments, and behavioral and emotional disturbances resulting from TBIs impose significant burdens on society and families. Cognitive dysfunction, in particular, is prominently evident among the disabilities caused by brain trauma. The extent of cognitive recovery directly influences the improvement of other impairments and the overall enhancement of quality of life.

The incidence of TBIs varies across different countries and regions, influenced by disparities in inclusion criteria for studies and research methodologies. A survey conducted by the U.S. Centers for Disease Control and Prevention (CDC) reveals that each year in the United States, approximately 1.7 million people experience TBIs, resulting in 52,000 deaths, 275,000 hospitalizations, and nearly 1,365,000 emergency department visits. Deaths from TBIs account for 30.5% of all injury-related fatalities in the U.S. According to the World Health Organization (WHO), the global incidence of mild TBIs ranges from 100 to 300 per 100,000 people. In Europe and North America, the incidence rates are between 47 and 453 per 100,000 and 51 and 618 per 100,000, respectively. In Oceania, New Zealand has an incidence rate of approximately 790 per 100,000. In China, the incidence rate is around 100 per 100,000, with rates in Western China exceeding the national average at 150 to 200 per 100,000. Estimates of TBI incidence primarily rely on official data, which may be biased. This is because only a portion of patients who visit hospitals are recorded, leading to an underestimation of the actual incidence rates. The actual rates of TBI are likely much higher than anticipated. Road traffic accidents are the primary cause of TBIs, accounting for 20% to 65% of cases. An epidemiological survey conducted in six provinces in Northeast China in 2004 found that road traffic injuries were the leading cause of TBIs, accounting for 61% of cases.

1.1.2 Cognitive Impairments Related to TBI

Cognition refers to the psychological processes involved in recognizing and understanding objects and events, including perception, attention, learning, memory, thinking, and language. Cognitive functions are composed of multiple domains, including memory, computation, time and spatial orientation, constructional abilities, executive functions, language comprehension, and expression, among others. Cognitive dysfunction broadly refers to impairments in cognitive functions caused by various factors, ranging from mild cognitive impairment to dementia. This condition is also known as cognitive decline, cognitive deficit, or cognitive disability.

Cognitive impairment is one of the most common and enduring sequelae of TBI. The mechanisms causing cognitive dysfunction are highly complex and are not yet fully understood.^7-12^ Based on existing information, the mechanisms behind cognitive impairments can roughly be divided into two aspects: the destruction of brain tissue structures related to cognition and abnormalities in the neurotransmitter system. Many parts of the brain are involved in the normal expression of cognitive functions, such as the frontal lobes, temporal lobes, parietal lobes, and hippocampus. Early research discovered that cognitive impairments caused by damage to the right hemisphere are more severe, with particularly prominent effects from damage to the ventral and medial cortices of the frontal lobes. The frontal lobes are mainly associated with executive functions, including abstract ability, concept formation, selective memory, and the ability to transfer cognitive processes. The hippocampus is closely related to learning and memory functions; damage to this area can impact the acquisition of new knowledge and the retrieval of previously mastered information. Additionally, the parietal lobe may also be involved in certain cognitive activities. Recent studies suggest that the cerebellum might also participate in higher cortical functions such as cognition and emotion, although definitive evidence is still lacking. Moreover, neurotransmitters related to cognitive functions include acetylcholine, dopamine, norepinephrine, serotonin, gamma-aminobutyric acid, and glutamate. It is currently believed that changes in neurotransmitters during the acute phase of brain injury may temporarily affect cognition, while persistent cognitive impairments during the recovery phase are related to permanent brain tissue damage and long-term changes in the function of the brain's neurotransmitter systems.

The treatment of cognitive impairments following TBI includes pharmacotherapy, hormone replacement therapy, and cognitive rehabilitation.^13-15^

Pharmacotherapy: Currently, there is a lack of large-scale randomized controlled trials and evidence-based medicine supporting pharmacological treatment for cognitive impairments following TBI. The existing pharmacological treatments are based on experiences from small-scale studies or from research on cognitive impairments caused by other types of brain damage, such as Alzheimer's disease and vascular dementia. The medications used include: (1) Drugs primarily acting on the dopaminergic system: Bromocriptine, Levodopa, Methylphenidate, and Amphetamine; (2) Drugs primarily acting on the catecholaminergic system: Meclofenoxate Hydrochloride, Methylphenidate Hydrochloride and Modafinil; (3) Drugs primarily acting on the cholinergic system: Physostigmine, Tacrine, Donepezil, Rivastigmine, and Galantamine; (4) Other drugs: Citicoline and Lamotrigine.

Hormone Replacement Therapy: (1) Growth Hormone: Growth hormone plays a critical role in the growth and development of the central nervous system, particularly evident in promoting brain growth, myelination, differentiation of glial cells, and cognitive functions; (2) Estrogen: Estrogens can increase the synthesis and transmission of neurotransmitters such as acetylcholine, thereby significantly influencing the normal functioning of learning, memory, and cognitive processes; (3) Thyroid Hormone: Thyroid hormones regulate the expression of genes and proteins related to myelination, neuronal migration, differentiation, and maturation. This includes the expression of proteins such as Neural Growth Protein-43, neurogranins, calmodulin-dependent protein kinase, and extracellular signal-regulated kinases, thereby affecting brain functions including learning and memory.

Cognitive Rehabilitation: Cognitive rehabilitation is a therapeutic approach designed to enhance cognitive function in patients with brain injuries. Current methods of cognitive rehabilitation primarily include occupational therapy, implicit memory rehabilitation, errorless learning, cognitive neuropsychological rehabilitation, computer-assisted and virtual cognitive rehabilitation, remote-controlled cognitive rehabilitation via the internet, and electromagnetic stimulation.

**1.2 Introduction to L-Oxiracetam for Injection**

Nootropics are a novel class of central nervous system drugs that enhance learning and memory capabilities. Unlike psychotropic drugs such as sedatives, antidepressants, anti-anxiety medications, psychostimulants, and hallucinogens, nootropics operate by assimilating the metabolic processes of brain cell bioenergy (such as glucose, ATP (Adenosine triphosphate), proteins, RNA (Ribonucleic acid), lipids, etc.). They selectively target the cerebral cortex and hippocampus to protect, activate, or promote the recovery of neuronal functions, thereby improving brain integration mechanisms related to cognitive behaviors (such as memory, learning, problem-solving, and analytical abilities). Currently, the most notable nootropics belong to the pyrrolidone class, with oxiracetam being a prominent example. Oxiracetam, a synthetic cyclic derivative of hydroxyamino butyric acid, also known as acetoxyoxiracetam or hydroxyoxopyrrolidine acetamide, was first synthesized by the Italian company Sigma-Tau Pharmaceuticals in 1974. It was initially launched in Italy in 1987, followed by a release in Portugal in 1991. In 2003, the China Food and Drug Administration approved the domestic release of oxiracetam capsules by Shijiazhuang Yiling Pharmaceutical Co., Ltd. Subsequently, in 2005 and 2010, approvals were also granted for oxiracetam injectables and injectable oxiracetam for domestic distribution.

L-oxiracetam, the single levorotatory enantiomer of oxiracetam, has not been marketed domestically or internationally. It has shown significant enhancement in learning and memory functions in rats subjected to acute brain damage caused by hydraulic shock or free fall. Furthermore, it significantly enhances and improves memory functions in mice with vascular dementia induced by repeated ligation and reperfusion, and in mice with learning and memory disorders caused by scopolamine. L-oxiracetam promotes the production of long-term potentiation (LTP) in the brain, increasing the duration and amplitude of post-synaptic potentials. It exhibits acetylcholine agonistic activity, preventing or reversing the reduction in learning and memory caused by scopolamine, and promotes the secretion of acetylcholine from primary cultured septal cholinergic neurons. Additionally, it enhances the brain's oxygen utilization, increases ATP content in the injured cortical and hippocampal areas, promotes the regeneration of new hippocampal neurons, and reduces cell apoptosis induced by brain damage.

Injectable oxiracetam is indicated for memory and cognitive impairments caused by conditions such as brain trauma. Clinically, adverse reactions include anxiety, skin itching, rash, nausea, and stomach pain, which can subside after discontinuation of the drug. A reduction in dosage should be considered in cases where patients experience psychiatric excitation or sleep disturbances. Caution is advised in patients allergic to oxiracetam and those with renal impairment.

Oxiracetam has been widely used both domestically and internationally for many years, with confirmed therapeutic efficacy and good patient tolerance. Extensive clinical research within the country has shown that oxiracetam can significantly improve memory and cognitive impairments caused by conditions such as brain trauma, with effects superior to those of piracetam. L-oxiracetam has shown notable improvement in cognitive and memory functions in rats with TBIs caused by hydraulic shock and free fall, and the efficacy of 200 mg/ kg L-oxiracetam is comparable to that of 400 mg/ kg oxiracetam. Pharmacokinetic studies have shown that there is no significant stereoisomer conversion between L-oxiracetam and R-oxiracetam in rats and beagles. The main pharmacokinetic parameters of L-oxiracetam in the plasma of rats and beagles showed no significant differences after a single intravenous injection of L-oxiracetam and twice the dose of racemic oxiracetam. The tissue distribution of L-oxiracetam was essentially the same whether administered alone or following the administration of racemic oxiracetam. Results from safety pharmacology, acute toxicity, chronic toxicity, genotoxicity, and local toxicity tests indicate that, at equivalent doses, L-oxiracetam and oxiracetam show no significant difference in toxicity to the test animals or cells. These preclinical results suggest that L-oxiracetam is the primary active component of oxiracetam in vivo. The use of L-oxiracetam alone may reduce the clinical dosage required and lower the potential for toxic side effects.

**1.3 Preclinical Trial Results for L-Oxiracetam for Injection**

1.3.1 Main Pharmacological Study Results

1.3.1.1 Effect on Brain Slice Long-Term Potentiation

LTP in the hippocampus is currently considered the primary mechanism for memory formation. In this experiment, electrophysiological techniques were employed on hippocampal brain slices to induce LTP using both L-oxiracetam and R-oxiracetam, in order to compare their pharmacological efficacy. The results indicate that L-oxiracetam, at a concentration of 10^-6^ M, can induce an increase in LTP amplitude (72.8 ± 16.2%), which is significantly higher than that induced by R-oxiracetam (28.7 ± 6.2%). The difference between the two isomers became even more pronounced at a concentration of 10^-7^ M, with LTP amplitude increases of 48.9 ± 9.1% for L-oxiracetam and 5.8 ± 3.2% for R-oxiracetam.

1.3.1.2 Protective Effects Against Neuronal Damage

Method: Neural stem cells (NSCs) were isolated from the cortical brain tissue of rat embryos and expanded. The subcultures of NSCs were carried through to the fourth generation and were plated for differentiation in a controlled culture setting. The experimental groups were designated as follows: a blank control group (contr), a normal saline group (ns), a R-oxiracetam group (R), low (Sl), medium (Sm), and high (Sh) dose L-oxiracetam groups, and an oxiracetam group (R/ S). On the 11th day of the culture medium exchange, the contr group was supplemented with DMEM/ F12 serum-free medium containing 20μl/ ml of normal cortical brain extract. The ns group received a DMEM/ F12 serum-free medium containing 20μl/ ml of brain injury cortical extract and 10μl/ ml of normal saline. The other groups were administered a medium containing 20μl/ ml of brain injury cortical extract along with varying concentrations of the respective drugs: R-oxiracetam at 2mg/ ml, L-oxiracetam at low dose (0.5mg/ ml), medium dose (1mg/ ml), high dose (2mg/ ml), and oxiracetam at 4mg/ ml. The cultures were maintained for an additional 4 days before undergoing MAP-2 immunofluorescence staining and TUNEL apoptosis assays.

Results: The immunofluorescence staining for MAP-2 indicated that the number of differentiated MAP-2 positive neurons was higher in the contr, Sh, and R/ S groups compared to the other groups. There was a statistically significant difference between the Sh, R/ S groups and the ns, R groups, and the number of differentiated MAP-2 positive neurons increased with higher doses of L-oxiracetam. TUNEL apoptosis assay results showed that the contr, Sh, and R/ S groups had fewer TUNEL positive apoptotic cells compared to other groups. There was a statistically significant difference between the Sh, R/ S groups and the ns, R groups, and the number of TUNEL positive apoptotic cells decreased as the dose of L-oxiracetam increased.

1.3.1.3 Effect on Secretion of Acetylcholine by Cholinergic Neurons

Method: Embryonic mouse forebrain primordia were used to prepare a single cell suspension, which was seeded onto 24-well plates with poly-lysine-coated cover slips. There were seven groups, with six wells per group, and each well received 1.5ml of medium. The contr group received DMEM/ F12 serum-free medium; the ns group received DMEM/ F12 serum-free medium containing 10μl/ ml normal saline. The other groups were supplemented with DMEM/ F12 serum-free medium containing the respective drug concentrations. The cultures were maintained for 14 days. Acetylcholine (ACh) content in the culture supernatants was measured using an enzyme linked immunosorbent assay (ELISA), and the cultured cells were subjected to ChAT immunofluorescence staining.

Results: ChAT immunofluorescence labeling showed that cultured primary cholinergic neurons from the basal forebrain primordium displayed numerous ChAT positive neurons. There were no statistically significant differences in the number of cholinergic neurons among the groups. However, the ChAT immunofluorescence intensity was significantly higher in the Sh and R/ S groups compared to the contr, ns, and R groups. Analysis of ACh content in the culture medium showed that the levels of acetylcholine were higher in the Sh and R/ S groups than in the other groups. There was a statistically significant difference between the Sh, R/ S groups and the ns, R groups, and the acetylcholine content in the culture medium increased with higher doses of L-oxiracetam.

1.3.1.4 Effect on Learning and Memory Functions in Rats with TBI Caused by Hydraulic Shock

Method: Rats that met the criteria through water maze training were selected and used to create a controllable hydraulic brain injury model. They were then randomly divided into several groups: a blank control group (contr), a normal saline group (ns), a R-oxiracetam group (R), and L-oxiracetam groups at low (Sl), medium (Sm), and high (Sh) doses, as well as an oxiracetam group (R/ S). The contr group received no treatment; the ns group was given sterile normal saline 1ml intravenously for 14 days post-injury; the drug treatment groups received sterile normal saline containing respective drug concentrations (R 200mg/ kg, Sl 50mg/ kg, Sm 100mg/ kg, Sh 200mg/ kg, R/ S 400mg/ kg) intravenously for 14 days post-injury. Spatial exploration tests were conducted 15 days post-injury, location cruising tests over 4 days started on the 16th day post-injury, and a second spatial exploration test was carried out on the 20th day post-injury. Subsequently, brain tissues from some of the rats in the brain injury model were perfused and sectioned for ChAT and DCX immunofluorescence analysis. Other rats from the hydraulic brain injury model had the injured cortical and hippocampal regions sampled for ATP content analysis.

Results: The spatial exploration test results indicated that the contr, Sh, and R/ S groups had a higher number of platform crossings compared to the other groups. There were statistically significant differences between the Sh, R/ S groups and the ns, R groups, and as the dose of L-oxiracetam increased, the number of platform crossings by the rats also significantly increased. In the location cruising test, the escape latency was shorter in the contr, Sh, and R/ S groups compared to the other groups, with significant differences observed between the Sh, R/ S groups and the ns, R groups. Additionally, as the dose of L-oxiracetam increased, the escape latency of the rats significantly decreased. Immunofluorescence labeling for ChAT in the septal areas showed that the contr, Sh, and R/ S groups had more ChAT-positive cholinergic neurons on the injured side than the other groups, with significant differences between the Sh, R/ S groups and the ns, R groups. The number of ChAT-positive cholinergic neurons on the injured side in the septal areas also increased with higher doses of L-oxiracetam. Immunofluorescence labeling for ChAT in the Meynert basal nucleus indicated that the contr, Sh, and R/ S groups had more ChAT-positive cholinergic neurons on the injured side compared to the other groups. There were statistically significant differences between the Sh, R/ S groups and the ns, R groups, and the number of ChAT-positive cholinergic neurons in the injured side of the Meynert basal nucleus also increased with higher doses of L-oxiracetam. Immunofluorescence results for DCX in the hippocampus showed that the Sh and R/ S groups had more DCX-positive new neurons in the dentate gyrus of the injured side hippocampus compared to other groups. The differences between the Sh, R/ S groups and the ns, R groups were statistically significant, and the number of DCX-positive new neurons in the dentate gyrus of the injured side hippocampus also increased with higher doses of L-oxiracetam. ATP content analysis in the cortex and hippocampus showed that the contr, Sh, and R/ S groups had higher ATP contents in the injured side cortex and hippocampus compared to the other groups. There were statistically significant differences between the Sh, R/ S groups and the ns, R groups, and the ATP content in the cortex and hippocampus also increased with increasing doses of L-oxiracetam.

1.3.1.5 Effect on Learning and Memory Functions in Rats with TBI Caused by Free Fall

Method: Rats that successfully passed water maze training were used to create a free fall-induced brain injury model. The animals were randomly divided into several groups: a blank control group (contr), a normal saline group (ns), a R-oxiracetam group (R), and low (Sl), medium (Sm), and high (Sh) dose L-oxiracetam groups, along with an oxiracetam group (R/ S). The contr group received no treatment. The ns group received sterile normal saline 1ml via tail vein injection daily for 14 days post-injury. The other treatment groups received daily tail vein injections of sterile normal saline containing the respective drug concentrations (R 200mg/ kg, Sl 50mg/ kg, Sm 100mg/ kg, Sh 200mg/ kg, R/ S 400mg/ kg) for 14 days post-injury. On the 15th day post-injury, the first spatial exploration test was conducted, followed by a 4-day place navigation test starting on the 16th day, and a second spatial exploration test on the 20th day post-injury.

Results: The results of the spatial exploration tests showed that the contr, Sh, and R/ S groups had a higher number of platform crossings compared to other groups, with significant differences observed between the Sh, R/ S groups and the ns, R groups. As the dose of L-oxiracetam increased, the number of platform crossings also significantly increased. In the place navigation tests, the escape latency was shorter for the contr, Sh, and R/ S groups compared to other groups. Significant differences were noted between the Sh, R/ S groups and the ns, R groups, and as the dose of L-oxiracetam increased, the escape latency of the rats significantly decreased.

1.3.1.6 Effect on Learning and Memory Impairments in Mice Induced by Scopolamine

Results from the Avoidance of Darkness Test: The high-dose group of L-oxiracetam significantly prolonged the latency period of memory in the model mice. The medium (150 mg/ kg) and high (300 mg/ kg) dose groups significantly reduced the number of errors made (P < 0.05). Results from the Y-Maze Test: The high-dose group of L-oxiracetam decreased the number of training sessions required for the model mice to escape to the correct arm; both medium and high dose groups significantly increased the number of proactive escapes and improved accuracy rates (P < 0.05, P < 0.01). In these experiments, the efficacy of the high-dose group was comparable to the oxiracetam group and better than the R-oxiracetam group, although the differences were not statistically significant (P > 0.05).

Results from the Jumping Platform Test: All dose groups of L-oxiracetam reduced the number of memory errors in the model mice. The high-dose group notably extended the latency period of memory (P < 0.05). Results from the Morris Water Maze Test: The medium and high dose groups of L-oxiracetam significantly shortened the latency period in the location navigation trial (P < 0.05); all dose groups significantly improved the number of original platform crossings in the spatial exploration test compared to the model group, with significant differences (P < 0.05, P < 0.01). In these experiments, the efficacy of the high-dose group was comparable to the oxiracetam group and superior to the R-oxiracetam group (P < 0.05).

1.3.1.7 Effect on Learning and Memory Impairments in Mice with Vascular Dementia

In the study, the medium (150 mg/ kg) and high (300 mg/ kg) dose groups of L-oxiracetam were shown to reduce the number of learning errors in the jumping platform test for model mice; the high dose group also significantly prolonged the memory latency period in this passive avoidance test. All three dose groups notably decreased the number of memory errors within the first 3 minutes, showing a statistically significant difference compared to the model group (P < 0.05). The efficacy of the high dose group was comparable to the oxiracetam group (600 mg/ kg) and superior to the R-oxiracetam group (300 mg/ kg), although the difference was not statistically significant (P > 0.05).

In the dark avoidance test (passive avoidance), both the medium and high doses of L-oxiracetam significantly extended the memory latency period for the model mice. These doses also significantly reduced the number of errors compared to the model group (P < 0.05). In the Y-maze test (active avoidance), all dose groups of L-oxiracetam reduced the number of training attempts needed for the mice to escape to the correct arm, and increased the number of proactive escapes. The medium and high dose groups significantly improved accuracy compared to the model group (P < 0.05, P < 0.01). In the Morris water maze test, which assesses spatial learning and memory capabilities, the medium and high dose groups of L-oxiracetam significantly reduced the latency period of the location navigation trial (P < 0.05). All three dose groups significantly enhanced the number of original platform crossings in the spatial exploration test, showing marked differences compared to the model group (P < 0.05, P < 0.01). In these experiments, the efficacy of the high dose group was equivalent to that of the oxiracetam group and superior to the R-oxiracetam group (P < 0.05).

1.3.2 Main Toxicological Study Results

1.3.2.1 Acute Toxicity Test

The test results indicate that the minimum lethal dose (MLD) of injectable L-oxiracetam administered via a single intravenous injection in mice is greater than 5g/ kg. In beagle dogs, the maximum tolerated dose (MTD) of injectable L-oxiracetam given via a single intravenous infusion is also equal to or greater than 5 g/ kg.

1.3.2.2 Long-Term Toxicity Test

(1) Long-Term Toxicity Test: Three-Month Study in Rats

In this segment of the study, Sprague-Dawley rats were used to evaluate the long-term toxicity of injectable L-oxiracetam. The rats exhibited good tolerance to the drug at doses up to 800 mg/ kg, with no apparent toxic effects observed. At a dose of 1600 mg/ kg, a transient decrease was noted in the total platelet (PLT) count and plateletcrit (PCT) in female rats. At the same dosage levels, the toxicity of injectable L-oxiracetam was found to be similar to that of the control drug, injectable oxiracetam. Two weeks after cessation of dosing, no significant delayed toxic effects were observed in any of the dosage groups or the positive control group.

(2) Long-Term Toxicity Test: Three-Month Study in Beagle Dogs

The study evaluated the long-term toxicity of injectable L-oxiracetam in beagle dogs, demonstrating good tolerance at a dose of 300 mg/ kg with no evident toxic effects. At a dose of 600 mg/ kg, minor adverse reactions were observed, primarily manifesting as a slowed increase in body weight in female test dogs. At a higher dose of 1200 mg/ kg, more severe adverse reactions occurred, including significant gastrointestinal discomfort primarily characterized by reduced food intake. In comparison to the positive control group receiving injectable oxiracetam, the gastrointestinal adverse reactions such as vomiting or retching and loose stools were noticeably less severe with injectable L-oxiracetam. There were no drug-related pathological changes in the organs and tissues of any groups, including both the L-oxiracetam and oxiracetam groups, at equivalent dosage levels. Furthermore, no significant toxicological differences were noted between injectable L-oxiracetam and the control drug, injectable oxiracetam.

Four weeks after the cessation of dosing, no significant delayed toxic effects were observed in any of the test or control groups.

1.3.2.3 Special Safety Tests (Allergenicity, Hemolysis, Local Irritancy, etc.)

At a concentration of 40 mg/ ml, injectable L-oxiracetam showed no irritant effects on the vascular tissues of rabbit ears. In vitro hemolysis tests revealed no hemolytic activity, nor were there any signs of red blood cell (RBC) agglutination. In active systemic anaphylaxis tests conducted on guinea pigs at doses of 288 mg/ kg and 64 mg/ kg, injectable L-oxiracetam yielded negative results. Similarly, passive cutaneous anaphylaxis tests performed at doses of 300 mg/ kg and 60 mg/ kg also returned negative results for allergic reactions.

1.3.2.4 Mutagenicity Test

Injectable L-oxiracetam at doses ranging from 5 to 5000 μg/ plate, both with and without S9 mix, did not show a dose-dependent increase in the number of revertant colonies of Salmonella typhimurium histidine auxotroph strains TA97, TA98, TA100, TA102, and TA1535, indicating a negative result in the Ames test.

In the chromosome aberration test, Chinese hamster lung fibroblasts were exposed to injectable L-oxiracetam for 24 and 48 hours under non-activated conditions, and for 6 hours under metabolic activation conditions, followed by a drug-free period of 18 hours before cell harvesting. At drug concentrations of 250, 500, and 1000 ug/ mL, there was no dose-dependent increase in the rate of chromosomal aberrations, indicating a negative result in the chromosome aberration test.

ICR mice were administered injectable L-oxiracetam via tail vein injection at doses of 500, 1000, and 2000 mg/ kg once daily for three consecutive days. Bone marrow was collected 24 hours after the last dose to prepare slides. There was no observed increase in the number of polychromatic erythrocytes with micronuclei in the bone marrow at any of the three dose levels, indicating a negative result in the micronucleus test.

1.3.3 Non-Clinical Pharmacokinetic Study Results

1.3.3.1 Pharmacokinetic Parameters of L-Oxiracetam

After a single intravenous injection of L-oxiracetam at low (200 mg/ kg), medium (400 mg/ kg), and high (800 mg/ kg) doses in rats, the Area Under the Curve (AUC)_0-∞_ values for L-oxiracetam were 292, 634, and 1332 µg•h/ mL, respectively, showing a dose-proportional increase. The plasma clearance (CL) of L-oxiracetam was between 622 and 692 mL/ h/ kg, the estimated plasma elimination half-life (T_1/ 2_) ranged from 1.84 to 2.86 hours, and the steady-state volume of distribution (Vss) was between 619 and 674 mL/ kg.

In beagle dogs, after a single intravenous injection at low (60 mg/ kg), medium (120 mg/ kg), and high (240 mg/ kg) doses of L-oxiracetam, the AUC_0-∞_ values were 309, 725, and 1637 µg•h/ mL, respectively, also showing a dose-proportional increase. The CL of L-oxiracetam ranged from 147 to 198 mL/ h/ kg, the estimated T_1/ 2_ was between 5.42 and 7.64 hours, and the Vss was between 489 and 624 mL/ kg.

In both rats and beagle dogs, the main pharmacokinetic parameters of L-oxiracetam showed no significant differences based on sex across low, medium, and high doses.

1.3.3.2 Tissue Distribution of L-Oxiracetam

After administering a 200 mg/ kg dose of L-oxiracetam via intravenous injection to rats, the peak concentration in all tissues was reached within 0.5 hours. The concentration of L-oxiracetam was notably high in the kidneys, suggesting that the drug is likely excreted through the renal system. Except at certain time points, the concentrations of L-oxiracetam in the liver, lungs, spleen, and gastrointestinal tract were generally similar to those in the plasma. Concentrations in the heart, skeletal muscle, gonads, and adipose tissue were generally lower than in plasma. Traces of L-oxiracetam were detected in brain tissue, indicating that it can cross the blood-brain barrier. The clearance of L-oxiracetam from brain tissue was significantly slower than from plasma. Four hours post-administration, the concentration of L-oxiracetam in the whole brain was greater than half of the plasma concentration. This characteristic may be beneficial for its clinical efficacy in treating neurological conditions.

1.3.3.3 Chiral Conversion between L-Oxiracetam and R-Oxiracetam

After administering either L-oxiracetam or R-oxiracetam to rats and beagle dogs, no enantiomers were detected in the plasma of either species. This indicates that there is no significant chiral conversion between L-oxiracetam and R-oxiracetam in the bodies of rats and beagle dogs.

1.3.3.4 Comparative Study of the Pharmacokinetic Behavior and Tissue Distribution between L-Oxiracetam and Racemic Oxiracetam

When administered alone, the pharmacokinetic behavior of L-oxiracetam in the plasma and its tissue distribution in rats were essentially consistent with those observed when administered as part of racemic oxiracetam. In both rats and beagle dogs, the bioavailability of L-oxiracetam relative to when administered as part of racemic oxiracetam was 113.89% and 92.10%, respectively.

1.3.3.5 Plasma Protein Binding, Metabolic Pathways, and Excretion Routes of L-Oxiracetam

Direct studies on the plasma protein binding rate, metabolism, and excretion pathways of L-oxiracetam have not been conducted. Existing pharmacokinetic data for oxiracetam indicate that after oral administration of 10 mg/ kg of oxiracetam to fasting male rats, the plasma protein binding rates at 30 minutes, 2 hours, and 4 hours were 0%, 0%, and 2.8%, respectively. In vitro tests have shown that the plasma protein binding rate of oxiracetam at concentrations of 1-100μg/ ml with male rat plasma is between 1% and 2.5%, while the binding rate with canine and human plasma is 0%. After oral administration of oxiracetam to male rats, no metabolites were detected in the plasma, and approximately 99% of the administered dose was recovered unchanged in the urine and feces within 24 hours, with only 1% consisting of unknown metabolites. After oral administration of 10 mg/ kg oxiracetam to fasting male rats, 51.3% and 44.1% of the drug were recovered from urine and feces, respectively, within 24 hours. Intravenous administration of 10 mg/ kg oxiracetam to fasting male rats resulted in 96.5% and 1.6% of the drug being recovered from urine and feces, respectively, within 24 hours. Biliary cannulation tests show that only a very small amount of the drug is excreted through the bile after oral administration to fasting male rats, with about 0.4% of the administered dose being excreted via the bile within 48 hours. Given that no significant differences in pharmacokinetic parameters have been observed between L-oxiracetam and R-oxiracetam in rats and dogs, and there is no significant chiral conversion, it can be inferred that L-oxiracetam has a very low plasma protein binding rate in rats, dogs, and humans. After intravenous injection, L-oxiracetam is primarily excreted unchanged in the urine in rats.

1.3.4 Clinical Research Results

1.3.4.1 Phase I Clinical Human Tolerability Test

The Phase I clinical tolerability trial of injectable L-oxiracetam was conducted from April 2014 to March 2015 at Yijishan Hospital of Wannan Medical College. A total of 80 subjects were enrolled, undergoing a single-dose administration across seven dosage groups: two subjects in the 0.6g group, four in the 1.2g group, ten in the 2.0g group, six in the 3.0g group, ten in the 4.0g group, six in the 6.0g group, and ten in the 8.0g group, completing a total of 48 subjects. Additionally, a multi-dose administration was carried out in two dosage groups, with ten subjects each in the 4.0g and 8.0g groups; and a chiral pharmacokinetic dose group consisting of 12 individuals. Results showed that during the trial, one subject experienced a Grade 1 adverse event (AE): a subject in the 4.0g group (Subject No. 69) reported mild abdominal bloating and discomfort after lunch on the fourth day of continuous dosing, which resolved after a bowel movement. Previous clinical applications of racemic oxiracetam have reported adverse reactions such as nausea, vomiting, and stomach discomfort. Therefore, it is considered that the AE is "possibly related" to the test drug, likely being drug-related. The severity of the AE was mild, not affecting normal activities. Clinical observation was continued without intervention, and the condition resolved naturally. Conclusion: The trial demonstrated that L-oxiracetam is well-tolerated and safe in a dosage range from 0.6g to 8.0g, with repeated dosing.

1.3.4.2 Phase I Clinical Human Pharmacokinetic Test

The Phase I pharmacokinetic study of injectable L-oxiracetam was conducted from April 2014 to March 2015 at Yijishan Hospital of Wannan Medical College.

Single-Dose Study: A single-dose parallel trial design was employed, involving 30 healthy participants, evenly split between males and females. They were divided into three dosage groups for the pharmacokinetic study of injectable L-oxiracetam: 2g, 4g, and 8g, with each group comprising 10 individuals.

Multiple-Dose Study: A single-dose single-cycle trial design was used, involving 10 healthy participants, also evenly split between males and females. They received a daily intravenous drip of 4.0g of injectable L-oxiracetam for a consecutive 7 days.

Chiral Pharmacokinetic Comparative Study: This study utilized a randomized two-period crossover design. Twelve healthy participants, evenly split between males and females, were divided into two groups. In Group I, participants received an intravenous drip of 3.0g L-oxiracetam in the first period, followed by a 7-day washout period, after which they received a 6.0g dose of racemic oxiracetam in the second period. Conversely, Group II participants were administered 6.0g of racemic oxiracetam in the first period and 3.0g L-oxiracetam in the second period.

Results: Throughout the trial involving 52 participants, no serious adverse events (SAEs) occurred, and the tolerability was good.

For the injectable L-oxiracetam, the pharmacokinetic parameters such as Peak concentration (C_max_), AUC_0-t_, and AUC_0-∞_ increased with the dosage range from 2g to 8g. The T_1/ 2_ and Vz were found to be independent of the administered dose. Using Spearman rank correlation analysis, a linear relationship was identified between the dose and parameters C_max_, AUC_0-t_, and AUC_0-∞_.

After multiple dosing of 4.0g of injectable L-oxiracetam once daily, steady-state was achieved by the fifth day, with a trough concentration at steady-state of (3.22 ± 0.78) ug/ mL. The pharmacokinetic parameters on the 7th day of continuous dosing (AUC_0-t_, AUC_0-∞_, C_max_) showed no statistically significant difference (P > 0.05) compared to those after a single dose (day 1), suggesting no accumulation of L-oxiracetam with the once-daily dosing regimen as indicated by the accumulation ratio near 1. Similarly, no significant difference was observed in the parameters t1/ 2z and Vz after 7 days of dosing compared to the first day (P > 0.05), indicating that the elimination characteristics of L-oxiracetam in humans are not altered by continuous dosing for 7 days.

Significant statistical differences between genders were observed in the 2g dosage group for C_max_, AUC_0-t_, AUC_0-∞_, and CLz; the Vz on the first day in the 4.0g multiple dosing group; and the steady-state AUC (AUCsst), AUC_0-∞_, and CLz on the 7th day of the 4.0g multiple dosing group (P < 0.05). The pharmacokinetic differences between genders for other dosage groups were negligible.

In the 4.0g group, urine and fecal samples were also collected. Approximately 60% of injectable L-oxiracetam was excreted unchanged in urine, while a very small amount, less than 1%, was excreted unchanged in feces.

In a comparative study between 3.0g L-oxiracetam and 6.0g racemic oxiracetam administered intravenously to 12 subjects, pharmacokinetic parameters for s-ORT were similar. Using bioequivalence testing methods, the AUC_0-t_, AUC_0-∞_, and C_max_ data for L-oxiracetam were log-transformed, analyzed using variance analysis and two-sided t-tests, and the 90% confidence interval (CL) were calculated. The results indicated that the geometric mean ratios of AUC_0-t_, AUC_0-∞_, and C_max_ were within the bioequivalence range, specifically 89.7%–100.0%, 89.5%–99.8%, and 85.0%–105.0%, respectively.

Phase I pharmacokinetic data suggest that the pharmacokinetic behaviors of L-oxiracetam and racemic oxiracetam in humans are fundamentally consistent. Therefore, injectable L-oxiracetam can adopt a once-daily dosing regimen consistent with that of racemic oxiracetam for subsequent clinical studies.

1.3.4.3 Phase II Clinical Trials for Efficacy and Safety Evaluation

The phase II exploratory clinical trial adopted a parallel control design with groups receiving injectable L-oxiracetam (3g/ day, 4g/ day), a positive control group receiving oxiracetam, and a placebo group, each conducted in a 1:1:1:1 ratio for efficacy and safety exploration, totaling 200 participating subjects. Preliminary statistical analysis of the phase II clinical study using the Per Protocol Set (PPS) indicated that three months post-treatment, the changes in the Loewenstein Occupational Therapy Cognitive Assessment (LOTCA) scale total scores from baseline were as follows: high dose group (4g/ day) 26.0 ± 11.8, low dose group (3g/ day) 22.2 ± 10.3, positive control group 22.8 ± 10.0, and placebo group 20.0 ± 11.4. The high dose group (4g/ day) showed statistically significant improvements compared to the placebo group (P < 0.05). The low dose group (3g/ day) did not show a statistically significant difference compared to the placebo group (P > 0.05), but there was a trend of improvement. Phase II safety results indicated that no SAEs related to the test drugs occurred, demonstrating good overall tolerability and safety.

The Phase III clinical trial is planned to be conducted with participants allocated to the high-dose treatment group (4g/ day), a positive control group, and a placebo group in a 2:2:1 ratio.

**1.4 Potential Risks and Benefits**

Since L-oxiracetam has not been marketed domestically or internationally, its clinical risks and benefits are assessed with reference to those of oxiracetam.

1.4.1 Known Potential Risks

Oxiracetam generally has few adverse reactions. However, it can cause anxiety, skin itching, rash, nausea, and stomach pain, which usually resolve after discontinuation of the drug. A reduction in dosage may be necessary for a small number of patients who experience psychiatric excitement or sleep abnormalities. Caution is advised in patients with allergies to this drug or with renal impairment; a reduced dosage should be used if the drug is necessary.

1.4.1.1 International Research Results

Son et al. conducted a human tolerability study confirming that the administration of oxiracetam at doses ranging from 400 to 2000 mg per day did not result in any adverse reactions.^16^ There were no changes in heart rate, blood pressure, respiration, electrocardiogram (ECG), electroencephalogram (EEG), liver, or kidney functions, indicating that oxiracetam is a drug with extremely low toxicity.

Rozzini and colleagues randomly divided 96 patients with cognitive impairments into two groups for a 26-week treatment with either oxiracetam (1600 mg) or a placebo.^17^ The group treated with oxiracetam demonstrated good compliance, and the study confirmed that oxiracetam is well-tolerated at this dosage.

In a one-year study conducted by Villardita et al., involving 60 patients with mild to moderate dementia treated with oxiracetam, no serious adverse reactions were observed.^18^ They concluded that a daily dose of 1600 mg of oxiracetam is safe.

1.4.1.2 Domestic Research Results

Yu and colleagues conducted a human tolerability study on oxiracetam using a starting dose of 200 mg, which is one-tenth of the most common adult dose used internationally (2000 mg/ day), with increments up to the standard international dose of 2000 mg.^19^ The trial included six dosage groups: 400, 800, 1200, and 1600 mg, in addition to the starting and maximum doses. The tolerability trial was carried out in an ascending dose order, with each participant receiving only one dose level. Measurements taken before and after medication included vital signs, neurological function, adverse reactions, and laboratory tests. The results were analyzed using a t-test to compare pre- and post-medication changes. Among the 22 participants who received varying doses, particularly a single oral dose of 2000 mg of oxiracetam, no adverse reactions were observed, and all subjects tolerated the drug well. Besides a significant decrease in triglyceride (TG) levels at doses of 800, 1200, 1600, and 2000 mg (P < 0.05), no significant statistical differences were noted in other measured parameters (P > 0.05).

In a study conducted by He and colleagues on the efficacy of oxiracetam injection in treating Alzheimer's disease, the treatment group received oxiracetam injection added to 5% glucose or saline solution for intravenous infusion.^20^ Each treatment cycle lasted 21 days, followed by a 7-day drug holiday before the next cycle. The control group was administered Cytidine Diphosphate Choline (CDP-Choline) 0.6g added to 5% glucose or saline solution, with the same treatment duration as the therapy group. After three treatment cycles, the effectiveness and safety were evaluated. In the CDP-Choline group, three cases experienced nausea, dizziness, headache, and insomnia, while in the oxiracetam group, two cases reported nausea, vomiting, and stomach discomfort, which improved after symptomatic treatment.

During a clinical observation conducted by Zhao and colleagues involving 327 patients, no adverse reactions were reported.^21^

1.4.2 Known Potential Benefits

Cranial brain injuries are common in neurosurgery and have the highest rates of mortality and disability among all types of trauma. Timely treatment during the acute phase is crucial for reducing both mortality and disability rates. Cognitive impairment and memory decline are the most common sequelae following brain trauma. Early EEG changes characteristic of brain trauma primarily include an increase in δ and θ slow waves, and a reduction in α and β wave power; the proliferation of δ and θ slow waves is often diffuse, which correlates with brain dysfunction after the injury. The power of the slow waves is related to the severity of the injury; more severe brain injuries are associated with higher and more pronounced θ power.

1.4.2.1 International Related Clinical Research

Russello D and colleagues conducted a study with 36 patients who were suffering from post-concussion syndrome, randomly dividing them into two groups.^22^ One group received treatment with oxiracetam. The results indicated that oxiracetam significantly improved the symptoms of patients with post-concussion syndrome. It enhanced the amount of α activity in the EEGs of patients with dementia and reduced δ activity, with the most notable effects observed in the frontal-temporal regions.

1.4.2.2 Domestic Related Clinical Research

Dan and colleagues investigated the effects of oxiracetam on brain function in patients with acute mild brain injuries.^23^ They selected 48 patients who were admitted within 24 hours after injury, all classified with mild brain injuries (Glasgow Coma Scale (GCS) scores between 12 and 15). The study was randomized and double-blind, comparing the effects of oxiracetam with those of piracetam. Changes in brain wave activity were monitored using quantitative EEGs before and after medication, focusing on changes in relative power values and ratios. After medication, changes in EEGs showed that in the oxiracetam group, all patients' indices increased, wave amplitudes rose, and generalized activity decreased. In contrast, only 21 patients in the piracetam group showed these changes, and 3 patients showed insignificant changes; focal activities were not completely eliminated in all cases. The study demonstrated that oxiracetam significantly improves brain function in patients with acute mild brain injuries, showing a stronger effect than piracetam.

Shu and colleagues conducted a randomized double-blind trial involving 120 patients with moderate TBIs, comparing the effects of oxiracetam to the positive control drug piracetam (sold under the brand name Cerebrolysin).^24^ The treatment group received oral oxiracetam capsules at a dose of 2.4g per day, with each treatment cycle lasting 30 days. The efficacy of the treatment was evaluated using several scales: the Mini-Mental State Examination (MMSE), GCS, Glasgow Outcome Scale (GOS), and self-reported symptom scores. The results indicated that among patients treated with oxiracetam, the marked effectiveness rate was 33.3%, the effectiveness rate was 50.0%, and the total effectiveness rate was 83.3%. Oxiracetam demonstrated significant superiority in improving patients' quality of life, cognitive abilities, and memory functions compared to piracetam. These differences were statistically significant (P < 0.05).

Wu and colleagues assessed the clinical efficacy of oxiracetam in the treatment of acute cranial brain injuries.^25^ They enrolled 76 patients who met the study criteria and randomly divided them into two groups: an oxiracetam treatment group with 38 patients and a control group receiving standard treatment, also with 38 patients. The study observed changes in the GCS scores during different periods of the treatment and the GOS scores at the end of the treatment and during the recovery period. The results indicated that the GCS scores of the oxiracetam treatment group were significantly better than those of the control group after 7 days of medication (P < 0.05). GOS scores assessed at the end of the 2-week treatment period and again 4 months later during follow-up also showed that the treatment group had significantly better outcomes than the control group (P < 0.05). Mortality rates were evaluated at the end of the follow-up period, with 5 deaths (10.4%) in the treatment group compared to 12 deaths (25.0%) in the control group, demonstrating a statistically significant difference (P < 0.05). The study concluded that early use of oxiracetam in the treatment of acute cranial brain injuries is effective, has few adverse reactions, significantly reduces mortality rates, and improves prognosis.

**2 Trial Objectives**

- Primary objectives
- To evaluate the efficacy of L-oxiracetam on functional outcome in patients with TBI.
- To evaluate the safety of L-oxiracetam in patients with TBI.
- Secondary objectives
- To analyse changes in function and disease severity of patients with TBI treated with either L-oxiracetam, oxiracetam or placebo.
- Exploratory objectives
- To evaluate influence in subgroup (such as age, gender and so on) in patients with TBI treated with either L-oxiracetam, oxiracetam or placebo.

**3 Trial Design**

**3.1 Summary of Trial Design**

This multicenter study is a randomized, double-blind, parallel, three-arm, phase III clinical trial. The design and implementation of this trial strictly adhere to the Helsinki Declaration and have obtained approval from the ethics committees of each research center. This trial was approved by the China National Medical Products Administration (Approval Number, 2016L03521) and registered on chinadrugtrials.org.cn (identifier, CTR20192539) and ClinicalTrials.gov (identifier, NCT04205565). The rights and safety of the subjects in this trial are prioritized over the benefits to science and society.

The trial is planned to be conducted in 74 centers across 51 hospitals in China (see **Appendix 1**), and subjects will not be enrolled in the study until written informed consent (see **Appendix 2**) is obtained from the subjects and/ or their guardians/ legal representatives. All qualified medical centers undergo rigorous training before the start of the study and adhere to standardized treatment for enrolled patients.

Eligible TBI patients will be randomly assigned to three groups: L-oxiracetam group, oxiracetam group, and placebo group in a ratio of 2:2:1. The efficacy and safety of improving memory and cognitive impairment in TBI patients will be observed over a treatment period of 14 days, with a follow-up period of 90 days.

**3.2 Trial Period**

September 2019- May 2024.

**4 Target Population**

**4.1 Eligibility of Target Population**

Patients are aged 18 to 75 years and have mild to moderate (GCS score of 10 to 15) acute TBI.

And investigators and sponsor should discuss with sharing sufficient information regarding the eligibility of patients before the enrollment.

**4.2 Inclusion and exclusion criteria**

4.2.1 Inclusion criteria

- Age 18-75 years;
- Head injury meeting all of the following conditions: 1) Clear evidence of head trauma in the current diagnosis, including closed head injury or head injury with cerebrospinal fluid leakage and/ or ear or nasal leakage and/ or intracranial air accumulation; 2) Confirmed by magnetic resonance imaging (MRI) or computed tomography (CT) to have intracranial bleeding above the cerebellar tentorium (including cerebral contusion, subarachnoid hemorrhage, extradural hematoma, subdural hematoma, intracerebral hematoma, etc.), with or without transient loss of consciousness; 3) Classified as mild to moderate head injury (GCS: 10-15); 4) Stable condition within 72 hours after head injury, undergoing conservative treatment, not undergoing craniotomy (may have intracranial pressure monitoring without general anesthesia or basal anesthesia);
- MMSE score below normal, with diagnostic cutoff values depending on different educational levels: illiterate (no education) ≤ 19 points, elementary school level ≤ 22 points, junior high school and above level ≤ 26 points;
- Consent from the guardian and/ or patient to participate in this clinical trial and signing of the informed consent form.

4.2.2 Exclusion criteria

- Known or suspected allergy to the experimental drug or its components;
- Use of prohibited drugs or other cognitive-enhancing drugs listed in the protocol after injury;
- History of severe head trauma, cerebrovascular accidents, or structural brain lesions;
- Conditions such as speech/ hearing impairment that prevent completion of cognitive function assessment;
- Occurrence of secondary brain injury after the current head injury;
- Need for craniotomy or external ventricular drainage;
- Concurrent serious injuries to other major organs or serious complications that may affect the subject's life;
- Patients with active epilepsy within the past year;
- Severe liver or kidney disease with abnormal liver or kidney function tests (alanine aminotransferase (ALT), aspartate aminotransferase (AST)) ≥ 3 times the upper limit of normal, serum creatinine (Scr) > upper limit of normal);
- Concurrent severe heart disease, lung disease, blood and hematopoietic system diseases, gastrointestinal diseases, or other severe or progressive systemic diseases;
- History or current diagnosis of malignant tumors (excluding cured stage IB or lower cervical cancer, non-invasive basal cell or squamous cell skin cancer; exclusion of breast cancer with complete remission (CR) > 10 years, malignant melanoma with CR > 10 years, and other malignant tumors with CR > 5 years);
- Presence of neurological or psychiatric disorders that prevent or unwillingness to cooperate;
- Pregnant, lactating women, or those with recent plans for childbirth;
- Investigator deems unsuitable for participation in the clinical trial;
- Participation in another clinical trial and use of experimental drugs in the last 3 months before the trial.

**4.3 Case criteria for discontinuation/ withdrawal**

4.3.1 Termination/ Withdrawal Decided by the Investigator

- During the clinical trial, the subject experiences other comorbidities, complications, or special physiological changes that make it inappropriate to continue the trial.
- The occurrence of SAEs or significant AEs makes it inappropriate for the subject to continue in the trial.
- The subject exhibits poor compliance, such as not taking the medication after enrollment, having no follow-up records, changing medication on their own, or using treatment drugs not specified in the protocol.
- The subject was mistakenly included in the trial despite not meeting the inclusion criteria.
- The subject meets any of the exclusion criteria.

4.3.2 Withdrawal Initiated by the Subject

- The subject, unwilling or unable to continue with the clinical trial, requests withdrawal from the researcher. Various reasons for withdrawal include perceived lack of therapeutic effect, intolerance to certain adverse reactions, economic factors, or unspecified reasons. Efforts should be made to understand the reasons for withdrawal and document them accordingly.
- The subject does not explicitly request withdrawal but discontinues medication and testing, resulting in loss to follow-up.

4.3.3 Handling of Cases of Termination/ Withdrawal

For cases that terminate/ withdraw from the trial prematurely, researchers should take proactive measures to complete the last assessment as far as possible, to facilitate analysis of efficacy and safety. All cases of termination/ withdrawal should be documented in the electronic Case Report Form (eCRF), including a summary of the trial's conclusion and reasons for termination/ withdrawal.

**4.4 Criteria for Stopping the Trial**

Stopping a trial refers to discontinuing all trial activities before the planned end of the trial, primarily to protect the rights of the participants, ensure the quality of the trial, and avoid unnecessary financial losses. The criteria for stopping a trial include:

- If serious safety issues arise, the trial should be stopped promptly.
- If the drug is found to lack clinical value, the trial should be stopped to prevent delaying effective treatment for participants and to avoid unnecessary financial losses.
- Major errors in the clinical trial protocol are discovered, making it difficult to evaluate the drug's effects; or a well-designed protocol experiences significant deviations in implementation, complicating the assessment of the drug's effects.
- The sponsor requests termination (due to reasons like funding or management issues).
- The national regulatory authority mandates the trial's termination for any reason.

**5 Trial Procedures and Assessment Methods**

**5.1 Assessment Procedures**

The trial schedule is shown in “Trial Schedule”.

**5.2 Drug Provision**

- L-oxiracetam: Injectable L-oxiracetam, specification: 1g per vial, manufactured by Shenghe (China) Biopharma Co., Ltd.
- Oxiracetam: Injectable oxiracetam, specification: 1g per vial, manufactured by Shiyao Group Ouyi Pharmaceutical Co., Ltd.
- Placebo: Injectable L-oxiracetam or oxiracetam placebo with identical color, smell, and appearance but without active ingredients, manufactured by Shenghe (China) Biopharma Co., Ltd.

All investigational drugs will be provided by Nanjing Yoko Pharmaceutical Co., Ltd. in accordance with blinding requirements and meet quality standards.

**5.3 Drug Packaging and Labeling**

All study medications are assigned a specific drug number and are labeled accordingly. The external packaging of both the trial medication and the control medication is kept uniform. Each bulk package contains medication for 14 days plus an additional day.

Each package must be affixed with a label bearing the drug number. The label includes the approval number, drug number, drug name, indications, administration method, storage conditions, and the supplying entity of the medication, and is clearly marked with the phrase "For Clinical Research Use Only".

**5.4 Drug Storage and Stability**

Storage Conditions: Store in a cool (not exceeding 20°C), dry place, protected from light and sealed.

**5.5 Preparation of Formulations**

The trial medication is added by the research nurse to 100-250ml of 5% dextrose solution or 0.9% saline solution, shaken well, and administered intravenously at a standard rate without any special administration methods or rate requirements.

**5.6 Trial Period**

Screening period (-72h to 0h) + Treatment period (14+1 days) + Follow-up period of 90 Days.

**5.7 Trial Drug Accountability Procedure**

- All trial drugs are uniformly provided by the sponsor and transported to each clinical trial site according to the required storage conditions.
- Each clinical trial site appoints a dedicated person to manage the trial drugs (drug administrator). Upon receiving the drugs, the drug administrator should verify the following items before signing for them:
- Drug inspection report; ensure that the name, dosage form, specifications, batch number, and other information match the actual drug.
- Check whether the conditions during transportation conform to the storage requirements.
- Verify that the accompanying thermometer does not show any exceedance of limits. If it does, the sponsor must be notified immediately.
- Drug packaging: Inspect the outer packaging of the drugs to ensure it is intact and clearly labeled.
- Labels: Small bottles and large boxes must be labeled.
- The drug administrator will store the trial drugs separately and centrally according to their storage conditions. Daily temperature and humidity records must be kept during storage, and timely adjustments should be made when conditions exceed the specified ranges to ensure compliance with storage requirements.
- The drug administrator issues drugs to the research nurse based on prescriptions or orders from the research doctor, using drug codes, and accurately completes the drug dispensing/ collection log.
- After receiving the drugs, the research nurse stores them according to the trial drug storage conditions and prepares the drugs daily for use by the participants, without disclosing any specifics about the drugs to researchers or participants under any circumstances.
- The research nurse must hand over empty vials of injectables, any remaining drugs after use, and empty packaging directly to the drug administrator, and record this in the drug dispensing/ collection log.
- After the trial ends, the sponsor collects the remaining trial drugs (including those leftover by participants and those unused) for destruction by the sponsor itself.

**5.8 Trial Procedure Related Assessments**

- Demographic data: Includes age, gender, and ethnicity.
- Medical history: Inquiry or collection from existing medical records about the current condition of cranial injuries and any other diseases the participant might have, including medication and allergy history.
- Medication history: Inquire about prescription and over-the-counter drugs used prior to the administration of the trial drug, recording the generic name of the drugs, single dose administered, frequency, method of administration, and reasons for use.
- Disease diagnosis: Cranial injuries confirmed by CT or MRI scans (participants who have already been examined post-injury do not need to repeat the scans).
- Vital signs: Include body temperature, respiration, pulse, and blood pressure.
- Biological sample collection and laboratory assessment: Collection of blood and urine samples for routine blood tests, blood biochemistry, coagulation functions, liver and kidney function tests, TG, creatine kinase (CK) measurements, blood pregnancy tests, and routine urinalysis. Biological samples are to be collected, processed, and tested by the laboratories of the participating units. Before the receipt of trial drugs, clinical trial units must provide the sponsor with the normal reference ranges for each laboratory indicator and promptly inform the sponsor of any changes during the trial.
- 12-lead ECG: Heart rate and QT/ QTc interval results must be reported.
- Scale assessment: Use of the GCS to evaluate the severity of cranial injuries in participants, the MMSE, Montreal Cognitive Assessment (MoCA), and LOTCA to evaluate cognitive functions, the Glasgow Outcome Scale-Extended (GOS-E) to assess prognosis, and the Activities of Daily Life-the Barthel Index scale (ADL-BI) to evaluate improvements in post-injury living abilities of the participants. Detailed assessment scales and operating instructions are available in **Appendix 3**.

**5.9 Scale evaluation**

- Each research center must have at least one neurologist with professional experience in scale assessment to serve as a scale evaluator.
- Prior to the start of the trial, scale evaluators from each research center undergo training on the operation of scales. They must master the assessment methods of all scales used and pass a test on these methods before being issued a certificate of training completion.
- During their authorization period, scale evaluators are responsible solely for the assessment of participants using various scales and must not participate in any other research processes within the clinical trial.

**5.10 Clinical Laboratory Evaluation**

The laboratory evaluations include tests for routine blood work, blood biochemistry, coagulation functions, liver and kidney function, TG, CK, and urine analysis. The indicators to be tested are as follows:

- The laboratory test results obtained within 72 hours post-injury will be used as the entry criteria if multiple tests are conducted; the most recent test results closest to the end of medication use will be used for evaluation.
- Routine Blood Test: RBC, White Blood Cells (WBC), PLT count, and Hemoglobin (HGB) measurement.
- Coagulation Function: Prothrombin Time (PT), Activated Partial Thromboplastin Time (APTT), Thrombin Time (TT), and Fibrinogen (FIB).
- Blood Biochemistry: AST, ALT, Total Bilirubin (TBIL), Gamma-Glutamyl Transferase (γ-GT), Alkaline Phosphatase (ALP), Scr, Glomerular Filtration Rate (GFR), CK, and TG.
- The GFR is a derived indicator, calculated using the simplified MDRD：GFR [ml/ (min·1.73 m^2^)] = 186 × (Scr)^−1.154^ ×(age)^−0.203^ × (0.742 if female); When applying this formula, Scr must be measured in mg/ dl.
- Urine Test: Urine erythrocyte (ERY), Urinary leukocyte (LEU), Protein (PRO), and Glucose (GLU).
- Blood Pregnancy Test: All female participants of childbearing potential must undergo a blood pregnancy test.

These assessments ensure a comprehensive evaluation of the participants' health and safety during the trial, while also maintaining the integrity and reliability of the data collected.

**5.11 Screening Period (-72h to 0h)**

During this visitation window, the following tasks need to be completed and documented:

- Obtain written informed consent from the guardian and/ or participant.
- Clinical trial sites to assign screening numbers based on the order in which participant consent forms are signed.
- Researchers collect demographic data, medical history, and medication history, and perform vital signs checks on participants.
- Participants who have not undergone a CT or MRI scan post-injury should have one conducted.
- Collect blood and urine samples from participants to test for routine blood parameters (WBC, RBC, HGB, PLT), routine urine parameters (PRO, GLU, LEU, ERY), liver function (AST, ALT, TBIL, γ-GT, ALP), kidney function (Scr, GFR), coagulation function (PT, APTT, TT, FIB), CK, and TG. Conduct a 12-lead ECG and perform a blood pregnancy test in women of childbearing age. If these tests have been conducted post-injury already, they do not need to be repeated.
- Participants to complete the MMSE and the GCS for scale evaluation.
- Researchers to determine the suitability of participants for the trial based on collected information and test results, in accordance with inclusion and exclusion criteria.
- Participants to complete the MoCA and LOTCA for scale evaluation.
- Randomize participants into groups, assign drug numbers, and dispense treatment drugs.

**5.12 Treatment Period (14 days + 1 day)**

Prescriptions or medical orders are issued by research physicians based on randomly assigned drug numbers. Drug administrators dispense medications to research nurses based on these prescriptions or medical orders. Upon retrieval of the medication, the research nurse administers the prescribed dosages to the participants on a daily basis, with the regimen spanning a continuous period of 14 days. Within the 1-day window following the end of this administration period, the following tasks must be completed and recorded:

- Conduct vital signs checks.
- Researchers document changes in condition, AEs, concurrent illnesses, and concomitant medication use during the medication period.
- Participants to complete the MMSE, GCS, MoCA, LOTCA, GOS-E, and the ADL-BI for evaluation.

**5.13 Follow-Up Visits**

30 days Follow-Up (± 3 days after medication ends):

- Researchers conduct a telephone follow-up to assess the participant's ADL-BI. The evaluation is obtained by interviewing the participant or their guardian.

60 days Follow-Up (± 3 days after medication ends):

- Similar to the 1-month follow-up, researchers use a telephone interview to assess the participant's ADL-BI by speaking with the participant or their guardian.

90 days Follow-Up (within ± 3 days window after medication ends); Participants are required to visit the clinic, where the following procedures are carried out and documented:

- Vital signs are checked.
- CT or MRI scans are performed; these should be consistent with those done during the screening period.
- Collection of blood and urine samples to measure routine blood values (WBC, RBC, HGB, PLT), routine urine values (PRO, GLU, LEU, ERY), liver function (AST, ALT, TBIL, γ-GT, ALP), kidney function (Scr, GFR), coagulation function (PT, APTT, TT, FIB), CK, and TG. A 12-lead ECG is performed, and blood pregnancy tests are conducted for women of childbearing age.
- Participants complete cognitive and functional assessments including the MMSE, MoCA, LOTCA, GOS-E, and the ADL-BI.

**5.14 Unscheduled Visits**

During the trial, if a participant experiences any discomfort or adverse effects, an unscheduled visit should be arranged. This visit will allow for immediate assessment and management of any issues that arise, ensuring the participant's safety and well-being throughout the trial process.

These follow-up and unscheduled visits are crucial for monitoring the long-term effects and efficacy of the treatment, as well as ensuring the ongoing health and safety of participants.

**6 Concomitant Medications and Treatments**

**6.1 Basic Treatment**

Post-injury, standard treatments to control intracranial pressure and prevent complications from cranial injuries are permitted. However, it is mandatory to record in the eCRF details such as the generic names of medications or other therapies used, dosage, reasons for use, frequency, and timing. This data will be analyzed and included in the final report.

**6.2 Prohibited Medications and Treatments**

Post-injury cognitive rehabilitation treatments are not allowed. During the medication and follow-up periods, the use of drugs that might affect the efficacy evaluation is prohibited, including:

- Cholinesterase inhibitors such as donepezil, rivastigmine, galantamine, tacrine, and huperzine A.
- γ-lactam brain function enhancers, such as aniracetam, piracetam, oxiracetam, and other commercially available forms.
- Other medications such as nimodipine, vinpocetine, ginkgo biloba extracts, cerebrolysin, amantadine/ robenidine, gangliosides, citicoline, idebenone, amantadine, glutamic acid, erythropoietin injection, dihydroergotoxine, brain protein hydrolysates, murine nerve growth factor, pyritinol, acetylglutamine, piracetam, ethionamide, and hormone replacement therapy (growth hormones, estrogens, thyroid hormones).

**6.3 Permitted Medications and Treatments**

Symptomatic treatment of other comorbid diseases is allowed, provided it does not affect the evaluation of efficacy and safety. Such treatments must be documented in the eCRF, including generic names or names of therapies, dosage, reasons for use, frequency, and timing, to facilitate analysis and reporting.

Any medications used to treat AEs or for prophylactic purposes during the trial must be meticulously recorded in the eCRF.

**7 Endpoints evaluation criteria**

**7.1 Efficacy** **Endpoints:**

7.1.1 Primary endpoint

- The primary outcome measure is the change in scores on the LOTCA at 90 days post-treatment compared to baseline.

7.1.2 Secondary endpoints

The secondary outcome measures include:

- Changes in scores on the LOTCA and GCS at the end of treatment compared to baseline;
- Changes in scores on the MMSE and MoCA at the end of treatment and 90 days post-treatment compared to baseline;
- The percentage of subjects at each level of the GOS-E at the end of treatment and 90 days post-treatment;
- ADL-BI at the end of treatment, 30 days, 60 days, and 90 days.

**7.2 Safety Endpoints:**

- Vital signs (temperature, pulse, respiration, blood pressure);
- Laboratory tests: Complete blood count (WBC, RBC, HGB, PLT); Urinalysis (PRO, GLU, LEU, ERY); Liver function tests (AST, ALT, TBIL, γ-GT, ALP); Renal function tests (Scr, GFR); CK; TG; Coagulation profile (PT, APTT, TT, FIB);
- 12-lead ECG;
- AEs.

**8 Safety assessment**

**8.1 Definitions**

- AE: An AE is any unfavourable medical occurrence in a participant that may present as symptoms, signs, illness, or laboratory findings after taking the trial medication. These events are not necessarily causally related to the trial medication.
- SAE: A SAE is any adverse medical event that occurs after taking the trial medication and results in death, life-threatening situations, permanent or significant disability or incapacity, requires hospitalization or prolongation of existing hospital stay, or causes congenital anomalies or birth defects.
- Suspected Unexpected Serious Adverse Reaction (SUSAR): This refers to an adverse reaction that is both unexpected and serious, where the nature and severity of the clinical presentation exceed what is described in the drug's Investigator's Brochure, the marketed product's package insert, or the product characteristics summary, and where the reaction is not anticipated based on the current knowledge.
- Drug Adverse Reaction: This term refers to any harmful or undesired effect in clinical trials that is possibly related to the trial medication. There is at least a reasonable possibility of a causal relationship between the trial medication and the AE, meaning that the relationship cannot be ruled out.

**8.2 Safety Information Related to the Trial Medication**

The trial drug, L-oxiracetam for injection, has been associated with clinical adverse reactions including anxiety, skin itching, rash, nausea, and stomach pain, which typically resolve after discontinuation of the drug. A few patients have reported psychiatric excitation and sleep disturbances.

The control drug, oxiracetam for injection, occasionally causes skin itching, nausea, psychiatric excitation, and sleep disturbances.

**8.3 Management and Documentation of** **Adverse Events**

8.3.1 Management of Adverse Events

- Inpatients

If an AE occurs during a subject's hospital stay, it should be handled according to the following procedure:

Upon detecting an AE in a subject, the attending or on-duty doctor should promptly inform the researcher. If necessary, symptomatic treatment may be initiated first. The research physician should preliminarily assess the severity and relation to the investigational drug and provide further handling advice:

- - General AE: Close monitoring of the event's outcome or corresponding symptomatic treatment may be conducted;
  - Significant AE: The research physician should immediately inform the principal investigator. Based on the subject's condition, treatment may be temporarily suspended, medication dosage adjusted, or targeted treatment measures taken. If necessary, the principal investigator may decide whether to urgently unblind (if applicable);
  - SAE: Handled and reported according to the following "8.7".
  - The research physician should treat according to the medical condition, and if the subject's injury exceeds the treatment capabilities of the research department, consultation and assistance from relevant departments should be sought.
- Outpatients

Upon learning of an AE in a subject, the research physician should thoroughly inquire about the subject's symptoms, signs, and location at the time of the event and provide necessary explanations and guidance. An initial assessment of the severity and relevance of the AE should be made. If the subject is at a local medical institution, the physician should contact the attending doctor by phone to re-assess the severity of the AE and provide handling advice:

- General AE: Initial treatment at a local hospital may be advised, with a notification to closely monitor the outcome of the event;
- Significant AE: It is suggested that the subject return to the hospital for treatment or seek treatment at a local hospital, and promptly inform the principal investigator. If the local hospital's facilities are limited, a doctor should be sent for treatment. If necessary, log in to the Interactive Web Response System (IWRS) to initiate the unblinding procedure. Treatment suspension, medication dosage adjustment, and symptomatic treatment should be adopted as per the protocol requirements.

8.3.2 Recording of AEs

- The research physician should make detailed records of AE, which must include: the name of the AE, start and end times, severity, measures taken, relation to the research drug, and outcome.
- Detailed records of the occurrence, progression, and treatment of SAEs should be made in the original medical records as much as possible and entered into the eCRF forms. This SAE should be tracked until it is resolved properly, the condition stabilizes, or the cause is clearly identified.

**8.4 Severity grading of AEs**

Mild: Minor self-perceived symptoms that are tolerable, do not affect daily activities, are transient, resolve on their own during continued medication use, and require no treatment.

Moderate: Symptoms are noticeable, affect the subject's daily activities, persist for a longer duration, and may resolve on their own or after symptomatic treatment. They may interfere with the use of the study medication, possibly necessitating a reduction in dosage or discontinuation of the drug.

Severe: The subject’s bodily functions are impaired, losing the ability to work or live normally, with symptoms persisting for a long duration, requiring discontinuation of the medication and appropriate management before resolution.

**8.5 Assessment of the Relationship between AEs and the Investigational Drug**

Based on whether there is a reasonable temporal sequence between the occurrence of AE and the use of the study drug, the type of drug reaction, and whether the reaction lessens, disappears, or reappears after discontinuation of the drug, the association between the AEs and the study drug is classified as definitely related, probably related, possibly related, possibly unrelated, and definitely unrelated. The first three categories are considered possibly related to the study drug, and evaluated as adverse drug reactions.

Details of the relationship between AE and the study drug are as follows:

- Definitely related: There is evidence of the use of the study drug, and the occurrence of the AE has a reasonable temporal sequence with the use of the study drug. The AE is more reasonably explained by the study drug than by other causes. Positive reaction upon discontinuation, positive reaction upon re-administration (if feasible).
- Probably related: There is evidence of the use of the study drug, and the occurrence of the AE has a reasonable temporal sequence with the use of the study drug. The AE is more reasonably explained by the study drug than by other causes. Positive reaction upon discontinuation.
- Possibly related: There is evidence of the use of the study drug, and the occurrence of the AE has a reasonable temporal relation with the use of the study drug. The AE can also be explained by other causes. Positive reaction upon discontinuation.
- Possibly unrelated: There is evidence of the use of the study drug, but the AE is more reasonably explained by other causes. Negative or unclear reaction upon discontinuation.
- Definitely unrelated: The study drug was not used, or there is no temporal relationship between the use of the study drug and the occurrence of the AE, or there is a clear other cause of the AE.

**8.6 Follow-up of Adverse Event Outcomes**

It is necessary to follow up on AEs that have not resolved by the end of the study or are still unresolved, until one of the following occurs:

- The event is alleviated;
- The event stabilizes;
- If baseline values are acceptable, the event returns to baseline levels;
- When no further information can be obtained (e.g., if the subject or their healthcare provider refuses to provide further information, or if a subject is proven to be lost to follow-up despite efforts).

Follow-up methods can vary based on the severity of the adverse reaction, including hospitalization, outpatient visits, home visits, phone calls, and correspondence.

**8.7 Serious AEs**

8.7.1 Management of SAEs

- When considered a SAE, the attending physician must notify the principal investigator or other responsible physician to attend. If the condition is critical, resuscitation should begin while notifying the project leader; if necessary, the investigational drug should be immediately discontinued;
- If identified as a SAE, appropriate clinical emergency treatment measures should be taken immediately based on clinical symptoms. If caused by a drug overdose leading to severe toxicity, the researcher should decide on measures to accelerate drug excretion to stabilize vital signs as much as possible, with cardiac monitoring if necessary, and consult with relevant departments for assistance if needed.
- If an outpatient is judged to have a SAE and cannot come to the clinic, it is advised that the subject return to the hospital or go to a local hospital for treatment while immediately notifying the project leader to get further handling advice. If treated at a local hospital, contact should be established with the attending doctor to understand the specifics and provide treatment recommendations. If necessary, log in to the IWRS system to initiate the unblinding process.

8.7.2 Reporting of SAEs

Any SAEs occurring during the trial, whether related to the investigational drug or not, must be promptly treated by the researcher and immediately reported in writing to the sponsor, followed by a detailed written follow-up report. SAE reports and follow-up reports should use the subject's identification code from the clinical trial, not the subject's real name, national ID number, or address.

Upon receiving any safety-related information from any source, the sponsor must immediately analyze and assess it, including the severity, relationship to the investigational drug, and whether it was an expected event. The sponsor should promptly report any suspected and unexpected serious adverse reactions to all investigators and clinical trial sites involved, as well as to ethics committees; the sponsor should also report these reactions to the drug regulatory authority and health authorities.

Researchers, upon receiving relevant safety information from the sponsor about the clinical trial, must promptly acknowledge and review it, consider adjusting treatment for the subject, if necessary, communicate with the subject at the earliest opportunity if needed, and report any suspected and unexpected serious adverse reactions provided by the sponsor to the ethics committee.

For reports involving death events, researchers should provide other required information to the sponsor and ethics committee, such as autopsy reports and final medical reports.

8.7.3 Pregnancy

If a subject becomes pregnant during the clinical trial, they must immediately discontinue the medication. The investigator must report the pregnancy to the sponsor within 24 hours of becoming aware and promptly inform the ethics committee. The investigator is responsible for following up on the pregnancy outcome until the termination of the pregnancy or one month after delivery, and must report the results to the sponsor.

8.7.4 Hospitalization

AEs that lead to hospitalization or extend the duration of hospitalization during a clinical trial should be considered SAEs. Any initial admission to a medical facility, even if it is shorter than 24 hours, meets this criterion. Exclusions from hospitalization include:

- Rehabilitation facilities
- Sanatoriums
- Routine emergency room admissions
- Day surgeries (such as outpatient/ day/ non-inpatient surgeries)

Hospital treatments or extended hospital stays that are unrelated to the worsening of an AE are not considered SAE. Examples include:

- Admission due to a pre-existing condition without any new AEs or worsening of the condition (e.g., for ongoing lab abnormalities that have been present since before the trial).
- Hospital stays for administrative reasons (such as annual routine check-ups).
- Hospitalizations specified by the clinical trial protocol (e.g., procedures required by the trial protocol).
- Elective hospitalizations unrelated to the worsening of an AE (such as elective cosmetic surgery).
- Scheduled treatments or surgical procedures that should be documented in the entire trial protocol and/ or the subject's baseline information.
- Hospital admissions solely for the use of blood products.

**9 Data Management**

This study utilizes an eCRF for the collection and management of research data, maintaining a complete audit trail to ensure the traceability of clinical trial data. The data management process must comply with Good Clinical Practice (GCP) standards to ensure the authenticity, integrity, and accuracy of clinical trial data.

**9.1 Developing a Data Management Plan**

Data managers, adhering to GCP guidelines and relevant clinical trial particulars, develop a data management plan. This plan records, describes, and defines the various tasks in data management, guiding the entire process. It should include aspects such as database creation, data entry, data verification, query management, medical coding, and database lock, and it should specify key timelines and clearly define the responsibilities of related personnel.

**9.2 Database Creation**

Database designers are responsible for creating the project database and conducting tests to ensure its accuracy and correctness.

**9.3 Data Entry**

Researchers at the study center or Clinical Research Coordinators (CRCs), following the "eCRF Filling Guide," perform data entry. Before data entry begins, the sponsor must train the relevant personnel at the study center to understand the content of the eCRF, familiarize themselves with the database structure and functionality, and recognize issues to avoid during entry. During the data entry process, researchers or CRCs can contact the data department at any time to resolve any questions encountered.

**9.4 Data Verification**

Data managers, following the Data Verification Plan, audit trial data. Main verification areas include missing data, logical issues, time windows, inclusion/ exclusion criteria, medication adherence, and consistency checks. Methods of data verification include eCRF logical checks, programmatic checks, manual reviews, and medical reviews. If data entering into the eCRF system does not meet logical requirements, the eCRF system or relevant personnel can issue queries that the study center must respond to. If the response is satisfactory, the query is closed. If the query remains unresolved, or if new queries arise from the updates made to the database following previous queries, the study center must provide new responses. This process repeats until all data in the database are confirmed as correct.

**9.5 Database Lock**

After drafting a data audit report based on the trial protocol and data review standards, the project manager organizes a data review meeting. The sponsor, principal investigator, statisticians, and data managers attend this meeting to review the data and partition the analysis datasets. Once the data manager completes the data lock inventory and following the database locking procedure, the statistician, sponsor, and principal investigator sign the database lock approval document, and the data manager then executes the database lock. If there is concrete evidence that unlocking the database is necessary after it has been locked, the researcher and relevant personnel must sign a database unlock document.

**9.6 Data document preservation and data transfer**

Data administrators should save data management-related documents as required, and hand over the locked database and related information to statistical analysts for statistical analysis.

**10 Sample size calculation**

Before conducting this study, we conducted a pilot trial: The change values of the total scores on the LOTCA scale at 90 days post-drug cessation compared to baseline for L-oxiracetam and oxiracetam were 26.0 ± 11.8 and 22.8 ± 10.0, respectively. The combined standard deviation (SD) for both groups was 11.0, with α set at 0.05 and 1-β at 0.8. Employing an optimal design with a sample ratio of 1:1, PASS software calculated a sample size of 190 cases for each group.

The change values of the total scores on the LOTCA scale at 90 days post-drug cessation compared to baseline for the L-oxiracetam group and the placebo group were 26.0 ± 11.8 and 20.0 ± 11.4, respectively. The combined SD for both groups was 11.6, with α set at 0.05 and 1-β at 0.8. Employing an optimal design with a sample ratio of 2:1, PASS software calculated a sample size of 204 cases for the L-oxiracetam group and 102 cases for the placebo group.

Considering potential dropouts, we increased the sample size by 15%. Therefore, the sample sizes for the L-oxiracetam group, oxiracetam group, and placebo group were set at 236, 236, and 118, respectively.

**11 Blinding**

This trial employs oxiracetam and a placebo as controls and adopts a double-blind design. Placebo medications for L-oxiracetam and oxiracetam were prepared by the sponsor to achieve blinding. Simulated agents are uniformly packaged, ensuring that drug categories cannot be distinguished by appearance and meeting preparation requirements.

Additionally, each clinical trial unit designated specific research nurses for drug dispensing and administration, ensuring they did not disclose drug allocation to participants, were not involved in trial assessments, and were solely responsible for drug preparation and administration. Unblinding procedures could be initiated through the IWRS in case of SAEs, deterioration, or disease progression in participants.

The double-blind design involves a first-level blinding, where the blind codes correspond to the treatment groups (L-oxiracetam, oxiracetam, or placebo) assigned to each case number. The randomization code list was created by the statistical unit, and the blind codes were individually sealed in duplicate, stored separately at the leading unit and the sponsor's site.

**12 Randomization**

In this trial, a block randomization method will be used with a ratio of 2:2:1, where successfully screened subjects will be randomly assigned to three groups. The study employs a centralized randomization procedure, with each center competing for participant enrollment. The Department of Biostatistics at the School of Public Health, Nanjing Medical University, provide the centralized randomization system. Following the confirmation of each eligible subject by the center's investigator, researchers at each center will log into the system, input screening data, obtain a random number and drug code, and dispense the investigational drug accordingly.

**13 Statistical Analysis of Datasets: Definition and Selection**

Full Analysis Set (FAS): The FAS operates under the Intention-To-Treat (ITT) principle, analyzing all cases that have been randomized into groups and have used the medication at least once. For cases where the entire course of treatment is not observable, the last observed data are carried forward to the final outcomes of the trial. According to the ITT principle, all patients are analyzed according to the groups into which they were randomized. This approach aims to maintain the benefits of randomization and to avoid biases that might arise from excluding patients who did not complete the treatment as originally planned.

PPS: The PPS consists of a subset of the FAS, generated from cases that have adhered closely to the trial protocol. This set includes all participants who completed the prescribed treatment and follow-up without any serious violations of the trial protocol. The precise definition of what constitutes a serious violation will be finalized during the data review process.

Safety Analysis Set (SS): The SS is used for the evaluation of safety and tolerability. It includes all participants who, after randomization, received at least one treatment and underwent at least one safety evaluation. In safety analyses, patients are analyzed based on the actual medication group they received, which helps in assessing the safety profile of the drug across different patient groups and treatment conditions. This dataset is crucial for identifying any potential adverse effects associated with the treatment.

**14 Statistical analysis plan**

SAS 9.4 was used for data analysis independently conducted by a biostatistician unaware of the allocation. Continuous variables will be presented as mean ± SD with a 95% CI for normal distribution and as median (range) for abnormal distribution. Categorical variables will be expressed by numbers and percentages. All hypothesis tests were two-sided with α = 0.05. A P-value ≤ 0.05 indicated statistical significance.

The analysis for the primary outcome was conducted using analysis of covariance (ANCOVA). The least squares means of change from baseline at 3 months in LOTCA scores, as well as the mean differences between groups (L-oxiracetam vs. oxiracetam/ Placebo) and 95% CIs were estimated. Cohen’s d and 95% CI was used to calculate the effect size. For the secondary continuous efficacy outcomes, mean differences with corresponding 95% CIs were also analyzed using ANCOVA. For categorical outcomes of GOS-E, the rate differences and 95% CIs between groups were estimated using the approximate normality method and statistically tested with Fisher's exact probability test. All models were adjusted for age, sex, education, and TBI severity at baseline. Stratified analyses were performed to explore the efficacy of the L-oxiracetam intervention on cognitive function in patients with different age (< 60 vs. 60 + years), sex (male vs. female), education (none vs. Primary vs. Secondary school or Higher), and TBI severity (mild vs. moderate). Moreover, multiplicative interactions were tested by including a cross-product item in the model for the intervention group with age, sex, education, or TBI severity.

**15 Quality Control and Assurance**

**15.1 Quality Control Measures**

Sponsors and researchers should employ standard operating procedures to ensure the implementation of quality control and assurance systems in clinical trials. All observations and findings in clinical trials should be verified to ensure the reliability of the data and that the conclusions of the clinical trials are based on original data. Quality control must be applied at every stage of data handling to ensure all data are reliable and processed correctly.

**15.2 Training of Researchers**

Before the start of the clinical trial, evaluators at each research center should undergo scale training, with training records preserved.

Prior to the start of the clinical trial, monitors in collaboration with the heads of each trial center should train researchers on the trial protocol. This training helps researchers understand and become familiar with the nature, action, efficacy, and safety of the trial drug, including information from pre-clinical studies related to the drug. They should also be kept informed of any new information related to the drug that emerges during the clinical trial.

**15.3 Enhancing Participant Compliance**

Researchers should rigorously implement informed consent, ensuring that participants fully understand the trial requirements and cooperate with the trial. Sponsors provide trial medication and cover laboratory testing costs.

Researchers should also require patients to bring all medications they are currently using during follow-ups to check for concomitant medication use and record it in the eCRF. For patients with poor therapeutic outcomes or those who do not adhere to the medication schedule, follow-up should be intensified.

**15.4 Clinical Trial Monitoring**

Monitors appointed by the sponsors should regularly visit trial hospital sites to ensure strict adherence to all aspects of the study protocol and to inspect original data to confirm consistency with the data entered in the eCRF.

**15.5 Clinical Trial Auditing**

Regulatory authorities and sponsors may delegate auditors to conduct systematic inspections of clinical trials to determine whether the execution of the trial conforms to the protocol and whether the data reported by the participating clinical units are consistent with medical records or other original records. Audits should be performed by personnel not directly involved in the clinical trial.

**16 Ethical Requirements**

Clinical trials must adhere to the Declaration of Helsinki and relevant national guidelines and regulations governing clinical trials. Before the initiation of the trial, the study protocol must be approved by the ethics committee of the responsible clinical research entity.

Before enrolling each patient in the study, the research physician is responsible for fully and comprehensively informing them or their designated guardian in writing about the purpose, procedures, and potential risks of the study. Patients must be informed that they have the right to withdraw from the study at any time. Prior to enrollment, each patient must be provided with a written informed consent form. It is the responsibility of the research physician to obtain informed consent from each patient before they participate in the study, and to retain the consent form as part of the clinical trial documentation for reference.

**17 Clinical Trial Records**

The eCRF system should only display the subject's randomization number and initials. The eCRF is used to record clinical research data of the subjects and is an integral part of the related research reports. The eCRF must be filled out by the researcher or an authorized person (as noted in the research authorization form). Before the eCRF database is locked, it must be electronically signed by the researcher or authorized person, affirming that all information in the eCRF system is accurate.

In clinical research, an eCRF form must be filled out for each examination to record the physical condition of the subjects. Medical records and other records of the subjects are kept by the researcher. These records should include original data, copies of laboratory data, and other medical test results (e.g., ECG).

In the study, subject information is not directly recorded in the eCRF system but is documented in each subject's original medical records as original data, which are then transcribed into the eCRF system.

Data in the eCRF originate from original medical records and are entered by the researcher or designated personnel, who must ensure the completeness and accuracy of the information. Any corrections or modifications to data in the eCRF system are automatically logged with the name of the person making the change and the date of the change.

After filling out the eCRF, it should be promptly submitted online. Once the data in the eCRF system has been verified through source data verification, data management review, and any queries resolved, the researcher must electronically sign off before the data is locked.

**18 Protocol Revision**

After approval by the ethics committee, any modifications to the protocol must be documented in a "Protocol Amendment Description," signed by the principal investigator, and approved by the ethics committee before implementation.

Once the protocol is amended, it must also be acknowledged and signed by the sponsor.

No one participating in the trial may deviate from the protocol.

**Appendix 1. Participating hospital**

| **No.** | **Institution Name** | **Province - City** |
| --- | --- | --- |
| 1 | Tianjin Medical University General Hospital | Tianjin |
| 2 | Beijing Chao-Yang Hospital, Capital Medical University | Beijing |
| 3 | Inner Mongolia People's Hospital | Inner Mongolia - Hohhot |
| 4 | Air Force Hospital of Northern Theater of PLA | Liaoning - Shenyang |
| 5 | The First People's Hospital of Shenyang | Liaoning - Shenyang |
| 6 | Tonghua Central Hospital | Jilin - Tonghua |
| 7 | Affiliated Hospital of Hebei University | Hebei - Baoding |
| 8 | Shanxi Provincial People's Hospital | Shanxi - Taiyuan |
| 9 | The First Affiliated Hospital of Henan University of Science and Technology | Henan - Luoyang |
| 10 | The First Hospital of Ningbo | Zhejiang - Ningbo |
| 11 | The Affiliated Hospital of Xuzhou Medical University | Jiangsu - Xuzhou |
| 12 | The First People's Hospital of Lianyungang | Jiangsu - Lianyungang |
| 13 | Suzhou Kowloon Hospital | Jiangsu - Suzhou |
| 14 | The Affiliated Huaian No.1 People’s Hospital of Nanjing Medical University | Jiangsu - Huai'an |
| 15 | Taizhou People's Hospital | Jiangsu - Taizhou |
| 16 | Northern Jiangsu People's Hospital | Jiangsu - Yangzhou |
| 17 | Affiliated Hospital of Nantong University | Jiangsu - Nantong |
| 18 | Affiliated Hospital of Jiangsu University | Jiangsu - Zhenjiang |
| 19 | The First People's Hospital of Changzhou | Jiangsu - Changzhou |
| 20 | Xiangya Hospital Central South University | Hunan - Changsha |
| 21 | Zhangzhou Municipal Hospital of Fujian Province | Fujian - Zhangzhou |
| 22 | The Second Affiliated Hospital of Guangzhou Medical University | Guangdong -Guangzhou |
| 23 | Guangzhou First People's Hospital | Guangdong -Guangzhou |
| 24 | Huizhou Central People's Hospital | Guangdong - Huizhou |
| 25 | Yichun People's Hospital | Jiangxi - Yichun |
| 26 | The Second Affiliated Hospital of Nanchang University | Jiangxi - Nanchang |
| 27 | The First Affiliated Hospital of Gannan Medical University | Jiangxi - Ganzhou |
| 28 | Haikou People's Hospital | Hainan - Haikou |
| 29 | The First People's Hospital of Nanning | Guangxi - Nanning |
| 30 | The Second Nanning People's Hospital | Guangxi - Nanning |
| 31 | Liuzhou Workers' Hospital | Guangxi - Liuzhou |
| 32 | Liuzhou People's Hospital | Guangxi - Liuzhou |
| 33 | Yan'an Hospital of Kunming City | Yunnan - Kunming |
| 34 | The People's Hospital of Yuechi County | Sichuan - Guang'an |
| 35 | The Second Affiliated Hospital of Shantou University Medical College | Guangdong - Shantou |
| 36 | Jieyang People's Hospital | Guangdong - Jieyang |
| 37 | Xianyang Hospital of Yan'an University | Shaanxi - Xianyang |
| 38 | Pu'er People's Hospital | Yunnan - Puer |
| 39 | The First Affiliated Hospital of Nanchang University | Jiangxi - Nanchang |
| 40 | Yangquan Coal Industry (Group) General Hospital | Shanxi - Yangquan |
| 41 | Zhejiang Provincial People's Hospital | Zhejiang - Hangzhou |
| 42 | Taizhou First People's Hospital | Zhejiang - Taizhou |
| 43 | Chengdu Xinhua Hospital | Sichuan - Chengdu |
| 44 | The People's Hospital of Dazhu County | Sichuan - Dazhou |
| 45 | The Affiliated Hospital of Southwest Medical University | Sichuan - Luzhou |
| 46 | The Fifth Affiliated Hospital of Guangzhou Medical University | Guangdong -Guangzhou |
| 47 | The Third Affiliated Hospital of Guangzhou Medical University | Guangdong -Guangzhou |
| 48 | Affiliated Hospital of Zunyi Medical University | Guizhou - Zunyi |
| 49 | Zhumadian Central Hospital | Henan - Zhumadian |
| 50 | Shiyan Renmin Hospital | Hubei - Shiyan |
| 51 | The Second Hospital of Tianjin Medical University | Tianjin |

**Appendix 2** **Informed Consent Form · Notification Page**

| Protocol Number: NJYK-L-ORCT-III |  |
| --- | --- |
| Research Center Name: |  |
| Research Center Address: |  |
| Patient Name: | Patient Screening Number: |

We are about to conduct a randomized, double-blind, parallel control, multicenter, Phase III clinical study on the use of L-oxiracetam vs oxiracetam to improve memory and cognitive impairment in patients with traumatic brain injury (TBI). Your condition may meet the inclusion criteria for this study, therefore, we would like to invite you to participate in this study. This informed consent form will introduce you to the purpose, steps, benefits, risks, inconvenience or discomfort of the study, etc. Please read carefully and make a careful decision on whether to participate in the study. When the researcher explains and discusses the informed consent form with you, you can ask questions at any time and let him/ her explain to you what you do not understand. You can make a decision after discussing with your family, friends, and your doctor.

If you are currently participating in other clinical studies, please inform your research doctor or research staff.

The lead unit of this study is Tianjin Medical University General Hospital, and the project is led by Director Rongcai Jiang. The sponsor of this study is Nanjing Youke Pharmaceutical Co., Ltd.

**Why is this study being conducted?**

(I) Relevant Background

With the development of modern industry, power machinery, and high-speed transportation tools, the incidence of TBI is increasing, and cognitive impairment is one of the most common and persistent sequelae of TBI. Due to the complex mechanism of acute brain injury and the difficulty of treatment, the world is exploring its treatment to promote effective recovery from brain injury. Countries in Europe, mainly Italy, have carried out research and application of nootropic drugs. This is a new type of central nervous system drug that can promote learning and memory ability. The most notable is the pyrrolidone class, its representative drug oxiracetam, was first synthesized by the Italian company in 1974, first listed in Italy in 1987, and then listed in Portugal in 1991. In 2003, the China Food and Drug Administration approved oxiracetam capsules for domestic listing, and in 2005 and 2010, oxiracetam injection and oxiracetam for injection were approved for domestic listing, respectively.

However, to date, countries around the world have different attitudes towards the use of brain injury recovery drugs. The United States and Canada do not advocate the use of any drugs to promote brain injury repair; some patients with brain trauma in Europe receive nootropic drug treatment. In our country, oxiracetam has become a commonly used neuroprotective drug for patients with nerve injury, and some patients with brain trauma are also using it. It can be confirmed that the use of oxiracetam rarely causes toxic side effects, and it can effectively promote brain injury recovery in animal experiments, but there is no standardized clinical research to confirm its obvious effectiveness. The drug in our study - oxiracetam for injection, is a single levorotatory enantiomer of oxiracetam. Preclinical studies have confirmed that the application of L-oxiracetam may reduce the clinical use dose of oxiracetam, thereby further reducing the potential toxic side effects of oxiracetam.

In view of the different uses of nootropic drugs in acute brain injury in Europe and the United States, and there is no actual evidence to prove that patients with brain trauma must use nootropic drugs, this study plans to use a placebo as a control for whether oxiracetam and L-oxiracetam are effective and safe. And it has obtained the approval of the National Drug Administration and the Ethics Committee.

(II) Purpose of the Study

The purpose of this study is to evaluate the effectiveness and safety of L-oxiracetam in improving memory and cognitive impairment in patients with acute TBI.

**Who can be invited to participate in this study?**

If you meet the following criteria: ① Age is 18-75 years old (including boundary values); ② TBI meets the following conditions: This diagnosis has a clear head injury, closed TBI, or TBI accompanied by cerebrospinal fluid otorrhea and/ or rhinorrhea and/ or intracranial pneumocephalus; magnetic resonance imaging (MRI) or computed tomography (CT) confirmed that there is intracranial hemorrhage above the cerebellar tentorium (including cerebral contusion, subarachnoid hemorrhage, epidural hematoma, subdural hemorrhage, intracerebral hematoma, etc.), with or without transient coma; The injury of TBI is light and medium (Glasgow Coma Scale (GCS) score 9-15 points); The condition is stable within 72 hours after TBI, only conservative treatment is needed, and craniotomy is not required (there can be non-general anesthesia or non-basic anesthesia intracranial pressure monitoring of brain parenchyma); ③ Simple mental state examination, that is, MMSE score is lower than normal; then you will be invited to participate in this study.

If you meet any of the following conditions, you will not be able to participate in this study. ① Known or suspected allergy to the test drug and its components. ② After the injury, drugs that improve cognitive function listed in the plan have been used. ③ There is a history of severe TBI, cerebrovascular accident, or structural cranial brain lesion. ④ There are diseases such as speech/ hearing impairment that cannot cooperate to complete cognitive function assessment. ⑤ Secondary brain injury occurred after this TBI. ⑥ Need to undergo craniotomy or ventricular drainage. ⑦ Combined with other serious organ injuries or serious complications that may affect the life of the subject. ⑧ Patients with active epilepsy within 1 year. ⑨ Combined with severe liver and kidney disease. ⑩ Combined with severe heart disease, lung disease, blood and hematopoietic system disease, gastrointestinal disease or other serious or progressive diseases. ⑪ Past or current malignant tumor (except for cured IB stage or lower cervical cancer, non-invasive basal cell or squamous cell skin cancer; breast cancer with complete remission (CR) >10 years, malignant melanoma with CR >10 years, other malignant tumors with CR >5 years).

⑫ Combined with neurological, mental diseases and unable to cooperate or unwilling to cooperate. ⑬ Pregnant, lactating women or those who have plans for childbirth in the near future. ⑭ The researcher believes that it is not suitable to participate in this clinical trial. ⑮ Participated in other clinical trials and used experimental drugs 3 months before the trial.

**How many people will participate in this study?**

This study will be conducted concurrently at multiple clinical research centers led by Tianjin Medical University General Hospital, and it is expected that 590 patients will voluntarily participate.

**How is this study conducted?**

This study will last for 14 days of medication, and you will need to follow up for 3 months after the end of the medication. If you voluntarily participate in this study, we hope you will cooperate with the following matters:

Your supervising doctor will inform you about the relevant situation of the study and answer all related questions. After signing the informed consent form, you will undergo a checkup including vital signs, and the doctor will also ask about your medical history and conduct a cognitive function assessment. You will also undergo routine blood tests (white blood cell (WBC), red blood cell (RBC), hemoglobin (HGB), platelet (PLT)), routine urine tests (protein (PRO), glucose (GLU), urinary leukocyte (LEU), urine erythrocyte (ERY)), liver function tests (aspartate aminotransferase (AST), alanine aminotransferase (ALT), total bilirubin (TBIL), gamma-glutamyl transferase (γ-GT), alkaline phosphatase (ALP)), kidney function tests (serum creatinine (Scr), glomerular filtration rate (GFR)), coagulation function tests (prothrombin time (PT), activated partial thromboplastin time ( APTT), thrombin time (TT), fibrinogen (FIB)), creatine kinase (CK), triglyceride (TG) and electrocardiogram (ECG) tests, and women of childbearing age need to undergo a blood pregnancy test. All of these tests are free of charge. After screening, if you meet the inclusion criteria and do not meet the exclusion criteria, you will be randomly assigned (like flipping a coin) to the L-oxiracetam injection group, Oxiracetam injection group, or placebo group. You have a 1/ 5 chance of entering the placebo group. The specific medication method is shown in the table below.

In the routine treatment of TBI to control intracranial hypertension and prevent complications:

| Experimental Group | L-oxiracetam for injection, 4 vials each time, once a day, intravenous drip.  Oxiracetam simulator for injection, 6 vials each time, once a day, intravenous drip. |
| --- | --- |
| Positive Control Group | L-oxiracetam simulator for injection, 4 vials each time, once a day, intravenous drip.  Oxiracetam for injection, 6 vials each time, once a day, intravenous drip. |
| Placebo Group | L-oxiracetam simulator for injection, 4 vials each time, once a day, intravenous drip.  Oxiracetam simulator for injection, 6 vials each time, once a day, intravenous drip. |

- During the medication process, if you have any discomfort or other problems, please inform your supervising doctor in time.
- After the end of the medication, you still need to cooperate with the doctor to complete routine blood tests (WBC, RBC, HGB, PLT), routine urine tests (PRO, GLU, LEU, ERY), liver function tests (AST, ALT, TBIL, γ-GT, ALP), kidney function tests (Scr, GFR), coagulation function tests (PT, APTT, TT, FIB), CK, TG and ECG tests, and conduct cognitive function and living ability assessments to understand the efficacy and safety of the drug. All these tests are free of charge.
- One month and two months after the end of the medication, the doctor will follow up with you (or your legal guardian) by phone to assess your daily living ability.
- Three months after the end of the medication, you need to come to the hospital for follow-up, accept necessary examinations including CT or MRI, vital signs, and cooperate with the doctor to complete routine blood tests (WBC, RBC, HGB, PLT), routine urine tests (PRO, GLU, LEU, ERY), liver function tests (AST, ALT, TBIL, γ-GT, ALP), kidney function tests (Scr, GFR), coagulation function tests (PT, APTT, TT, FIB), CK, TG and ECG tests, women of childbearing age need to undergo a blood pregnancy test. The doctor will also assess your cognitive function and living ability.

**What is the impact of participating in this study on the daily life of the subjects?**

When deciding whether to participate in this study, please carefully consider the possible impact of the above-listed examinations and follow-ups on your daily work, family life, etc. Consider the time and transportation issues for each return visit. If you have any questions about the examinations and procedures involved in the trial, you can consult us.

After the injury, cognitive rehabilitation treatment is not allowed. During the medication and follow-up period, the use of the following drugs that may affect the evaluation of efficacy is prohibited:

Cholinesterase inhibitors, such as Donepezil, Rivastigmine, Galantamine, Neostigmine, huperzine A, etc.;

γ-lactam brain function improvers, such as Aniracetam, Piracetam and other market-available Oxiracetam, etc.;

Other drugs: Nicergoline, Nimodipine, Ginkgo biloba preparations, cerebrolysin, Almitrine-raubasine, Ganglioside, Citicoline, Idebenone, Tyrosine, Safflower Extract and Aceglutamide Injection, Dihydroergosine mesylate, Brain protein hydrolysate, Mouse nerve growth factor, Pyrithioxin hydrochloride, Acetylglutamine, Acetamide pyrrolidone, 2-(2-Aminoethyl)isothiourea dihydrobromide, Hormone replacement therapy (growth hormone, estrogen, thyroid hormone), etc.

Before undergoing any treatment or taking any new medication, please consult your research doctor.

Considering your safety and to ensure the validity of the research results, you cannot participate in any other clinical research related to drugs and medical devices during the study period.

**What are the risks and adverse reactions of participating in this study?**

The clinical adverse reactions of oxiracetam for injection include anxiety, skin itching, rash, nausea, stomach pain, etc., which can subside on their own after stopping the medication. A few patients have experienced mental excitement and sleep abnormalities. The side effects of L-oxiracetam are similar, and due to the lower dose, there may be fewer side effects. The incidence of the above side effects is very low, and they can recover on their own after stopping the medication.

The doctor will monitor drug side effects. During the study, if you experience any side effects or discomfort, you should immediately report to the research doctor.

The doctor may give you other drugs to control side effects. If you or your research doctor believe that you cannot tolerate these side effects, the research drug may be completely discontinued, and you may withdraw from this study.

Other risks associated with research such as:

**Reproductive risks**

For female subjects: This study does not recruit pregnant or lactating women. Since Oxiracetam and L-oxiracetam may affect reproduction, participants in this study who are sexually active must use contraception and should continue to use contraception until 3 months after the last administration of the research drug.

During the study, if you or your female partner become pregnant or think you may be pregnant, you should immediately tell the research doctor so that the study will not have reproductive accidents.

**Other risks**

There may also be some currently unpredictable risks, discomfort, drug interactions, or adverse reactions.

If personal private information is inadvertently leaked, it may have a negative impact on your work, study, and life.

**What are the possible benefits for the subjects participating in this study?**

By participating in this study, you will, to a certain extent, receive more attention from senior doctors, which is beneficial for the improvement of the condition; the results of laboratory tests conducted in the study will also help you and the doctor make judgments about your own health status. At the same time, we hope that the information obtained from your participation in this study can benefit patients with the same condition as you in the future.

**Are there other alternative treatment options if not participate in this study?**

You can choose not to participate in this study, which will not have any adverse effects on your routine treatment. Currently, for your health condition, similar nootropic drugs have been used in clinical treatment, such as Piracetam, Aniracetam, etc. The treatment plan of this study is not the only treatment option for your current disease, you can discuss with your doctor and then decide whether to participate in this study.

**Is it necessary to participate and complete this study?**

Your participation in this study is entirely voluntary. If you do not wish to, you can refuse to participate, which will not have any negative impact on your current or future health care. Even if you agree to participate, you can change your mind at any time, tell the researcher to withdraw from the study, you will not be discriminated against or retaliated against for withdrawing from the trial, and it will not affect your access to normal medical services. When you decide not to participate in this study anymore, we hope you will inform your research doctor in time, and the research doctor can provide advice and guidance on your health condition.

The sponsor or regulatory agency may also terminate this study during the study period. If this study is terminated prematurely, we will notify you in time, and your research doctor will provide advice for your next treatment plan based on your health condition.

For patients who drop out halfway, for safety reasons, we have a last follow-up plan, and you have the right to refuse. If after you withdraw, new information related to your health and rights is found, we may contact you again.

In principle, after you withdraw, the researcher will strictly keep your relevant information until it is finally destroyed, and will not continue to use or disclose this information during this period. But in the following rare cases, the researcher will continue to use or disclose your relevant information, even if you have withdrawn from the study or the study has ended. These situations include: removing your information will affect the scientific nature of the research results or the evaluation of data safety; providing some limited information for research, teaching or other activities (this information will not include your name, ID number, or other personal information that can identify you); when government regulatory agencies need to supervise the study, they will request to view all research information, which will also include your relevant information when you participated in the study.

**The costs of participating in this study**

The sponsor, Nanjing Youke Pharmaceutical Co., Ltd., will pay for the examination fees related to the study during your participation in this study, the registration fee during follow-up, provide research drugs for free, and provide a follow-up subsidy of a total of 600 yuan (including 100 yuan for each telephone follow-up after discharge, a total of two times; 400 yuan for the final visit to the hospital), which will be issued after the group is disbanded.

If the patient suffers damage such as adverse reactions or serious adverse reactions related to the study, the sponsor will bear the corresponding treatment costs and economic compensation.

If you need treatment and examination for other diseases at the same time, it will not be free.

**How is research-related injury handled?**

If your health condition is harmed during your participation in this study, please inform the researcher, and we will take necessary medical measures. According to the relevant laws and regulations in our country, when a research-related injury occurs, the sponsor of this study will bear the corresponding medical expenses and provide corresponding economic compensation.

**What do I need to do if I participate in the study?**

- Provide accurate past medical history and current health information.
- Inform the doctor of any health problems that occur during the study.
- Tell the doctor about any treatment you have undergone during the study and any new drugs, medications, vitamins, or herbs you have taken.
- Unless permitted by a doctor, you should not take any medication or treatment, including prescription drugs and drugs purchased over the counter at pharmacies (including vitamins and herbs).
- Take the research medication as directed by the doctor and visit as required.
- Do not participate in other medical research.
- Take contraceptive measures acceptable to the doctor.
- Follow the guidance of researchers and research doctors.
- You can ask at any time if there is anything unclear.

**Will the personal information of the subjects be kept confidential?**

If you decide to participate in this study, your participation in the study and your personal information in the study will be kept confidential. Your blood/ urine samples will be identified by the research number rather than your name. Information that can identify you will not be disclosed to members outside the research team unless your permission is obtained. All research members and research sponsors are required to keep your identity confidential. Your files will be kept in a locked file cabinet for researchers to view. To ensure that the research is conducted in accordance with regulations, when necessary, members of government management departments or ethics committees can view your personal information in the research unit as stipulated. When the results of this study are published, your personal information will not be disclosed.

**What about new information related to the study?**

During the trial, we may learn new information about the treatment, and we will notify you in time to let you decide whether to continue to participate in the study or withdraw.

**Will the research drug treatment continue after the study ends?**

After the end of the study, Nanjing Youke Pharmaceutical Co., Ltd. (the sponsor) will no longer continue to provide you with research drugs. Your doctor will discuss your future treatment plan with you.

**Who should I contact if I have questions or difficulties?**

If you have any questions related to this study, please contact the doctor _______, contact number: _______ .

If you have questions related to the rights of the subjects themselves, you can contact _______ Medical Ethics Committee of the hospital, contact number: _______.

**Informed Consent Form · Patient Signature Page**

Subject's Informed Consent Statement

I have been informed about the research background, purpose, steps, risks, and benefits of the L-oxiracetam injection project. I have had enough time and opportunity to ask questions, and I am satisfied with the answers to the questions. I have also been informed that when I have questions or want to get more information, I should contact whom. I have read this informed consent form and agree to participate in this study. I know that I can withdraw from this study at any time during the study without any reason. I have been informed that I will receive a copy of this informed consent form, which contains my signature and the researcher's signature.

Subject's Signature: Date:

Contact Number:

Guardian's Signature: Date:

Relationship with the Subject: Contact Number:

Note: When the subject or his/ her guardian cannot read or write, at least one impartial witness must be present. The impartial witness must witness the entire process of the informed consent discussion and sign.

I confirm that the information in the informed consent form has been correctly explained and the subject and/ or the subject's legal representative understand these information. The subject voluntarily agrees to participate in this study.

Impartial Witness Signature [if applicable]

Date:

Contact Number:

Researcher's Notification Statement

I have informed the subject (and/ or his/ her guardian) about the research background, purpose, steps, risks, and benefits of the L-oxiracetam injection project, given him/ her enough time to read the informed consent form, discuss with others, and answered his/ her questions about the research; I have informed the subject of the contact information when encountering problems; I have informed the subject (and/ or his/ her guardian) that he/ she can withdraw from this study at any time during the study without any reason.

Researcher's Signature:

Date: Contact Number:

**Appendix 3. Scoring Criteria Attachments**

**Attachment 1.** **Mini-Mental State Examination (MMSE)**

| Item | | | Score | | | | | |
| --- | --- | --- | --- | --- | --- | --- | --- | --- |
| I Orientation  (10 point) | What year is it now? | |  |  |  |  | 1 | 0 |
|  | What season is it now? | |  |  |  |  | 1 | 0 |
|  | What month is it now? | |  |  |  |  | 1 | 0 |
|  | What date is it today? | |  |  |  |  | 1 | 0 |
|  | What day of the week is it today? | |  |  |  |  | 1 | 0 |
|  | Which county (district) do you live in? | |  |  |  |  | 1 | 0 |
|  | Which province do you live in? | |  |  |  |  | 1 | 0 |
|  | Which town (street) do you live in? | |  |  |  |  | 1 | 0 |
|  | Which hospital are we in now? | |  |  |  |  | 1 | 0 |
|  | What floor are we on now? | |  |  |  |  | 1 | 0 |
| II Memory  (3 point) | I will tell you three things, please repeat them after me and remember them, I will ask you again later (1 point each, total 3 points) | |  |  | 3 | 2 | 1 | 0 |
| III Attention and Calculation  (5 point) | I would like you to count backward from 100 by sevens.Subtract 5 times continuously (93, 86, 79, 72, 65. 1 point each, total 5 points. If wrong, but the next answer is correct, only count one error) | | 5 | 4 | 3 | 2 | 1 | 0 |
| IV Recall  (3 point) | Now, can you tell me the three things I just told you to remember? | |  |  | 3 | 2 | 1 | 0 |
| V Language Ability  (9 point) | Naming Ability | Show a watch, ask the patients to name it. |  |  |  |  | 1 | 0 |
|  |  | Show a pen, ask the patient to name it. |  |  |  |  | 1 | 0 |
|  | Repetition Ability | I will say a sentence now, please repeat it clearly after me (sì shí sì zhī shí shī zi) |  |  |  |  | 1 | 0 |
|  | Reading Ability | (Close your eyes) Please read this sentence and do as it says. |  |  |  |  | 1 | 0 |
|  | Three-step Command | I will give you a piece of paper, please do as I say, start now: “Take the paper in your right hand, fold it in half with both hands, and place it on your left leg.” (1 point for each action, total 3 points) |  |  | 3 | 2 | 1 | 0 |
|  | Writing Ability | Make up and write a complete sentence about anything. |  |  |  |  | 1 | 0 |
|  | Structural Ability | Please copy this picture. |  |  |  |  | 1 | 0 |
| Total Score |  | | | | | | | |

**Operating Instructions**

I. Orientation (Maximum: 10 points)

First, ask about the date, then ask other parts specifically, such as "Can you tell me what season it is now?", each correct answer gets one point. Please ask in order, "Can you tell me which province you live in?" (county, street, where, which floor), each correct answer gets one point.

II. Memory (Maximum: 3 points)

Tell the testee that you will ask a few questions to check his/ her memory, then clearly and slowly say the names of 3 unrelated things (such as: ball, national flag, tree, about 1 second for each). After saying all 3 names, ask the testee to repeat them. The testee's score depends on their first repetition of the answer. (1 point for each correct answer, up to 3 points). If they can't remember all, you can repeat, but the number of repetitions cannot exceed 5 times. If they still can't remember all 3 names after 5 times, then the check for recall ability is meaningless. (Please skip part IV "Recall Ability" check).

III. Attention and Calculation (Maximum: 5 points)

Ask the patient to start from 100 and subtract 7, then subtract 7 again, subtract 5 times in total (i.e., 93, 86, 79, 72, 65). Each correct answer gets 1 point, if the previous one was wrong, but the next answer is correct, also get 1 point.

IV. Recall Ability (Maximum: 3 points)

If the testee completely remembered the 3 names last time, now let them repeat them again. Each correct repetition gets 1 point, up to 3 points.

V. Language Ability (Maximum: 9 points)

1. Naming Ability (0-2 points): Show the watch card to the testee and ask them what this is? Then show the pen and ask them the same question.

2. Repetition Ability (0-1 point): Ask the testee to pay attention to what you say and repeat it once, note that only one repetition is allowed. This sentence is "sì shí sì zhī shí shī zi", only correct, clear pronunciation can score 1 point.

3. Three-step Command (0-3 points): Give the testee a blank piece of paper, ask them to do as you command, note not to repeat or demonstrate. Only the actions they do in the correct order are correct, each correct action scores 1 point.

4. Reading Ability (0-1 point): Show a "Close your eyes" card to the testee, ask the testee to read it and do as required. Only if they actually close their eyes can they score.

5. Writing Ability (0-1 point): Give the testee a piece of white paper, let them spontaneously write a complete sentence. The sentence must have a subject, a verb, and make sense. Note that you cannot give any hints. Grammar and punctuation errors can be ignored.

6. Structural Ability (0-1 point): On a piece of white paper, there are two intersecting pentagons, ask the testee to draw them accurately. Evaluation criteria: The pentagon needs to draw 5 clear angles and 5 sides. At the same time, the intersection of the two pentagons forms a diamond, the jitter of the lines and the rotation of the figure can be ignored.

**Attachment 2. Montreal Cognitive Assessment (MoCA）**

| Visual Space and Executive Function | | | | | | | | | | | | | | | | | | | | | | | Score |
| --- | --- | --- | --- | --- | --- | --- | --- | --- | --- | --- | --- | --- | --- | --- | --- | --- | --- | --- | --- | --- | --- | --- | --- |
|  | E A  5 End  2  B  1  Start  D 4  3  C | | | | | | Copy Cube | | | | Draw Clock(**10 past 11**  ）(3 point) | | | | | | | | | | | | / 5 |
|  |  |  |  | [ ] |  |  |  |  |  | [ ] | Outline[ ] Pointer[ ] Number[ ] | | | | | | | | | | | |  |
| Naming | | | | | | | | | | | | | | | | | | | | | | |  |
| 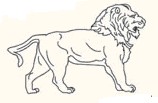 | | |  | 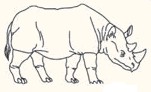 | | | | | |  |  | 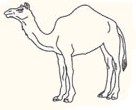 | | | | | | | | | | | / 3 |
|  |  | [ ] |  |  |  |  |  |  | [ ] | |  |  | |  | | | |  |  | | [ ] | |  |
| Memory | | Read the following words, then have the patient repeat the process 2 times, recall after 5 minutes. | |  | | Face | | | Velvet | | Church | | | | Chrysanthemum | | | | | | | Red | Not counted |
|  |  |  |  | First time | |  | | |  | |  | | | |  | | | | | | |  |  |
|  |  |  |  | Second time | |  | | |  | |  | | | |  | | | | | | |  |  |
| Attention | | Read the following numbers, please have the patient repeat (1 per second). | | | | | | | | | | | Forward[ ] | | | | | | | **21854** | | | / 2 |
|  |  |  |  |  |  |  |  |  |  |  |  |  | Backward[ ] | | | | | | | **742** | | |  |
| Read the following numbers, every time the number 1 appears, the patient knocks on the table once, if the number of errors is greater than or equal to 2, do not give. | | | | | | | | [ ]52139411806215194511141905112 | | | | | | | | | | | | | | | / 1 |
| Serail 7 subtraction starting at 100. | | | | [ ]93 | [ ]86 | | | | | [ ]79 | | | [ ]72 | | | | [ ]65 | | | | | | / 3 |
| 4~5 correct give 3, 2~3 correct give 2, 1 correct give 1, all wrong is 0. | | | | | | | | | | | | | | | | | | | | | | |  |
| Language | | Language Repeat: I only know that Zhang Liang came to help today. [ ] When the dog is in the room, the cat always hides under the sofa [ ] | | | | | | | | | | | | | | | | | | | | | / 2  / 1 |
|  |  | Fluency:Say as many animal names as possible in 1 minute. [ ]  (N≥11 Name) | | | | | | | | | | | | | | | | | | | | |  |
| Abstract | | Similarity: Like Banana—Orange=Fruit [ ]Train—Bicycle [ ]Watch—Ruler | | | | | | | | | | | | | | | | | | | | | / 2 |
| Delayed Recall | | Cannot prompt when recalling | | Face  [ ] | | Velvet  [ ] | | | Church  [ ] | | Chrysanthemum [ ] | | | | | Red  [ ] | | | | Only based on non-prompted memory | | | / 5 |
|  |  | Class prompt: | |  | |  | | |  | |  | | | | |  | | | |  |  |  |  |
|  |  | Multiple-choice prompt: | |  | |  | | |  | |  | | | | |  | | | |  |  |  |  |
| Orientation | | Date[ ] Month[ ] Year[ ] Day of the week[ ] Place[ ] City[ ] | | | | | | | | | | | | | | | | | | | | | / 6 |
| Total Score | |  | | | | | | | | | | | | | | | | | | | | | / 30 |

**MoCA Use and Evaluation Guide**

**1:Alternating Line Test**

Instructions: "Sometimes we use '123...' or English 'ABC...' to indicate order. Please draw a line in order from number to English character and gradually increasing. Start here [point to number (1)], from 1 to A, then to 2, and keep going until it ends here [point to English character (E)]."

Evaluation: When the patient completely follows the order of "1-A-2-B-3-C-4-D-5-E" for the line and there are no crossed lines, give 1. When the patient makes any mistakes and does not immediately correct them, give 0.

**2:Visual Structural Skills (Cube)**

Instructions (examiner points to the cube): "Please draw this picture again in the blank space below as accurately as possible."

Evaluation: When it fully meets the following standards, give 1: The figure is

three-dimensional

All lines are present

with no extra lines

Relative sides are basically parallel, length is basically consistent (rectangle or prism is also correct)

If any of the above standards are violated, it is 0.

**3:Visual Structural Skills (Clock)**

Instructions: "Please draw a clock here, fill in all the numbers and indicate 10 past 11." Evaluation: When it meets the following three standards, give 1:

Outline (1 point): The surface must be a circle, allowing minor defects (such as, the circle is not closed).

Numbers (1 point): All numbers must be complete and no extra numbers; the order of numbers must be correct and within their quadrant; Roman numerals can be used; numbers can be placed outside the circle.

Pointer (1 point): There must be two pointers and they point to the correct time together; the hour hand must be significantly shorter than the minute hand; the center intersection of the pointers must be inside the clock and close to the center of the clock.

If any of the above items are violated, do not give points for that item.

**4:Naming**

Instructions: From left to right, ask the patient while pointing at the picture: "Can you tell me the name of this animal?"

Evaluation: Give 1 for each correct answer. The correct answers are: (1) Lion; (2) Rhino; (3) Camel or Dromedary.

**5:Memory**

Instructions: The examiner reads 5 words at a rate of 1 word per second and tells the patient: "This is a memory test. In the following time, I will read you a few words, you need to listen carefully, you must remember. When I finish reading, tell me the words you remember. When answering, say whatever you think of, you don't have to follow the order I read." Mark the words the patient answered correctly in the first trial column. When the patient has answered all the words, or can no longer recall, read these 5 words again, and tell the patient: "I will read these words again, try to remember and tell me the words you remember, including the words you have already said in the first time." Mark the words the patient answered correctly in the second trial column.

After the second trial ends, tell the patient that he will be asked to recall these words later: "At the end of the examination, I will ask you to recall these words again."

Evaluation: These two recalls are not scored.

**6: Attention**

Number Forward Span: Instructions: "Now I'm going to say some numbers, you listen carefully, when I finish, you repeat them in the same way." Read these 5 numbers at a rate of 1 number per second.

Number Backward Span: Instructions: "Now I'm going to say some more numbers, you listen carefully, but when I finish, you must repeat them in reverse order." Read these 5 numbers at a rate of 1 number per second.

Evaluation: Accurate repetition, give 1 for each number series (note: the correct answer for backward is 2-4-7).

Alertness: Instructions: The examiner reads the number string at a rate of 1 per second and tells the patient: "Now I'm going to read a series of numbers, please listen carefully. Every time I read 1, you clap your hands. Don't clap your hands when I read other numbers."

Evaluation: If it is completely correct or only one mistake is made, give 1, otherwise do not give (error is when the patient does not clap when reading 1, or claps when reading other numbers).

Continuous Minus 7: Instructions: "Now please do a calculation problem, subtract a 7 from 100, then subtract another 7 from the result, keep subtracting until I ask you to stop." If necessary, you can explain to the patient again.

Evaluation: This item totals 3. All wrong scores 0, one correct scores 1, two to three correct scores 2, four to five correct scores 3. From 100 start to calculate the correct subtraction, each subtraction is evaluated separately, that is to say, if the patient subtracts wrong once, and all the subsequent subtractions of 7 are correct, then the subsequent correct subtractions should be given. For example, if the patient's answer is 93-85-78-71-64, 85 is wrong, but all other results are correct, so give 3.

**7: Sentence Repetition**

Instructions: "Now I'm going to say a sentence to you, after I finish, please repeat what I said as exactly as possible [pause for a while]: I only know that Zhang Liang came to help today." After the patient answers, "Now I'm going to say another sentence, after I finish, please also repeat it as exactly as possible [pause for a while]: When the dog is in the room, the cat always hides under the sofa."

Evaluation: Correct repetition, give 1 for each sentence. The repetition must be accurate. Pay attention to the omission (such as, omitting "only", "always") and replacement/ addition (such as "I only know that Zhang Liang..." said as "I only know Zhang Liang today..."; or "room" said as "house", etc.) in the repetition.

**8: Word Fluency**

Instructions: "Please say as many animal names as you know as quickly and as much as possible. The time is 1 minute, please think about it, are you ready? Start." Stop after 1 minute.

Evaluation: If the patient says ≥11 animal names within 1 minute, give 1. At the same time, record the patient's answer content on the back of the check table or on both sides. Dragon, Phoenix, Kirin and other deified animals are also correct.

**9: Abstract**

Let the patient explain in what way each pair of words are similar, or what they have in common. Start with the example words. Instructions: "Can you tell me in what way oranges and bananas are similar?" If the patient's answer is a specific feature (such as,

Both have skin, or both can be eaten, etc.), then you can only prompt once: "Please change another way, in what way are they similar?" If the patient still does not give an accurate answer (fruit), then say: "What you said is not wrong, you can also say they are both fruits." But do not give any other explanation or explanation.

After the exercise is over, say: "Can you tell me in what way trains and bicycles are similar?" After the patient answers, proceed to the next group of words: "Can you tell me in what way watches and rulers are similar?" Do not give any other explanation or inspiration.

Evaluation: Only evaluate the answers to the last two groups of words. Correct answer, give 1 for each group of words. Only the following answers are considered correct:

Train and bicycle: transportation tool; travel tool. Watch and ruler: measuring instrument; used for measurement.

The following answers cannot be given:

Train and bicycle: both have wheels. Watch and ruler: both have numbers.

**10: Delayed Recall**

Instructions: "Just now I read you a few words to remember, please try to recall again, tell me what these words are?" Mark the words that are correctly recalled without prompting in the blank column below.

Evaluation: Correctly recall the words without prompting, give 1 for each word.

Optional Items:

After the delayed free recall, for words that cannot be recalled, encourage the patient to recall as much as possible through semantic Classification cues.

For those who recall correctly through Classification prompts or multiple-choice prompts, mark a check (√) in the corresponding blank column. If they still can't recall after the Classification prompt, proceed with the multiple-choice prompt. For example: "Which of the following words is one that you remembered: nose, face, palm?"

The Classification prompts and/ or multiple-choice prompts for each word are as follows:

Scoring: Cue recall is not scored. Cue recall is only used for clinical purposes, providing further information for the examiner to analyze the type of memory impairment in the patient. For memory deficits caused by retrieval impairment, cues can improve recall performance; if it is encoding impairment, then cues do not help improve recall performance.

Red, Blue, Green

Red: A type of color

Rose, Chrysanthemum, Peony

Chrysanthemum: A type of flower

Church, School, Hospital

Church: A building

Cotton cloth, Dacron, Velvet

Velvet: A type of textile

Nose, Face, Palm

Face: Part of the body

Multiple-choice Prompt

Classification Prompt

**11:Orientation**

Instructions: "Tell me what today's date is." If the patient's answer is incomplete, you can prompt the patient: "Tell me what it is now [which year, which month, the exact date today, which day of the week]." Then ask again: "Tell me what this place is, and which city it is in?"

Evaluation: Give 1 for each correct answer. The patient must answer the exact date and location (name of the hospital, clinic, office). If the date is one day more or one day less, it is considered wrong, and no points are given.

**Attachment 3. Loewenstein Occupational Therapy Cognitive Assessment (LOTCA)**

**Loewenstein Occupational Therapy Cognitive Assessment (LOTCA)**

| Assessment Items | Method Summary |
| --- | --- |
| A.Orientation | |
| 1. Orientation to place | Ask the patient about their current location, city, home address, and where they stayed before admission. |
| 1. Orientation to time | Ask the patient about the day of the week, month, year, season, estimate the current time without looking at the clock, and how long they have been in the hospital. |
| B.Visual Perception | |
| 1. Object Identification | Let the patient identify 8 kinds of daily necessities pictures through naming, understanding, approximate pairing, and identical pairing: chair, teapot, watch, key, shoe, bicycle, scissors, glasses. |
| 1. Shape Identification | Let the patient recognize 8 different shapes of geometric figures through naming, understanding, approximate pairing, and identical pairing: square, triangle, circle, rectangle, diamond, semicircle, trapezoid, and hexagon. |
| 1. Overlapping Figures | Let the patient recognize the overlapping figure of banana, apple, pear; pliers, saw, hoe. |
| 1. Object Consistency | Let the patient identify 4 pictures of objects taken from special angles: car, hammer, telephone, and fork. Give the front windshield of the car, the back of the telephone, the side of the fork, the side of the hammer. |
| C. Spatial Perception | |
| 7. Body Orientation | Let the patient stretch out their right hand and left foot in turn; touch the opposite ear and thigh with their hand. |
| 8. Spatial Relationship with Surrounding Objects | Let the patient point out four different objects in four different directions in the room: front, back, left, and right. |
| 9. Spatial Relationship in Pictures | Show the patient a picture, then say the names of the objects in front, behind, left, and right of the character in the picture. |
| D. Praxis | |
| 10. Motor Imitation | Let the patient imitate the examiner's actions. |
| 11. Utilization of Objects | Let the patient demonstrate how to use 4 groups of objects: comb, scissors and paper, envelope and paper, pencil and eraser. |
| 12. Symbolic Actions | Let the patient demonstrate how to brush teeth, open the door with a key, cut bread with a dinner knife, make a phone call. |
| E. Visuomotor Organization | |
| 13. Copying Geometric Forms | Let the patient copy a circle, triangle, diamond, cube, and a composite figure. |
| 14. Reproducing a Two-dimensional Model | Let the patient draw geometric figures according to the given pattern, including a circle, a rectangle (square), two triangles, and some related shapes. |
| 15. Constructing a Pegboard Design | Let the patient insert pegs on the plastic pegboard to create the corresponding figure according to the given pattern. |
| 16. Constructing a Colored Block Design | Let the patient use colored blocks to piece together the corresponding three-dimensional figure according to the given pattern. |
| 17. Constructing a Plain Block Design | Ask the patient to piece together a three-dimensional figure according to the given pattern using colorless blocks, and state how many blocks are needed. |
| 18. Reproducing a Puzzle | Ask the patient to piece together a colored butterfly using 9 pieces of pattern fragments according to the given pattern. |
| 19. Drawing a Clock | Ask the patient to draw a clock on a piece of paper with a circle, mark the numbers, and indicate the position of the hour and minute hands at 10:15. |
| F. Thinking Operation | |
| 20. Item Classification | Ask the patient to categorize and name 14 items provided (sailboat, helicopter, airplane, bicycle, ship, train, car, hammer, scissors, needle, screwdriver, sewing machine, hoe, rake) according to different principles. |
| 21. Riska Object Classification (Unstructured) | Ask the patient to categorize 18 plastic pieces of three different colors (dark brown, light brown, cream) and three different shapes (arrow, ellipse, 1/ 4 sector) according to a certain intention (such as color or shape). |
| 22. Riska Object Classification (Structured) | Similar to 21, the difference is that the patient categorizes 18 plastic pieces according to the categorization method demonstrated by the examiner. |
| 23. Picture Sequence A | Give the patient 5 pictures that are out of order but related in content, ask the patient to arrange them in a logical order, and describe the story plot. |
| 24. Picture Sequence B | Give the patient another 6 pictures that are out of order but related in content, ask the patient to arrange them in a logical order, and describe the story plot. |
| 25. Geometric Sequence | Show the patient a set of geometric figures that change according to a certain rule, ask the patient to continue arranging according to the arrangement rule of the figures. |
| 26. Logical Questions | Ask the patient to look at four logical questions (one at a time), and then answer. For example: Zhang Ming was born in 1930, in which year should he be 35 years old? Xiao Li has 5 apples, Xiao Shan has 3 less than Xiao Li, how many apples do they have in total? |
| G. Attention and Concentration | |
| Evaluate the patient's attention and concentration during the entire evaluation process. | |

**LOTCA Operation and Scoring Standards**

**I. Instructions for Use**

1. Before using LOTCA, please carefully read the descriptions and correct operation methods for each test.

2. The scores for most test items range from 1 point (lowest) to 4 points (highest), except for the -F test items:

① The three item classification test items are scored from 1 point (lowest) to 5 points (highest); ② The two orientation test items are scored from 1 point (lowest) to 8 points (highest).

3. In each test, the test subject and the examiner should sit side by side, but the following test items should adopt a face-to-face seating method: ① Spatial perception items and ② Motor application items.

4. After each test item is finished, the examiner should ask the test subject: "Is this item completed?" and then score the test subject. The blank space behind each test item on the scoring form is used to fill in notes.

5. Patients with brain injuries are prone to fatigue. Some patients will express it when they are tired, but some are not aware of their own fatigue. Therefore, if the examiner finds that the test subject's actions are slowing down or restless, the test should be paused and continued after some time. At the end of the evaluation, the examiner should record the time required for the evaluation and whether the evaluation was completed in stages.

6. The examiner should objectively evaluate the level of attention and concentration of the test subject based on the observation of the test subject during the entire evaluation process.

**II. Scoring Standards**

**A. Orientation**

If the test subject's understanding ability is problematic (such as sensory aphasia), this item cannot be evaluated. If the test subject's comprehension is good but only has difficulty expressing, they can choose "yes" or "no" from the multiple choices provided by the examiner.

1. Orientation to place

Method: The examiner asks the test subject the following questions.

(1) Where are you now?

(2) What city are we in now?

(3) Where do you live? What is your exact address?

(4) Where were you before you came here?

For patients with language or memory impairments, multiple choices can be used. The examiner provides 3 options for the test subject to choose from, including one correct answer.

Scoring: The test subject gets 2 points for each correct answer; if they can only answer correctly after being given multiple choices, they get 1 point.

Minimum score: 1 point (all answers are wrong, or only one question is answered correctly after giving multiple choices):

Maximum score: 8 points (no need to give any options, all questions are answered correctly).

2. Orientation to time

Method: The examiner asks the test subject the following questions.

(1) What day of the week is it today? What month is it now? What year is it?

(2) What season is it now?

(3) What time is it now?

(4) How long have you been in the hospital? (If the test subject has not been hospitalized, the examiner can ask: "How long have you been sick?" or "How long have you been feeling unwell?")

For patients with language or memory impairments, multiple choices can be used. The examiner will provide 3 options for the test subject to choose from, including one correct answer.

Scoring: The test subject gets 2 points for each correct answer; if they can only answer correctly after being given multiple choices, they get 1 point.

Minimum score: 1 point (all answers are wrong, or only one question is answered correctly after giving multiple choices);

Maximum score: 8 points (no need to give any options, all questions are answered correctly).

**B.Visual Perception**

3. Object Identification

The object recognition cards (blue) in the test box and pages 1-4 of the test album are needed. Method:

● Naming: The examiner shows the test subject 8 cards of daily necessities: chair, teapot, watch, key, shoe, bicycle, scissors, glasses, and asks the test subject to say the name of each item. Note: Arrange the cards in the order provided above for questioning, do not use numbers on the back of the cards to arrange the order.

● Understanding: If the test subject has difficulty expressing and cannot say the name of the item, the examiner can open pages 1-2 of the test album, say the name of an item, and let the test subject point it out in the album. The examiner will ask: Which one is the chair; Which one is the watch; etc. Ask each of the 8 items one by one.

● Approximate Pairing: If the test subject has problems in understanding, the examiner takes out pictures similar to pages 1-2 of the test album, shows the test subject 8 cards one by one, and asks one by one: "Which one is this in the album?" The test subject is required to point out the item similar to a card in the album.

● Identical Pairing: If the test subject cannot distinguish similar objects, the examiner opens pages 3-4 of the album (this set of items is exactly the same as the items on the card). The examiner asks one by one: "Which one is this card in the album?" The test subject is required to pair the item on the card with the album.

Scoring:

1 point: Through the method of identical pairing, the test subject can only identify a few items (less than 4).

2 points: Through the method of identical pairing, the test subject can identify 5-8 items.

3 points: Through naming, understanding, and approximate pairing, the test subject can identify at least 4 items (4-7).

4 points: Through naming, understanding, and approximate pairing, the test subject can identify all items.

4. Shape Identification

The shape recognition cards (yellow) in the test box and pages 5-8 of the test album are needed. Method:

● Naming: The examiner shows the test subject 8 shapes on the cards one by one: square, triangle, etc. The test subject is asked to say the name of each shape.

● Understanding: If the test subject has difficulty expressing and cannot say the name of the shape, the examiner can open pages 5-6 of the test album and let the test subject point out the same shape on the album as the card. For example, "Please point out which shape is a circle", etc. The test subject is required to point out the corresponding shape.

● Approximate Pairing: If the test subject has problems in understanding and cannot recognize the shape, the examiner opens page 7 of the test album with similar shapes, shows the test subject 8 shape cards one by one, and asks the test subject to point out the shape on the album that is similar to the card.

● Identical Pairing: If the test subject cannot distinguish similar objects, then the examiner opens page 8 of the album. The shapes on page 8 are exactly the same as the shapes on the card. The examiner asks: "Which shape is this on the album?" The test subject is required to point out the matching item.

Note: The examiner shows the picture to the test subject in the exact same position as the shape on the test album. Scoring:

1 point: Through the method of identical pairing, the test subject can only identify a few items (less than 4).

2 points: Through the method of identical pairing, the test subject can identify 5-8 items.

3 points: Through naming, understanding, and approximate pairing, the test subject can identify at least 4 items (4-7).

4 points: Through naming, understanding, and approximate pairing, the test subject can identify all items.

Note: Arrange the cards and ask questions in the order provided above, do not use numbers on the back of the cards to arrange the order.

5. Overlapping Figures

The overlapping shape cards (green) in the test box and pages 9-10 of the test album are needed.

Method: The examiner shows the test subject two overlapping shape recognition cards, each recognition card has three objects overlapping: banana, pear, apple; pliers, hoe, saw.

The examiner asks the test subject: "What is drawn on the card?" If the test subject has difficulty recognizing geometric shapes, the examiner shows the test subject six individual item pictures on the test album, and then guides the test subject to answer: "Please point out in the album what you see on the card." The operation for the second card is the same.

Scoring:

1 point: Without the help of the album, the test subject cannot identify any items, or with the help of the album, the test subject can identify fewer than 3 items.

2 points: With the help of the album, the test subject can identify 3 items.

3 points: Without the help of the album, the test subject can identify at least 4 items, or with the help of the album, the test subject can identify all items.

4 points: Without the help of the album, the test subject can identify all items on the card.

6. Object Consistency

The test album's pages 11-19 are needed.

Method: The examiner shows the test subject four photos (page 11), the objects in the photos (car, hammer, telephone, and fork) are all taken from angles that are slightly different from what is normally seen, the examiner will ask the test subject for each photo: "What do you see in this photo?"

Only when the test subject has a language barrier (such as aphasia), the examiner can use multiple-choice pictures (pages 12-19). For example, let the test subject first look at the large picture on page 12, and then ask the test subject: "Please point out in these small pictures (page 13), the object you see in the large picture."

Each question only has one correct answer.

Scoring:

1 point: The test subject cannot identify any one object, or can only identify one of them.

2 points: The test subject can identify 2 objects.

3 points: The test subject can identify 3 objects.

4 points: The test subject can identify all 4 objects.

**C. Spatial Perception**

Method: The examiner and the test subject sit face to face.

7. Body Orientation

Method: The examiner can switch "left" and "right" according to the test subject's body problems. The examiner asks the test subject:

(1) Stretch out your right hand.

(2) Stretch out your left foot.

(3) Put your right hand on your left ear.

(4) Put your left hand on your right thigh.

Scoring: Each correct response gets 1 point. The minimum score is 1 point, and the maximum score is 4 points.

8. Spatial Relationship with Surrounding Objects

Method: The examiner points out four different objects in four different directions (left, right, front, back) in the room to the test subject, and then asks the test subject:

(1) On which side of you? (For example: door)

(2) On which side of you? (For example: window)

(3) On which side of you? (For example: where I sit)

(4) On which side of you? (Any obvious object in the room)

Scoring: Each correct answer gets 1 point. The minimum score is 1 point, and the maximum score is 4 points.

9. Spatial Relationship in Pictures

The photo in the test box is needed.

Method: The examiner shows the test subject a photo, in which there is a man sitting in front of a table. The examiner asks the test subject:

(1) What is in front of this person?

(2) What is on the left side of this person?

(3) On which side of this person is the computer?

(4) What is behind this person?

Scoring: Each correct answer gets 1 point. The minimum score is 1 point, and the maximum score is 4 points.

**D. Praxis**

Motor application includes three groups of content: action imitation, use of objects, and symbolic actions.

10. Motor Imitation

Method: The examiner and the test subject sit face to face. The examiner tells the test subject: “Please imitate my actions, just like looking in a mirror.” If the test subject does not understand, the examiner can further explain with actions: “If I make a movement with my left hand, please make the same movement with your right hand.”

The examiner performs the following actions:

(1) Pinch the earlobe on the same side with the thumb and index finger of one hand.

(2) Continuous action: Put the palm on the back of the neck, and then put it on the shoulder on the opposite side.

(3) Put the back of one hand on the cheek on the opposite side (fingers straight).

(4) The thumb first touches the middle finger, then touches the ring finger, and repeats the above actions 3 times.

Scoring: Each correct response gets 1 point. The minimum score is 1 point, and the maximum score is 4 points.

Note: The mirror relationship in action imitation is mainly to test the use of actions, not the recognition of the left and right sides of the body. Therefore, whether the action is mirrored or on the opposite side, as long as the action is correct, full marks can be obtained.

11. Utilization of Objects

Method: The examiner shows the test subject one group of items at a time: a comb; a pair of scissors and a piece of paper; an envelope and a piece of paper; a pencil and an eraser. The examiner says to the test subject: "Please demonstrate how to use these items." When using the pencil and eraser, the examiner says to the test subject: "Please draw a straight line on the paper, and then erase it."

Scoring: Each correct response gets 1 point. The minimum score is 1 point, and the maximum score is 4 points.

12. Symbolic Actions

Method: The examiner asks the test subject:

(1) Please demonstrate to me how you brush your teeth. (The examiner asks the test subject to demonstrate the entire action, from applying toothpaste to the toothbrush, putting the toothbrush in the mouth, to the brushing action)

(2) Please demonstrate to me how you open the door with a key.

(3) Please demonstrate to me how you cut bread with a dinner knife.

(4) Please demonstrate to me how you make a phone call. (The examiner asks the test subject to demonstrate the entire action, from picking up the handset, dialing, to putting the handset to the ear)

Scoring: Each correct demonstration gets 1 point. The minimum score is 1 point, and the maximum score is 4 points.

**E. Visuomotor Organization**

In this part of the test, the test time needs to be recorded and filled in the score sheet.

13. Copying Geometric Forms

The geometric figure cards (orange) in the test box are needed.

Method: The examiner puts a piece of paper and a pencil in front of the test subject, and says to the test subject: "I will show you 5 figures, please draw these 5 figures on the paper." The examiner arranges the figures in the following order: circle, triangle, diamond, cube, and a composite figure.

Scoring: 1 point: Cannot draw any figure, or can only draw one of them.

2 points: Can draw 2 or 3 figures.

3 points: Can draw 4 figures.

4 points: Can draw 5 figures.

Note: When drawing a cube, the test subject must accurately draw the position of each edge of the figure to score. That is to say, the test subject should show a sense of three-dimensional space of the figure.

Please arrange the cards in the order provided above for questioning, do not use numbers on the back of the cards to arrange the order.

14. Reproducing a Two-dimensional Model

The 16th page of the test album is needed.

Method: The examiner shows the test subject a geometric pattern, including a circle, a rectangle (square), two triangles, and some related shapes. The examiner asks the test subject: "Draw this pattern next to this pattern." If the test subject cannot do this, the examiner can guide the test subject: "Draw directly on this pattern."

Scoring: 1 point: Cannot draw the pattern.

2 points: Can only draw directly on the pattern.

3 points: Can draw the pattern, but after repeated attempts and errors.

4 points: Can draw the pattern.

15. Constructing a Pegboard Design

The 17th page of the test album is needed.

Method: The examiner places the following tools in front of the test subject: a socket board, some plastic pegs, and the triangular pattern design on the 17th page of the test album. The examiner asks the test subject: "Use the pegs to complete the corresponding pattern on the socket board."

Scoring: 1 point: Cannot complete.

2 points: Can only complete vertical and horizontal lines, cannot complete diagonal lines and/ or the pattern does not have corners.

3 points: Can complete the pattern, but the pattern is not correctly positioned on the socket board (see page 17).

4 points: Can correctly complete the pattern.

16. Constructing a Colored Block Design

The 18th page of the test album is needed.

Method: The examiner places the following tools in front of the test subject: 10 colored blocks and the pattern on the 18th page of the test album. The examiner asks the test subject: "Please piece together the model according to the pattern."

Scoring: 1 point: Cannot complete.

2 points: Can only establish a flat model on the table, without height or depth, or part of it is a flat model.

3 points: The established model only has height or only has depth.

4 points: Can correctly complete the puzzle.

17. Constructing a Plain Block Design

The 19th page of the test album is needed.

Method: The examiner places the following tools in front of the test subject: 10 five-color blocks and the pattern on the 19th page of the test album. The examiner asks the test subject:

1. How many blocks are needed to complete this model?
2. Please start the puzzle.

Scoring: 1 point: Cannot complete the puzzle and cannot answer the number of blocks correctly.

2 points: Only the blocks that are seen are pieced together, ignoring the blocks that are not visible behind.

3 points: The number of blocks answered is wrong, but the model can be correctly pieced together: or the number of blocks answered is correct, but the model cannot be correctly pieced together.

4 points: Can correctly complete the puzzle.

18. Reproducing a Puzzle

The 20th page of the test album is needed.

Method: The examiner shows the test subject the colored butterfly pattern on the 20th page of the test album and the corresponding 9 pieces of pattern fragments, and asks the test subject to piece together the broken pictures on the pattern.

Scoring: 1 point: Cannot complete.

2 points: Can only piece together the vertical three fragments in the middle of the pattern.

3 points: After repeated attempts, the pattern can be correctly pieced together.

4 points: Can correctly piece together the pattern without needing to attempt.

19. Drawing a Clock

The 21st page of the test album is needed.

Method: The examiner gives the test subject a pencil and a piece of paper with a circle drawn on it (like the 21st page of the test album), and then says to the test subject: "Please write out the numbers inside the clock." Then draw the correct position of the hour hand and minute hand according to 10:15.

Scoring: 1 point: Cannot complete.

2 points: The clock face drawn roughly conforms to the intention, but the scale and the time marked are deviated.

3 points: The scale of the clock face drawn is correct, but the time marked is wrong: or the time marked is correct, but the position of the clock scale is incorrect.

4 points: Can correctly complete.

**F. Cognitive Operations**

20. Item Categorization

The item classification cards (red) in the test box are needed.

Method: The examiner randomly spreads 14 cards printed with the following items on the table: sailboat, helicopter, airplane, bicycle, ship, train, car, hammer, scissors, needle, screwdriver, sewing machine, hoe, rake.

Then the examiner asks the test subject: (1) Please group the cards by the type of items.

(2) Please name each group.

After the test subject completes the first grouping operation, the examiner will ask again:

(1) Could there be another way of classification?

(2) Please name each group according to the new classification method.

Scoring: 1 point: Cannot complete.

2 points: Can complete part of the item classification (can be coarse or fine).

3 points: Can complete the classification of items twice, but needs prompts and/ or cannot complete all classifications.

4 points: Can complete the classification of items, can have or without prompts, but cannot summarize the classification standard in language.

5 points: Can complete the classification of items and can describe the classification standard in language. If the test subject cannot get the highest score due to language problems, the examiner should explain it next to the score on the scoring form.

21. Riska Object Classification (Unstructured)

Method: This test requires 18 plastic blocks in the test box with 3 different colors (dark brown, light brown, and cream) and 3 different shapes (arrow, ellipse, and 1/ 4 sector). All blocks are randomly placed in front of the test subject. The examiner says: "Group the objects you think are similar." After the test subject completes the grouping, the examiner asks the test subject: "Why are these grouped together?" (or: "What principle did you group by?") When the test subject describes its grouping standards, the examiner asks the test subject: "Now, group in another way."

Note: In order to keep consistent with the overall scoring standards of LOTCA, the scoring standards of the first edition of Riska Shape Classification are reduced.

Scoring:

1 point: Precise pairing (blocks of the same color and shape) and domain collection (arranging the blocks into a house or a flower pattern).

2 points: Classify according to an incomplete standard (for example, some blocks are missed and not grouped, or two standards are confused).

3 points: Arrange the blocks in three dimensions according to a standard (for example, arrange the oval blocks in a row, arrange the arrow blocks on the other side, etc.).

4 points: Randomly classify according to a standard, can switch from one standard to another (for example, first by color, then by shape).

5 points: Can classify according to two or more standards at the same time (for example, combine 2 or more shapes and colors in a group).

22.Riska Object Classification (Structured)

Method: The same plastic blocks as in test item 21 are needed. The examiner places a group in front of the test subject: a dark brown arrow, a cream-colored 1/ 4 sector, and a light brown ellipse. Then the examiner says to the test subject: "I have divided a group, now you start to divide a group similar to mine, try to divide as much as possible." If the test subject can divide all the blocks into groups, the examiner asks again: "What are the similarities between the group you divided and the group I divided?" If the test subject can say three different shapes and three different color standards, the test ends. If the test subject cannot say it, the examiner says again: "The group you divided is similar to the group I divided in some ways, but not in some ways, try to make them more similar." If some groups are not completed, the examiner can give the test subject a hint: "Use all the blocks."

Scoring:

1 point: Precise pairing (blocks of the same color and shape) and/ or collection (arranging the blocks into a line or a flower pattern).

2 points: Classify according to an incomplete standard (for example, some blocks are missed and not grouped, or two standards are confused).

3 points: Classify according to a standard.

4 points: After the examiner's prompt, the second attempt can classify according to two standards at the same time.

5 points: The first attempt can classify according to two standards at the same time.

23 and 24 are picture sorting

The picture sorting story cards (A and B, both purple) in the test box are needed.

23. Picture Sequece A

Method: The examiner spreads 5 cards in front of the test subject in the following order, which together form a small story.

5 2

4 1 3

The examiner asks the test subject:

(1) Please arrange the cards in the correct order.

(2) Please describe the plot of the story.

Scoring: 1 point: Cannot complete.

2 points: Only part of the cards are used, but it does not conform to the whole order.

3 points: Can describe the plot of the story, but the arrangement of the cards is incorrect; or the arrangement of the cards is correct, but cannot describe the plot of the story.

4 points: Correctly completed.

24. Picture Sequence B

If the test subject scores 4 points in Picture Sorting A, or due to aphasia, can only arrange the cards in the correct order, then this picture sorting test is needed. The cards are spread out in the following order:

5 1 4

2 6 3

The requirements for the test subject and the scoring method are the same as in Picture Sorting A test. If the test subject cannot get full marks due to language problems, the examiner should note it on the scoring form. If the test subject scores less than 4 points in the Picture Sorting A test, then the Picture Sorting B test is not needed (this item has no points).

25. Geometric Sequence

The 26th and 27th pages of the test album are needed.

Method: The examiner shows the test subject the first set of geometric sorting shapes (the 26th page of the test album) and gives the test subject a pencil. The examiner says to the test subject: "In this column, these shapes are arranged in a specific order, please continue to draw according to this order." Repeat the test of the second set of sorting geometric shapes (the 27th page of the test album) in this way.

The correct answer to the first set of shape sequences is: circle, square. The correct answer to the second set of shape sequences is: four horizontal line segments, five vertical line segments. In the second set of shapes, if the test subject cannot understand the order of the shapes and cannot draw correctly (for example, the test subject continues to draw according to the beginning of the shape, or continues to draw part of the shape), then the examiner should guide the test subject: "Is there another possible way to continue this shape sequence?"

Scoring: 1 point: Cannot complete.

2 points: Only completes the continuation of the first set of shape sequences.

3 points: After several attempts, can complete the continuation of two sets of shape sequences,

4 points: Can correctly complete shape sorting.

Note: The test subject needs to draw at least two or more shapes that conform to the sorting to get 3 points.

26. Logical Questions

Method: The examiner shows the test subject a page of paper with the following questions, and then reads them together. One question at a time. The test subject can choose to answer orally or in writing according to their own wishes. If the test subject has a language barrier, this test may be difficult.

Questions: (1) Zhang Ming was born in 1930, in which year should he be 35 years old?

(1) Li Da was born in 1950, how old is he this year?

(2) Xiao Li has 5 apples, Xiao Shan has 3 less than Xiao Li, how many apples do they have in total?

(3) Xiao Nan was born earlier than Xiao Zhen, but later than Xiao Sha. Who is the oldest? Who is in the middle? Who is the youngest?

Scoring: 1 point for each correct answer. The lowest score is 1 point, and the highest score is 4 points.

**G. Attention and Concentration**

Method: Score based on the performance of the test subject observed in the above test process.

Scoring:

1 point: The attention concentration period is very short, the test subject's attention does not exceed 5 minutes, and constant repetition is needed.

The test needs to be stopped (the entire test process cannot be completed at once).

2 points: The test subject can concentrate for a short time, the concentration exceeds 15 minutes; repeated prompts are needed. The entire test process needs to be completed in two times.

3 points: The test subject has slight difficulties in attention and concentration, but can still complete all test items after multiple re-concentrations.

4 points: There are no problems with attention and concentration.

**Attachment 4. Glasgow Outcome Scale Extended (GOS-E)**

**Glasgow Outcome Scale Extended (GOS-E)**

| Score | Characteristic |
| --- | --- |
| 8 | Upper Good Recovery, full recovery or minior symptoms that do not affect daily life |
| 7 | Lower Good Recovery, with minor physical or mental deficits that affects daily life |
| 6 | Upper Moderate Disability, some disabilitiy exists, but can partly resume work or previous activities |
| 5 | Lower Moderate Disability, independent but cannot resume work/ school or previous social activities |
| 4 | Upper Severe Disability, needs partial assistance for daily activities |
| 3 | Lower Severe Disability, needs full assistance for daily activities |
| 2 | Vegetative State, absence of awareness of self and environment |
| 1 | Death |

**Attachment 5. Glasgow Coma Scale (GCS)**

**Glasgow Coma Scale (GCS)**

| Eye Opening Response | | Language Response | | Motor Response | |
| --- | --- | --- | --- | --- | --- |
| Item | Score | Item | Score | Item | Score |
| Open eyes spontaneously | 4 | Is oriented to person, place and time | 5 | Follows commands | 6 |
| Open eyes in response to speech | 3 | Converses, may be confused | 4 | Makes localized movement in response to painful stimulation | 5 |
| Open eyes in response to painful stimulations | 2 | Replies with inappropriate words | 3 | Makes non purposeful movements in response to painful stimulation (withdraws from pain) | 4 |
| Does not open eyes in response to any stimulation | 1 | Makes incomprehensible sounds | 2 | Flexes upper extremities/ extends lower extremities in response to painful stimulation | 3 |
|  |  | Makes no response | 1 | Extends all extremities in response to painful stimulation | 2 |
|  |  |  |  | Makes no response to noxious stimulation | 1 |

**Attachment 6. Activities of Daily Living (ADL) Ability Scale (Barthel Index)**

Referencing the Activities of Daily Living (ADL) Ability Scale (Barthel Index) provided by the Disease Control Department of the Ministry of Health and the Neurology Branch of the Chinese Medical Association in the "China Stroke Prevention Guidelines" (trial)

Barthel Index scoring (total score is 100 points): ① Independent 100 points ② Mild dependence 75-95 points ③ Moderate dependence 50-70 points ④ Severe dependence 25-45 points ⑤ Total dependence 0-20 points

**Activities of Daily Living (ADL) Ability Scale**

| Daily Activity Item | Independent | Partially Independent or Requires Some Assistance | Requires Significant Assistance | Completely Dependent | Score |
| --- | --- | --- | --- | --- | --- |
| Eating | 10 | 5 | 0 |  |  |
| Bathing | 5 | 0 |  |  |  |
| Grooming (Washing face, brushing teeth, shaving, combing hair) | 5 | 0 |  |  |  |
| Dressing | 10 | 5 | 0 |  |  |
| Bowel control | 10 | 5（occasional incontinence） | 0（incontinence） |  |  |
| Urinary control | 10 | 5（occasional incontinence） | 0（incontinence） |  |  |
| Toileting (wiping, adjusting clothes, flushing) | 10 | 5 | 0 |  |  |
| Transferring | 15 | 10 | 5 | 0 |  |
| Walking 45 meters on level ground | 15 | 10 | 5 | 0 |  |
| Up and down stairs | 10 | 5 | 0 |  |  |

**Instructions for Filling Out the ADL (BI)**

The BI measures a patient's ten basic daily activities, such as eating, transferring, independently using the toilet, bathing, walking or dressing. Each item is divided into four levels of 0, 5, 10, 15 points according to the difficulty of the task, and the patient is evaluated accordingly. If the patient cannot complete the activity, the score of each item will be determined by the actual time and quantity of help needed. If the patient needs help, even if it is very little help or supervision, he cannot get full marks. When the patient cannot meet the specified standards, it is recorded as 0 points. If the patient gets the highest score (100 points), he should be able to control his bowel movements, eat by himself, get up or leave the chair, bathe independently, walk at least 45 meters, and be able to go up and down stairs. However, this only means that he can be alone, and does not mean that he can live independently (he may not be able to cook or clean the room).

Evaluation Guide

- This indicator should be used to record what the patient has done, not what the patient can do.
- Its main purpose is to determine the degree of independence of the patient in the absence of any help (whether verbal or physical, no matter how small or for what reason).
- In all the items tested, the patient is not independent when supervision is needed.
- The patient's performance should come from the most reliable evidence. It usually comes from the patient's friends, relatives and nurses, but direct observation and consensus are also important.
- Generally speaking, the patient's performance in the previous 24-48 hours is very important, but occasionally it is related to a longer time (for example, last week's bowel movement).
- Patients with unclear consciousness should be scored 0 points, even if there is no incontinence.
- Intermediate means that the patient's efforts exceed 50%.
- If you can control your urination yourself, the medium score means occasional urinary incontinence (less than or equal to 1 time/ 24 hours).
- Allow the use of assistive independent tools (such as crutches).

Use Guide

1. Eating: Independent eating means that the patient can independently eat prepared food within a normal time. The food includes any normal diet (not just porridge), and the food can be made or brought by others; picking up vegetables, serving rice, stirring, cutting food, etc. can be completed independently, scoring 10 points; if a small part of picking up vegetables, serving rice, stirring, cutting food, etc. need help to complete, score 5 points, otherwise score 0 points.

2. Bathing: No need for guidance, supervision and help can enter and exit the bathroom by themselves, wash themselves, shower does not need help or supervision, complete independently, score 5 points, otherwise score 0 points.

3. Grooming: refers to the situation within 24-48 hours, independently complete washing face, combing hair, brushing teeth, shaving and other personal hygiene, caregivers provide tools such as squeezing toothpaste, preparing water, etc, can also score 5 points, otherwise score 0 points.

4. Dressing: refers to being able to put on and take off various clothes, shoes and socks as before the illness, including personal ability to button, open and pull the zipper, wear shoes, etc, score 10 points; need others to help button, shoelaces, open and pull the zipper and other complex functions, but can independently put on outerwear, wear shoes and other simple functions score 5 points, otherwise score 0 points.

5. Bowel control: refers to the situation of a week; can fully control, score 10 points; occasionally (less than or equal to 1 time per week) incontinence, score 5 points; incontinence or coma more than 1 time per week is scored as 0 points.

6. Urinary control: refers to the situation within 24-48 hours; can fully control, score 10 points; occasionally (less than or equal to 1 time per 24 hours, more than 1 time per week urinary incontinence) incontinence, score 5 points; frequent urination (more than 1 time per 24 hours) incontinence, should be scored 0 points. Catheterized patients are classified as urinary incontinence.

7. Going to the toilet: can enter and exit the toilet or stool place by themselves, without others to undress or handle hygiene, score 10 points; if the above activities need help for some functions, score 5 points; if the main functions such as undressing and handling hygiene need help, score 0 points.

8. Chair/ bed transfer: The patient can independently and safely move from the bed to the chair and return, score 15 points; to ensure safety, 1 person needs to help or language guidance, score 10 points; 2 people or 1 strong and skilled person need to help, score 5 points; cannot sit up, or need more than 2 people to help, score 0 points.

9. Walking (walking) on the ground: refers to the ability to use assistive tools (including crutches, but not rolling walking tools such as wheelchairs) to move in the home or ward or hospital, without supervision and care, can walk independently 45 meters, is considered to be completed independently, score 15 points; need 1 untrained person to help (physical or language guidance), including supervision and care, can walk 45 meters, score 10 points; can move independently on a wheelchair, independently use a wheelchair to walk 45 meters score 5 points, if not completed, score 0 points.

10. Up and down stairs: can independently go up and down stairs, including the use of assistive devices (such as crutches) to go up and down stairs, is still considered to be completed independently, score 10 points; under the partial help (such as support) or supervision of others, can complete up and down stairs, score 5 points, otherwise score 0 points.

**Reference**

1 Corrigan, J. D., Selassie, A. W. & Orman, J. A. L. The epidemiology of traumatic brain injury. *J Head Trauma Rehabil* **25**, 72-80 (2010). <https://doi.org/10.1097/HTR.0b013e3181ccc8b4>

2 Faul, M., Xu, L., Wald, M., Coronado, V. & Dellinger, A. Faul, M. , Xu, L. , Wald, M.M. , Coronado, V. & Dellinger, A.M. Traumatic brain injury in the United States: national estimates of prevalence and incidence, 2002-2006. Inj. Prev. 16, A268. *Injury Prevention - INJ PREV* **16** (2010). <https://doi.org/10.1136/ip.2010.029215.951>

3 Cassidy, J. D. *et al.* Incidence, risk factors and prevention of mild traumatic brain injury: results of the WHO Collaborating Centre Task Force on Mild Traumatic Brain Injury. *J Rehabil Med*, 28-60 (2004).

4 Wang, Z. C. & Zhao, Y. L. Craniocerebral trauma clinical research and standardized treatment. (2001).

5 Feigin, V. L. *et al.* Incidence of traumatic brain injury in New Zealand: a population-based study. *Lancet Neurol* **12**, 53-64 (2013). <https://doi.org/10.1016/s1474-4422(12)70262-4>

6 Tagliaferri, F., Compagnone, C., Korsic, M., Servadei, F. & Kraus, J. A systematic review of brain injury epidemiology in Europe. *Acta Neurochir (Wien)* **148** (2006).

7 Peters, J., Daum, I., Gizewski, E., Forsting, M. & Suchan, B. Associations Evoked During Memory Encoding Recruit the Context-Network. *Hippocampus* **19**, 141-151 (2009). <https://doi.org/10.1002/hipo.20490>

8 Fletcher, P. C. & Henson, R. N. Frontal lobes and human memory: insights from functional neuroimaging. *Brain* **124**, 849-881 (2001). <https://doi.org/10.1093/brain/124.5.849>

9 Li, S. C. Neuromodulation of behavioral and cognitive development across the life span. *Dev Psychol* **48**, 810-814 (2012). <https://doi.org/10.1037/a0027813>

10 Chiavarino, C., Apperly, I. A. & Humphreys, G. W. Frontal and parietal lobe involvement in the processing of pretence and intention. *Q J Exp Psychol (Hove)* **62**, 1738-1756 (2009). <https://doi.org/10.1080/17470210802633313>

11 Stoodley, C. J. The cerebellum and cognition: evidence from functional imaging studies. *Cerebellum* **11**, 352-365 (2012). <https://doi.org/10.1007/s12311-011-0260-7>

12 Nachev, P. Cognition and medial frontal cortex in health and disease. *Curr Opin Neurol* **19**, 586-592 (2006). <https://doi.org/10.1097/01.wco.0000247609.36482.ae>

13 Writer, B. W. & Schillerstrom, J. E. Psychopharmacological treatment for cognitive impairment in survivors of traumatic brain injury: a critical review. *J Neuropsychiatry Clin Neurosci* **21**, 362-370 (2009). <https://doi.org/10.1176/jnp.2009.21.4.362>

14 Togher, L. *et al.* INCOG recommendations for management of cognition following traumatic brain injury, part IV: cognitive communication. *J Head Trauma Rehabil* **29**, 353-368 (2014). <https://doi.org/10.1097/htr.0000000000000071>

15 Ponsford, J. *et al.* INCOG 2.0 Guidelines for Cognitive Rehabilitation Following Traumatic Brain Injury, Part II: Attention and Information Processing Speed. *J Head Trauma Rehabil* **38**, 38-51 (2023). <https://doi.org/10.1097/htr.0000000000000839>

16 Son, J. *et al.* Rapid quantitative analysis of oxiracetam in human plasma by liquid chromatography/electrospray tandem mass spectrometry. *J Pharm Biomed Anal* **36**, 657-661 (2004). <https://doi.org/10.1016/j.jpba.2004.07.031>

17 Rozzini, R., Zanetti, O. & Bianchetti, A. Effectiveness of oxiracetam therapy in the treatment of cognitive deficiencies secondary to primary degenerative dementia. *Acta Neurol (Napoli)* **14**, 117-126 (1992).

18 Villardita, C., Grioli, S., Lomeo, C., Cattaneo, C. & Parini, J. Clinical studies with oxiracetam in patients with dementia of Alzheimer type and multi-infarct dementia of mild to moderate degree. *Neuropsychobiology* **25**, 24-28 (1992). <https://doi.org/10.1159/000118805>

19 Yu, D., Jiao, X., Zhou, Y., Chen, Q. & Lou, Y. Human tolerability and pharmacokinetic study of domestically produced oxiracetam capsules. *The Chinese Journal of Clinical Pharmacology* **13**, 6 (1997).

20 He, X. Observation of the therapeutic effects of oxiracetam injection in the treatment of Alzheimer's disease. *Practical Journal of Medicine and Pharmacy* **27**, 1 (2010). <https://doi.org/10.3969/j.issn.1671-4008.2010.05.011>

21 Zhao, G. & Wang, Y. Clinical evaluation of domestically produced oxiracetam in the treatment of dementia. *The Chinese Journal of Clinical Pharmacology* **13**, 5 (1997).

22 Russello, D. *et al.* [Oxiracetam treatment of exogenous post-concussion syndrome. Statistical evaluation of results]. *Minerva Chir* **45**, 1309-1314 (1990).

23 Wei, D., Sun, X., Liu, F. & Tang, W. The effect of oxiracetam on the function of patients with acute mild brain injury. *Journal of Traumatic Surgery* **8**, 3 (2006). <https://doi.org/10.3969/j.issn.1009-4237.2006.03.021>

24 Kai, S. *et al.* Clinical evaluation of oxiracetam capsules in the treatment of traumatic brain injury. *Chinese Journal of Neuromedicine* **4**, 3 (2005). <https://doi.org/10.3760/cma.j.issn.1671-8925.2005.05.014>

25 Wu, S. & Zhan, S. Observation of the therapeutic effects of oxiracetam in 38 cases of acute cranial brain injury. *China Practical Medicine*, 123-124 (2008).
